# Machine learning and semi-empirical calculations: a synergistic approach to rapid, accurate, and mechanism-based reaction barrier prediction†

**DOI:** 10.1039/d2sc02925a

**Published:** 2022-06-14

**Authors:** Elliot H. E. Farrar, Matthew N. Grayson

**Affiliations:** Department of Chemistry, University of Bath Claverton Down Bath BA2 7AY UK M.N.Grayson@bath.ac.uk

## Abstract

Modern QM modelling methods, such as DFT, have provided detailed mechanistic insights into countless reactions. However, their computational cost inhibits their ability to rapidly screen large numbers of substrates and catalysts in reaction discovery. For a C–C bond forming nitro-Michael addition, we introduce a synergistic semi-empirical quantum mechanical (SQM) and machine learning (ML) approach that allows the prediction of DFT-quality reaction barriers in minutes, even on a standard laptop using widely available modelling software. Mean absolute errors (MAEs) are obtained that are below the accepted chemical accuracy threshold of 1 kcal mol^−1^ and substantially better than SQM methods without ML correction (5.71 kcal mol^−1^). Predictive power is shown to hold when the ML models are applied to an unseen set of compounds from the toxicology literature. Mechanistic insight is also achieved *via* the generation of full SQM transition state (TS) structures which are found to be very good approximations for the DFT-level geometries, revealing important steric interactions in some TSs. This combination of speed, accuracy, and mechanistic insight is unprecedented; current ML barrier models compromise on at least one of these important criteria.

## Abstract 2

A synergistic approach that combines machine learning with semi-empirical methods enables the fast and accurate prediction of DFT-quality reaction barriers, with mechanistic insights available from semi-empirical transition state geometries.

## Graphical abstract

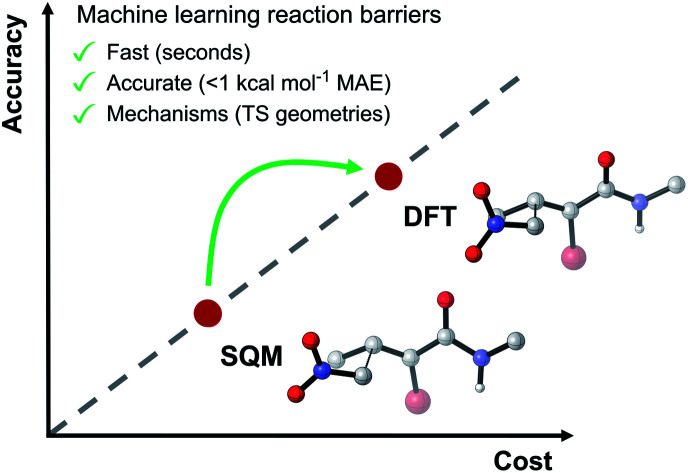

## Introduction

In the last thirty years, the ease-of-use, power, and accessibility of highly sophisticated computational chemistry techniques has increased substantially.^1,2^ These methods allow us to obtain detailed understandings of reaction mechanisms *via* exploration of their potential energy surface (PES) to identify reactant, product, and transition state (TS) structures. Typically, the most telling insights come from analysis of competing TS geometries, from which key steric and electronic effects can be identified, and their respective reaction barriers. This allows us to rationalise the mechanisms and selectivities of a huge range of chemical reactions.^3–6^ In turn, this enables the rational design of new reactions and catalysts,^7–9^ and reduces the need for experimental trial-and-error approaches.

Although numerous toolkits have been developed to automate the location and subsequent optimisation of TS structures,^10–12^ the cost of these methods remains limited by the level of molecular modelling method used in geometry optimisation and energy calculations. Despite some reported shortcomings,^13–15^ for example when TSs contain ion pairs,^16^ density functional theory (DFT)^17,18^ is one of the most widely used quantum mechanical (QM) reaction modelling techniques and has been involved in the successful modelling of countless reactions.^2,19^ However, a trade-off between speed and accuracy must be made; typical DFT calculations take on the order of hours to days, but this can be further exacerbated by the exact combination of functional, basis set and solvation model used, the number of atoms and complexity of the system in question, and the necessity, in most cases, to optimise many distinct chemical species and multiple conformations of each to draw practical conclusions.

In terms of calculation time, molecular mechanics (MM) typically improves on DFT by around six orders of magnitude.^20^ Accordingly, several force field and force field-cost methods have been developed that allow the approximation of various thermochemical properties.^21–31^ However, many do not provide geometric information, or produce less accurate pictures of the TS, for example by treating them as minima. Furthermore, force field methods are often parameterised on niche training domains with expensive DFT calculations or experimental data or require complex and lengthy parameterisation procedures to be effective, limiting their transferability and making their implementation more difficult.

In contrast, most semi-empirical quantum mechanical (SQM) methods are extensively parameterised, and thus widely applicable to many areas of chemistry.^32–35^ Additionally, many are embedded in widely available software packages, such as Gaussian,^36^ GAMESS,^37^ Spartan,^38^ MOPAC,^39^ and ORCA,^40^ and thus are straightforward to implement. Like force fields, SQM methods are several orders of magnitude faster than DFT, on the scale of seconds to minutes per calculation.^20^ However, this comes at the expense of some chemical accuracy as parts of the more expensive QM calculations are replaced with empirical parameters tuned *via* experiment or full QM.^41^ Despite some reported disagreements with more accurate *ab initio* methods, for example in the assignment of Diels–Alder reactions as either step-wise or concerted,^42^ SQM methods have been shown to produce reliable geometries for TSs of many reactions, including nucleophilic substitutions, isomerisations, alkene epoxidations, metal-catalysed oxidations, and even some cycloadditions.^43–45^ However, they require expensive, high-accuracy DFT single point energy (SPE) corrections to give reliable barriers,^43^ and thus SQM data alone is not of a sufficient quality for accurate reaction modelling.

Machine learning (ML) is an increasingly prevalent tool in the field of chemistry, used to identify patterns in chemical datasets.^46,47^ Once a relationship is identified, the target property can be predicted from the features (inputs) of the model. Previously, ML has been used for the prediction of reaction rates and barriers derived from both experiment^48–53^ and high-level reference calculations.^54–63^ However, many models have errors significantly above the accepted threshold for chemical accuracy of 1 kcal mol^−1^,^64,65^ and offer little or no mechanistic insight, for example using molecular fingerprints or graphical representations of molecules. A few examples do make use of TSs in their predictions but use DFT to generate them, making the prediction process very time-consuming.^48,66^ Therefore, no current ML barrier model offers the combination of fast and accurate predictions with mechanistic insight derived from TSs. In recent years, several studies have used ML to bridge the gap between SQM and high-level QM, allowing prediction of various ground state thermochemical properties.^67–71^ We believe this combined SQM/ML approach could be used to improve current standards in the prediction of reaction barriers. Thus, we proposed to learn the relationship between simple, interpretable, and readily available SQM-derived molecular and atomic features and target DFT barriers, and hence afford DFT-quality barriers with the calculation speed of SQM. By calculating TSs using SQM, rapid mechanistic insight would also be available.

**Fig. 1:**
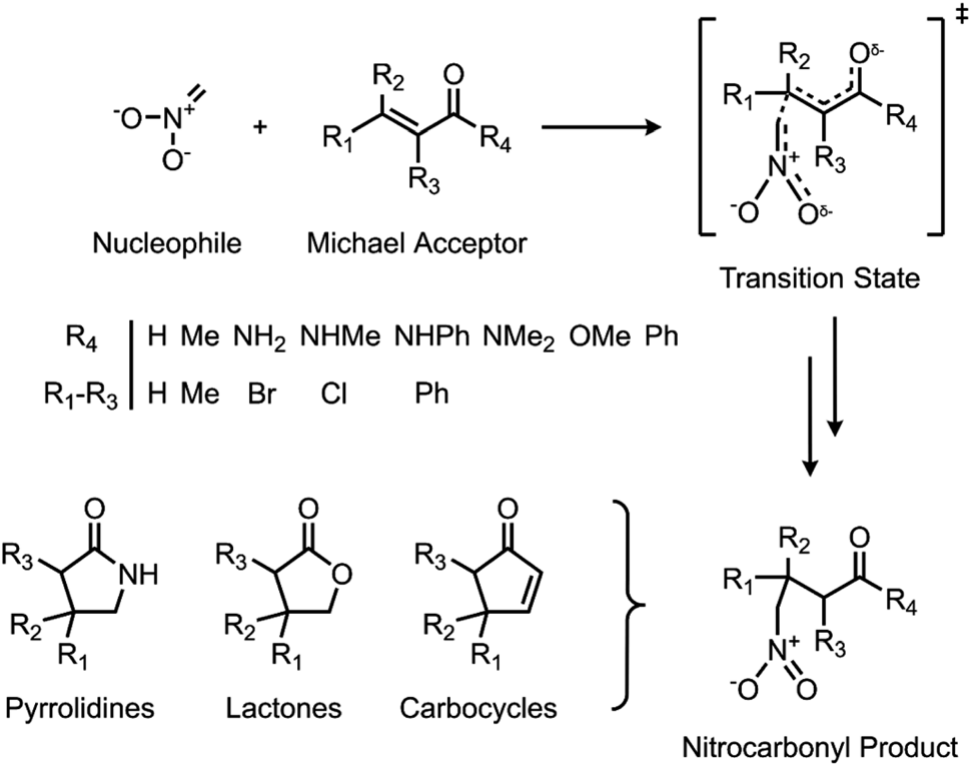
C–C bond forming nitro-Michael additions used to generate the ML dataset.

Herein, we use a synergistic SQM/ML approach to predict DFT-quality free energy activation barriers for a diverse class of C–C bond forming nitro-Michael additions (Fig. 1). Michael additions are one of the most efficient and prevalent methods for formation of C–C bonds in organic and biosynthesis,^72^ finding important applications in asymmetric catalysis^5,73–75^ and several natural product syntheses.^72,76–79^ Among the many classes of these versatile reactions, the nitro-Michael addition is one of the most useful;^80–84^ insertion of the nitro group into the organic framework *via* Michael addition enables a variety of synthetically important stereoselective reactions,^85^ and the resulting nitro compounds can be the precursor for an assortment of highly useful chemical functionalities, including pyrrolidines, lactones, aminocarbonyls, and aminoalkanes.^86^

## Methodology

Reactant and TS geometries for 1000 unique Michael addition reactions were built using Schrödinger's R-Group enumeration^87^ to vary four positions of a generic α,β-unsaturated carbonyl Michael acceptor (MA) core with common organic fragments across synthesis,^79^ toxicology,^88,89^ and covalent drug design (Fig. 1).^90^ A set of 37 additional reactions were built using Michael acceptors (aldehydes, ketones, and esters) from the toxicology literature to be used for external validation (Fig. S2†).^91^ Full details of dataset generation are provided in the ESI, section 1.†

All structures were conformationally searched using Schrödinger's MacroModel (version 12.7)^87,92^ with the OPLS3e force field^93^ before optimising the lowest energy conformation of each with AM1,^32^ PM6,^35^ and ωB97X-D/def2-TZVP^94,95^ using Gaussian16 (Revision A.03).^36^ Additionally, each MA was optimised with UFF for a comparison of SQM with classical force field methods.^96^ To incorporate the effect of solvent, SPE corrections were performed with the same method as the optimisation and the integral equation formalism of the polarisable continuum model (IEFPCM)^97^ with toluene. Temperature (298.15 K) and concentration-corrected (1 mol l^−1^) quasiharmonic free energies were calculated with GoodVibes^98^ and used to calculate reaction barriers (AM1 barrier range: 7.83–42.38 kcal mol^−1^; PM6 barrier range: 2.54–42.01 kcal mol^−1^; DFT barrier range: 3.17–39.35 kcal mol^−1^). Full computational details are provided in the ESI, section 1.†

A variety of simple and interpretable molecular and atomic physical organic chemical features were extracted for each MA and TS at each level of theory (Table S4 and Fig. S7†). Prior to fitting, all features were standardised and processed to deal with collinear and zero-variance features before dividing them into several distinct feature subsets per level of theory (Table S5†); MA features, TS features, and combined MA and TS features (including the reaction barrier) denoted by “All”. Full details of feature extraction are provided in the ESI, section 2.†

The enumerated dataset was randomly split into an 80% train set (800 reactions) and 20% test set (200 reactions) and the former used to train each feature subset to predict the DFT free energy reaction barrier. Seven scikit-learn^99^ regression algorithms were used for training: ridge regression (Ridge), *k*-nearest neighbour regression (NNR), random forest regression (RFR), gradient boosting regression (GBR), support vector regression (SVR), kernel ridge regression (KRR), and Gaussian process regression (GPR). Feature selection and hyperparameter tuning were employed using scikit-learn^99^ and mlxtend^100^ within the 80% train set to prevent overfitting of the feature subsets to the regression models and optimise the models parameters, respectively.^101^ 5-Fold cross validation (CV) was performed within the train set to generate mean absolute errors (MAEs). To assess the individual model performances, external validation was performed using the unseen 20% test set to generate MAEs with standard errors. To further assess the generalisability of the models, MAE scores with standard errors, which are comparable across different sample sizes, were also calculated for the set of 37 unseen reactions from literature. However, among these 37 reactions, two (E5 and E7) contain alcohol groups within their R-groups that allow intra- and intermolecular hydrogen bonding to take place within their respective MA and TS geometries (Fig. S3†). As no such structures were present in the train set, the generated ML models cannot reasonably be expected to learn to account for hydrogen bonding. Indeed, for all models and feature subsets, reactions E5 and E7 were found to exhibit disproportionately worse predictions compared to the MAE of the other 35 structures; for example, absolute errors of 4.26 and 4.74 kcal mol^−1^ were obtained for E5 and E7, respectively, with GPR (AM1 All), compared to an MAE of 0.92 kcal mol^−1^ over the other 35 reactions. Thus, herein, the final literature set was defined as only the 35 structures other than E5 and E7. Full details of the ML process and analyses are provided in the ESI, sections 3 and 4.†

## Results and discussion

**Fig. 2:**
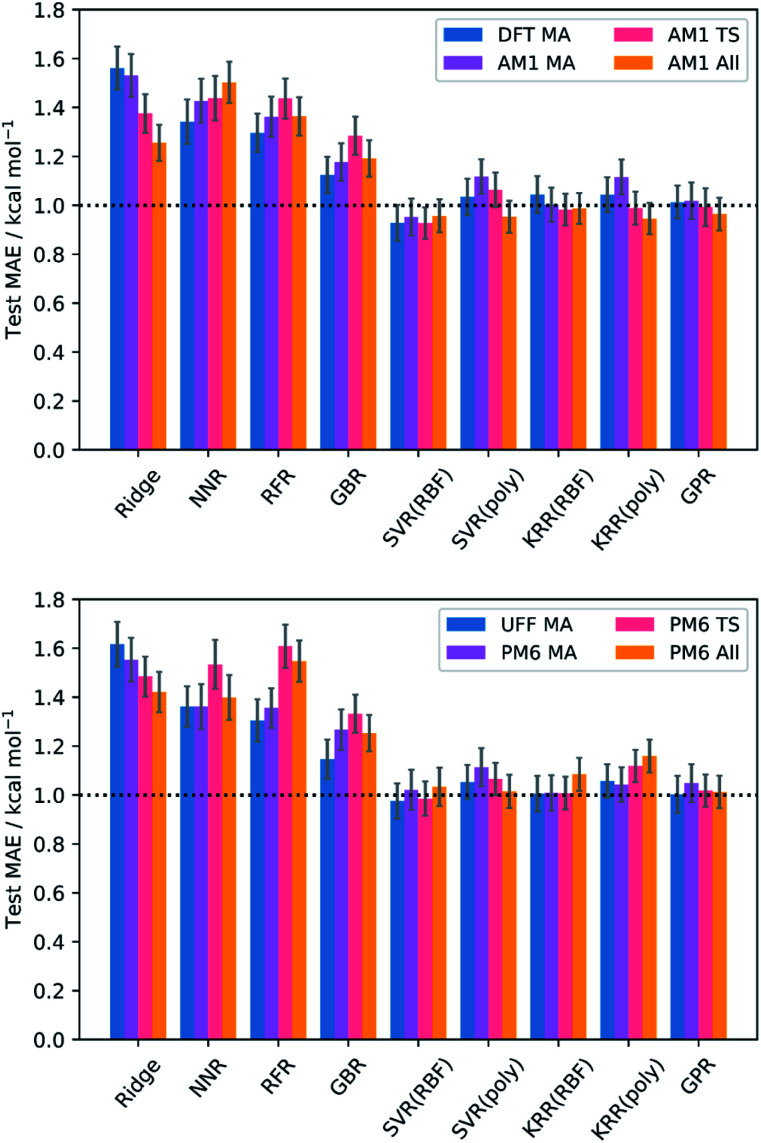
Test MAEs and standard errors (20% test set) for each model and feature subset; RBF = radial basis function kernel; poly = polynomial kernel. GPR used the Matern kernel.

The test MAEs and standard errors (from external validation with the 20% test set) for each model and feature subset are provided in Fig. 2. Full metrics, features, and hyperparameters for each model are provided in the ESI, section 5.† In general, train (5-fold CV) MAEs closely match the test MAEs, indicating that no significant overfitting takes place in the models.

Impressive results are achieved by all models, with test MAEs quenched below 2 kcal mol^−1^ for all feature subsets and each regressor. However, the performances of the kernel-based models (SVR, KRR, and GPR) are the most remarkable, with each producing MAEs below the accepted threshold for chemical accuracy of 1 kcal mol^−1^.^64,65^ Indeed, examples of SVR,^102,103^ KRR,^67,104,105^ and GPR^48,106^ are prevalent in chemistry. These algorithms employ the Kernel trick, mapping the original input features into a higher dimensional feature space in a computationally efficient way and thus allowing the generation of highly complex models at a relatively low cost. In contrast, NNR is a conceptually simple method, and so the complex relationship between the input features and targets cannot be captured as easily. For the remaining models, the data presented does not appear to be of the most suitable form to deliver optimal performance. For example, ridge regression is a linear algorithm that relies on the features having strong linear correlations with the target barriers, which is often not the case in chemical systems;^46^ indeed, only three features across all levels of theory (the AM1 barrier, and the percent buried volume (PBV) of the nucleophile carbon for AM1 and PM6) have linear correlations (Pearson's *r*) with the DFT barrier that are above 0.7, often quoted as the threshold for collinearity.^107^ Similarly, the decision-tree-based models (RFR and GBR) generally lend themselves to the prediction of discrete data, such as reaction selectivities, rather than continuous variables such as reaction barriers.^62,66^ However, despite their apparent unsuitability, each of these models still produces MAEs approaching chemical accuracy, demonstrating the overall success of our SQM to DFT ML approach.

Evaluation of the average performances of the SVR, KRR, and GPR models (Table 1) reveals that all feature subsets produce excellent train and test MAEs. In fact, the predictive power of the MA-only and TS-only feature subsets were generally found to be comparable with the combined subsets. This is important because conformationally searching and obtaining converged structures for reactant states is a more trivial task than for TSs, typically requiring less user input and computational expense. This gives ML users the opportunity to use purely MA-derived features and thus trade off a small amount of accuracy for what could be a substantial amount of time when learning or predicting over enough reactions. However, mechanistic insight from TSs would not be available in such an approach. Additionally, both the MA-only and TS-only feature subsets were found to perform relatively poorly for prediction of the literature structures, with TS features performing slightly better but still worse than their respective train and test metrics. This indicates that TS features are more important than MA features with respect to a model's ability to generalise. However, only when both MA and TS features are combined with the reaction barrier from the SQM method do literature predictions begin to approach the accuracy of the train and test metrics. By inclusion of the reaction barrier, these combined feature subsets represent a version of the Δ-ML approach, in which models are trained to learn the difference between the SQM and DFT barriers, rather than predicting the DFT barrier directly.^67^ Indeed, Δ-ML has previously been found to perform well for out-of-sample predictions.^58^ Therefore, the use of feature subsets without both MA and TS information, as well as the reaction barrier, is generally not recommended.

**Table 1:** Average MAEs of all SVR, KRR, and GPR models

Feature subset	MAE/kcal mol^−1^		
	Train	Test	Literature
UFF MA	0.98	1.02	1.68
AM1 MA	0.99	1.04	1.44
AM1 TS	0.94	0.99	1.17
AM1 All	0.91	0.96	1.03
PM6 MA	0.98	1.05	1.43
PM6 TS	0.99	1.04	1.42
PM6 All	0.98	1.06	1.28
DFT MA	0.95	1.01	1.65

Comparing performances across the different levels of theory, the classical UFF method is found to perform similarly to the AM1, PM6, and DFT MA-only subsets for the train and test sets. However, with only MA information available, extension of UFF to the literature set is poor and mechanistic insight from TSs is not available. In addition to these same drawbacks, the DFT MA subset also suffers from the relatively high cost of the DFT calculations; on a 16-core node, the average DFT MA calculation took over an hour, compared to 5, 14, and 32 seconds with UFF, AM1, and PM6, respectively. Furthermore, DFT calculations scale poorly as the size of the chemical structures become larger. Thus, the use of either UFF or DFT MA features are not generally recommended.

Conceptually, AM1 and PM6 are very similar methods; both are based on the neglect of diatomic differential overlap (NDDO) formalism and share several fundamental approximations, although PM6 is more extensively parameterised and makes several improvements to the core–core potentials.^35^ Accordingly, the initial MAE between the PM6 and DFT barriers (4.17 kcal mol^−1^) was found to be slightly better than between the AM1 and DFT barriers (5.71 kcal mol^−1^). Nevertheless, AM1 was found to perform marginally better after ML, particularly when making predictions on the literature dataset; overall, the combined AM1 MA and TS feature subset (AM1 All) performed the best across the train, test, and literature sets.

**Fig. 3:**
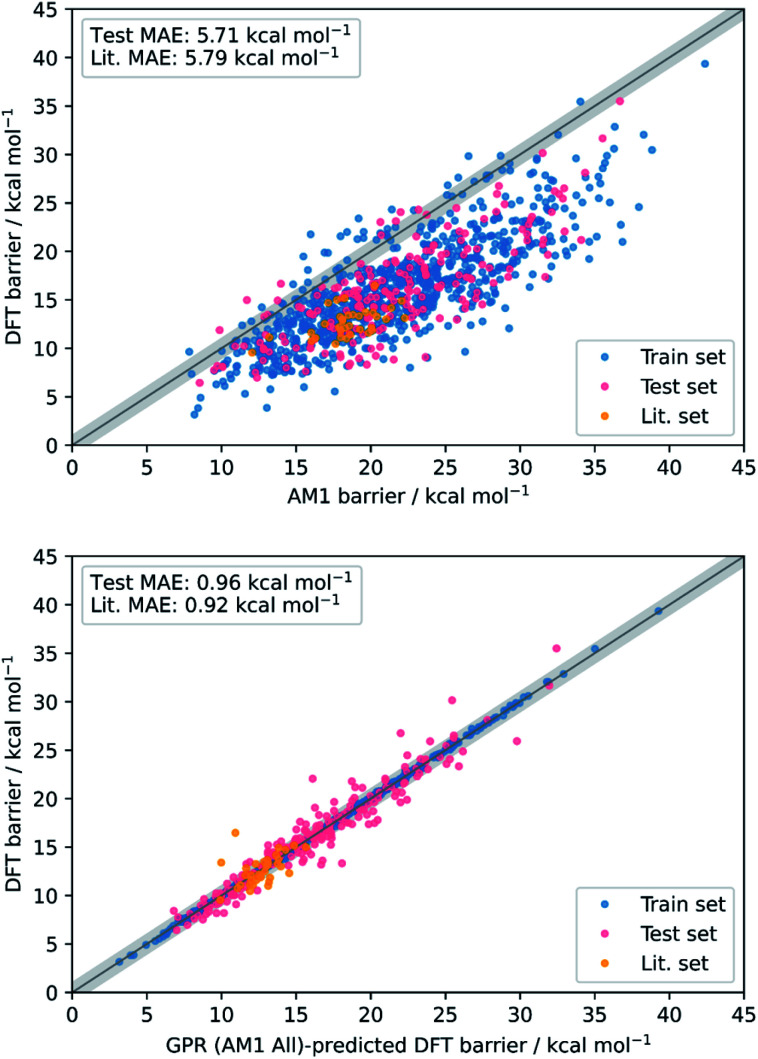
Scatter plots showing the accuracy of DFT barrier prediction using only the AM1 barrier (top) compared to ML with GPR (AM1 All) (bottom), both with respect to the identity line (grey bands correspond to ±1 kcal mol^−1^).

Overall, the best model obtained is *via* GPR using the combined AM1 MA and TS feature subset with 101 features (GPR (AM1 All)), yielding train, test, and literature MAEs of 0.93, 0.96 ± 0.07, and 0.92 ± 0.18 kcal mol^−1^, respectively. Indeed, GPR was found to be the best model in several other studies for the prediction of thermochemical properties,^48,106^ however, using the same feature subset, both KRR(RBF) and KRR(poly) also produced models with all MAEs below 1 kcal mol^−1^. By plotting the AM1 and GPR (AM1 All)-predicted barriers against the DFT barriers, the improvement gained over the untrained model *via* our ML approach with respect to both the test set and literature set predictions can be visualised (Fig. 3).

**Fig. 4:**
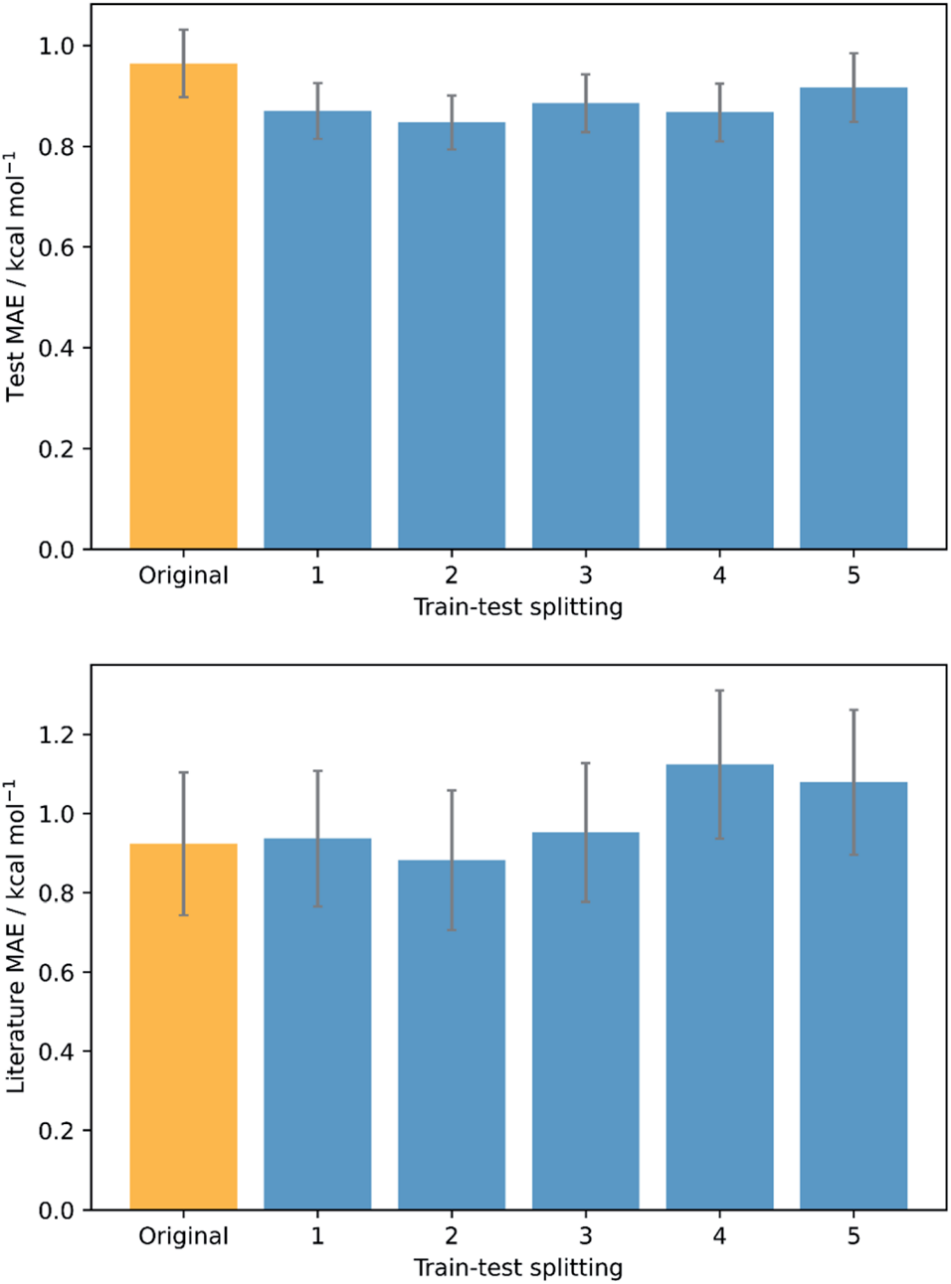
Double cross validation; test (top) and literature (bottom) MAE and standard errors for GPR (AM1 All) at the original test–train splitting (yellow) and five additional splittings (blue).

**Fig. 5:**
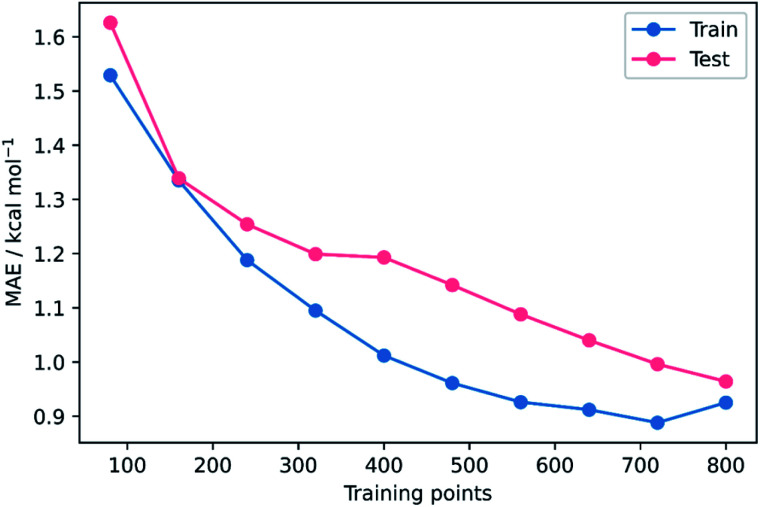
MAE learning curves for GPR (AM1 All).

To validate that the success of the GPR model is genuine and not due to any fortuitous test–train splitting, we performed an extensive double CV approach by retraining the model at five additional random test–train splittings (Fig. 4).^108^ These produced average train, test, and literature MAEs of 0.94, 0.89 ± 0.06, and 0.98 ± 0.18 kcal mol^−1^, respectively, in line with the original metrics. Additionally, learning curves for the model (Fig. 5) flatten out as the number of training points is increased towards the maximum of 800, indicating that the size of the train set is acceptable and does not substantially limit the accuracy of the model or its ability to make predictions. Finally, the train and test scores tend to be very similar, indicating that no significant overfitting takes place in the model at any point. Learning curves for the SVR and KRR models with the AM1 All feature subset were found to display the same trends (see ESI, section 6†).

**Fig. 6:**
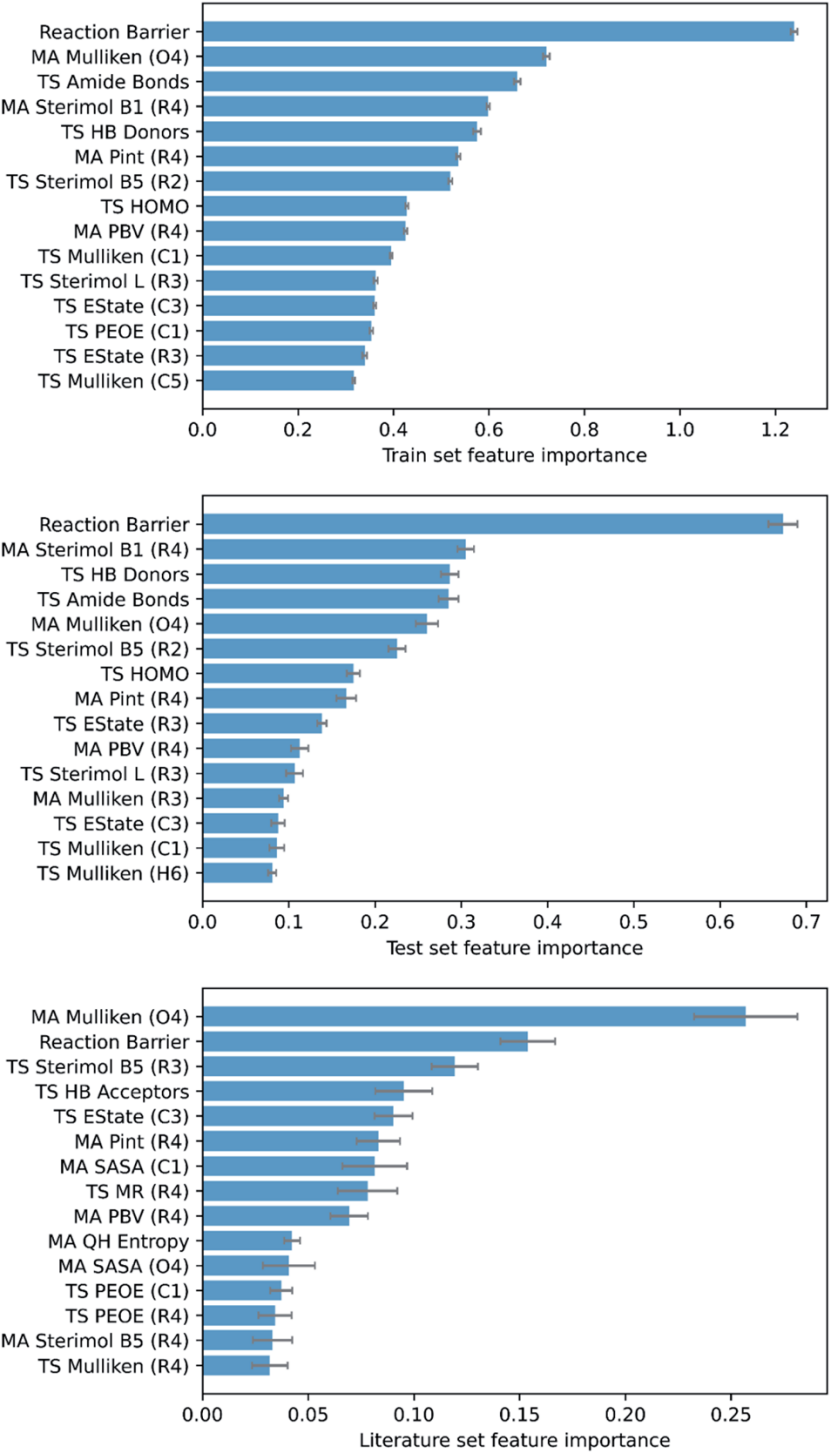
Top 15 train, test, and literature set permutation features importances for GPR (AM1 All).

To ascertain which features make the largest contributions to determining the DFT barrier, as well as the overall generalisability of the models, their permutation feature importances were analysed. This process, in a typical ML study, can provide useful chemical insights into the mechanism of the reaction, depending on the interpretability of the highest-ranking features. For the GPR (AM1 All) model, the AM1 barrier was found to rank very highly in feature importance (Fig. 6).

The next most important feature, and the most important for the literature set, is the Mulliken charge of the carbonyl oxygen. The conjugated π-system of α,β-unsaturated carbonyls is soft and highly polarisable, whilst oxygen is a highly electronegative atom.^109^ Thus, the charge of the oxygen is a good indicator of the total electron density in the π-system of each MA. Accordingly, as the charge of the oxygen becomes more negative, interaction between the MA and negatively charged nucleophile becomes more difficult, and the reaction barrier increases. In addition, several other features encoding electrostatic information for specific atoms (largely the R_1_–R_4_ substituents and core Michael acceptor atoms, C_1_–O_4_) are important, including orbital electronegativities (PEOE), dispersion descriptors (*P*_int_), and electrotopological characteristics (EState). Overall, these features account for a significant proportion of the electrostatic component of the nitro-Michael addition reaction.

Finally, several steric features, including sterimol parameters, PBVs, and solvent accessible surface areas (SASA), comprise a substantial proportion of the remaining high-importance features. This is in line with previous findings that a significant extent of steric control exists in the rate of Michael addition reactions with glutathione.^89,110^ For example, steric parameters in the vicinity of the α,β-unsaturated carbonyl describe the steric accessibility of the β-carbon, where the nucleophile needs to be for 1,4-Michael addition to occur, and thus correlate very strongly with the reaction barrier.

The top-performing features of GPR models with subsets derived from PM6 calculations, in addition to each of the SVR and KRR models with AM1 features, were of a similar nature to GPR (AM1 All) (see ESI, section 7†), validating the importance of the features discussed. However, whilst chemical insights can be drawn from these features, allowing a subsequent understanding of their impact on the reaction mechanism, one of the unique benefits of our ML approach is that full SQM TS geometries are produced as part of the feature generation process. In turn, this allows mechanistic insights into the reaction to be obtained *via* visualisation of the geometries, without the need to analyse individual feature importances. But how accurate are these SQM geometries, and can they be used as approximations for the DFT TS geometries?

**Fig. 7:**
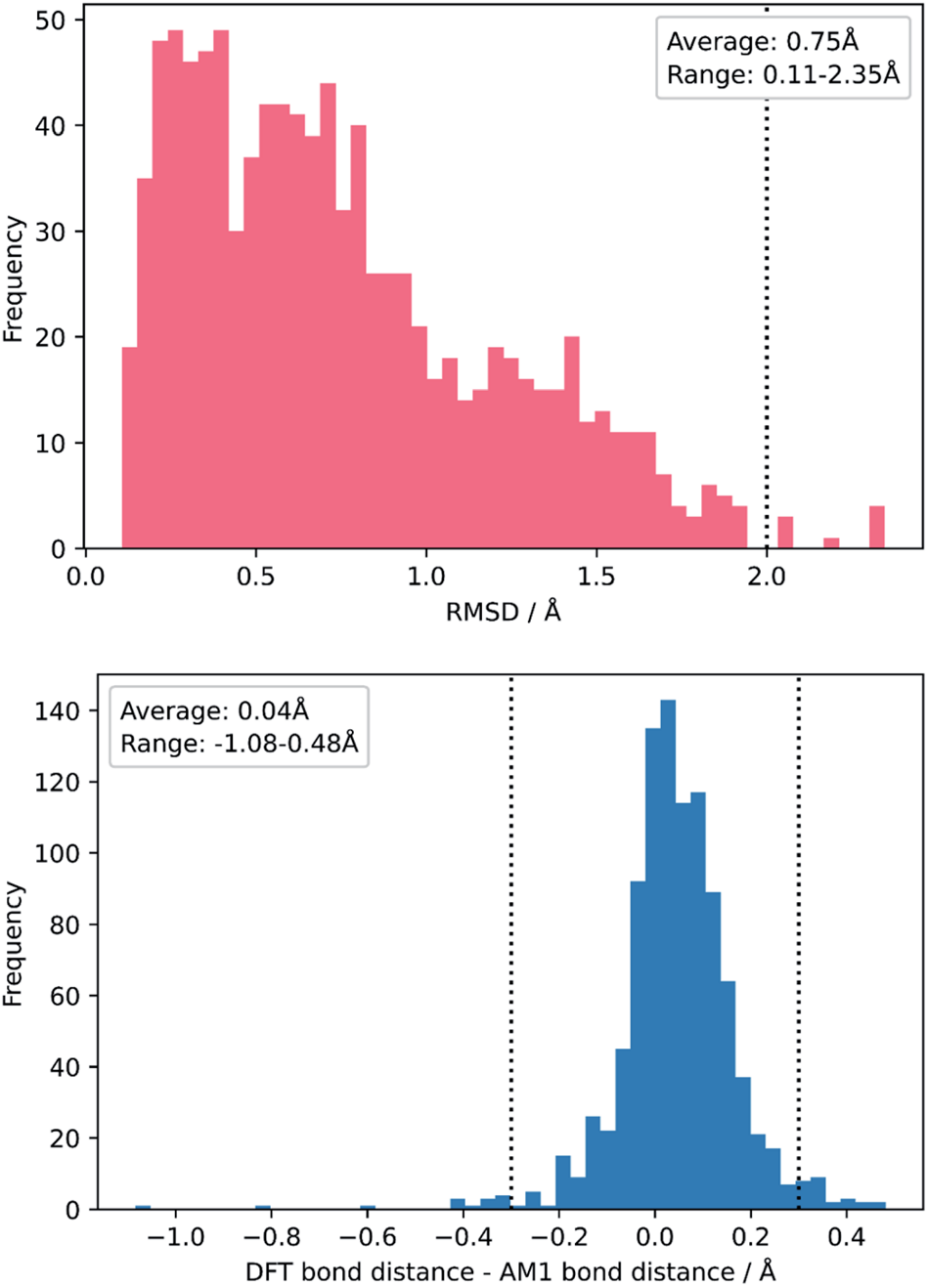
Distribution of RMSDs (top) and bond distance differences (bottom) between each TS in the enumerated dataset at its AM1 and DFT geometries.

**Fig. 8:**
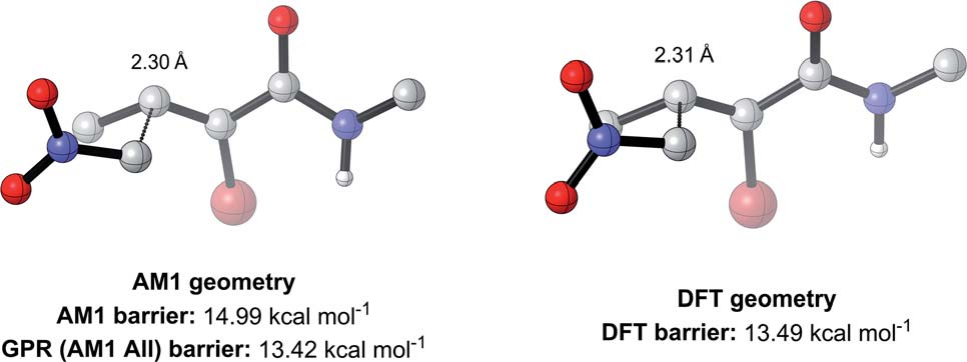
AM1 and DFT geometries of the enumerated TS with the RMSD closest to the average of 0.75 Å (hydrogens omitted for clarity).

To test this, we calculated the root-mean-squared deviation of atomic positions (RMSD)^111^ and difference in bond-forming distance between each of the TS geometries at the AM1 and DFT levels of theory (Fig. 7). In the context of molecular docking, an RMSD below 2 Å is considered successful when comparing the conformations of organic ligands to their protein-bound conformation.^112^ The average RMSD for our enumerated dataset of 1000 structures was calculated at 0.75 Å, with 99.2% of TSs falling below the 2 Å threshold. The bond-forming distance is on average 0.04 Å larger in the DFT structure than in the AM1 structure, with 96.4% of TSs having an absolute difference in bond-forming distance below 0.3 Å. Fig. 8 depicts the AM1 and DFT geometries of the TS with the RMSD closest to 0.75 Å, and hence represents the approximate average deviation that would be expected between an AM1 and DFT geometry in our dataset. Close inspection of the structures reveals that the major origin of deviation results from the angle of approach of the nucleophile and the orientation of R-groups, rather than any changes to the core structure of the MA. Accordingly, removing the nucleophile or the R-groups from each structure and recalculating the RMSDs drops the averages to 0.6 Å and 0.45 Å, respectively, whilst removing both (leaving only the core enone functionality) drops it to 0.14 Å (Fig. S20–22†). In fact, even when the AM1 MA geometries were compared to the DFT TS geometries with the nucleophile removed, an average RMSD of 1.35 Å was calculated, substantially below the 2 Å threshold (Fig. S23†). In all cases, similar distributions and average values were also calculated for the literature structures and at each level of theory (see ESI, section 8†); notably, UFF and DFT MA geometries were similarly comparable with DFT-derived TS geometries (nucleophile removed), whilst PM6 was found be slightly worse at predicting DFT TS geometries than AM1, with a larger average RMSD of 0.87 Å.

The inclusion of solvent *via* SPE corrections results in highly flexible models that allow different solvents to be incorporated without reoptimising every structure. However, to examine the impact of solvent, AM1, PM6, and DFT TS optimisations with the IEFPCM solvent model were performed on the literature set. We then superimposed each pair of gas and solvent optimised TSs and calculated average RMSDs of 0.13 Å for AM1, 0.30 Å for PM6, and 0.16 Å for DFT (Fig. S27 and S28†). These low RMSDs indicate that, for the reaction investigated in our work, the use of gas phase optimised structures is a valid approximation.

**Fig. 9:**
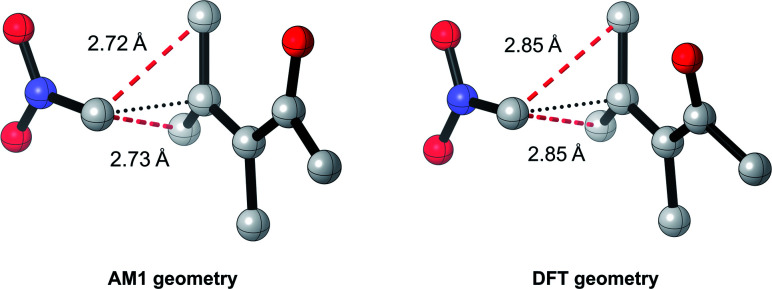
AM1 and DFT geometries of an example TS revealing steric interactions between the nucleophile and the R_1_ (Me) and R_2_ (Me) groups of the MA; C–C distances within 90% of the sum of the van der Waals radii (3.4 Å) are indicated by dotted red lines.

Overall, these analyses indicate that SQM geometries of TSs, and even MAs to some extent, can be considered good substitutes for the full DFT level geometries, on average, allowing accurate mechanistic analysis of the TSs simply by their visualisation. Importantly, no other ML barrier model that we are aware of offers this level of mechanistic insight without the need for time-consuming DFT calculations. For example, analysis of one TS structure at the DFT level reveals how severe C–C steric interactions between the nucleophile and the R_1_ and R_2_ groups of the MA destabilise the structure, and these interactions are also captured by the AM1 geometry (Fig. 9). Whilst insights into the electronics and sterics of the reaction were revealed by analysis of the most important predictive features, direct visualisation of the geometries and interactions improves upon this approach by identifying which groups are directly involved in this steric clash and revealing more about their exact nature. Such information helps to validate the predictions made by ML and can guide rational reaction design.

For the nitro-Michael addition reaction investigated here, the full DFT level of theory took approximately 7 hours on a 16-core node to obtain each of the 1000 enumerated MA and TS structures, compared to only 51 seconds for the respective AM1 calculations (Table S3†). On a 1-core laptop, this corresponds to over 100 hours of calculation for DFT per structure, compared to less than 15 minutes for AM1. Thus, the ease and efficiency of our SQM/ML approach is demonstrated; with a prebuilt ML model, the user simply calculates a baseline barrier for a reaction (in seconds, using widely available SQM or force field approaches), applies a correction *via* ML (in seconds), and accurate DFT-level barriers and geometries can be obtained. Although users may be concerned that the deficiencies of a particular baseline method, for example poor applicability to a particular chemical domain (see introduction), may inhibit a model's ability to make accurate predictions, our results demonstrate that feature subsets derived from both classical force field and SQM methods all lead to comparable predictive performances. Thus, the user may simply select an appropriate baseline method for the reaction in question and, by the same principles described above, accurate predictions should be possible. For a more detailed account of the applicability of SQM methods in modelling organic chemistry, we recommend a review by Thiel.^113^

Overall, the combination of highly accurate SQM geometries and ML-derived energies represents a significantly cheaper way to obtain very good approximations of DFT-level geometries and energies. In turn, this enables the rapid prediction of reaction barriers and delivers mechanistic insight for this essential class of nitro-Michael additions, which could lead to much faster screening of these kinds of reactions, and thus much more efficient design of new synthetic methodology.

## Conclusion

We have combined ML and SQM calculations to achieve the fast and accurate prediction of DFT-quality free energy activation barriers using widely available computational techniques. Using a variety of regression algorithms with simple and highly interpretable features, MAEs below the accepted chemical accuracy threshold of 1 kcal mol^−1^ were achieved with a calculation time of seconds, even when making predictions on an unseen set of compounds from the toxicology literature. Evaluation of the most predictive features provided clear insights into important aspects of the nitro-Michael addition reaction mechanism. However, SQM geometries of TSs, and to some extent MAs, were found to be very good approximations to the full DFT TS geometries and thus offer mechanistic insight with no additional work required. Combination of these SQM geometries with highly accurate ML-derived energies allows the prediction of barriers and the screening of reactions at DFT level, without the need for time-consuming DFT calculations. No current ML barrier models offer our combination of speed, accuracy, and mechanistic insight. The generalised nature of the study means the ML approach can be highly customised, for example by choosing from various regression algorithms, features, and molecular modelling methods. We believe that the same principles could also be applied to achieve the rapid prediction of reaction barriers and mechanisms for other important classes of chemical reactions, paving the way for more efficient drug discovery and rational reaction design.

## Data availability

Gaussian16 output files for all computed structures are openly available in Dataset for "Machine learning and semi-empirical calculations: a synergistic approach to rapid, accurate, and mechanism-based reaction barrier prediction" in the University of Bath Research Data Archive at https://doi.org/10.15125/BATH-01092.

## Author contributions

This manuscript was written through contributions from all authors.

## Conflicts of interest

There are no conflicts to declare.

## Supplementary Material

SC-013-D2SC02925A-s001
