# Supplementary material for: Machine learning and semi-empirical calculations: a synergistic approach to rapid, accurate, and mechanism-based reaction barrier prediction
Source: Chem Sci. 2022 Jun 14;13(25):7594–603. doi: 10.1039/d2sc02925a (PMC9242013; doi:10.1039/d2sc02925a)
Supplement: SC-013-D2SC02925A-s001 [file SC-013-D2SC02925A-s001.pdf]

Electronic Supplementary Material (ESI) for Chemical Science.
This journal is © The Royal Society of Chemistry 2022

Machine learning and semi-empirical calculations: A synergistic approach to rapid, accurate and mechanism-based reaction barrier prediction

Supporting Information

*Elliot H. E. Farrar and Matthew N. Grayson**

Department of Chemistry, University of Bath, Claverton Down, Bath, BA2 7AY (UK)

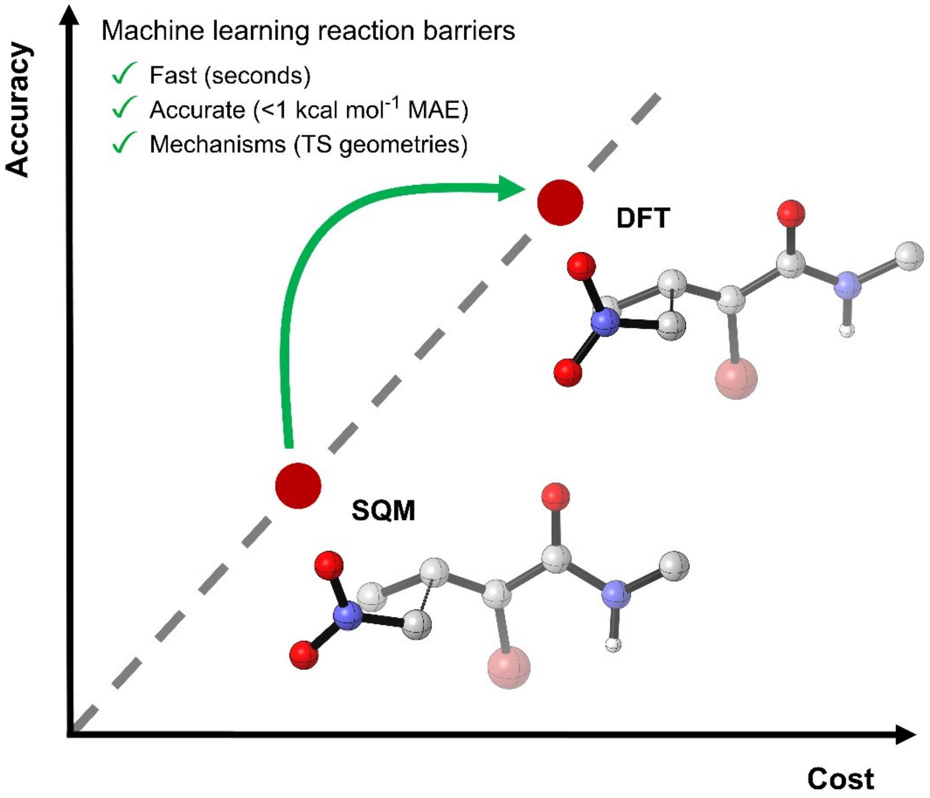

Table of Contents

1. Dataset Generation 2
2. Feature Extraction 6
3. Machine Learning 8
4. Model Analysis 9
5. All Machine Learning Metrics, Features, Hyperparameters 10
6. Learning Curves 32
7. Feature Importances 34
8. RMSD Analyses 40
9. References 43

1. Dataset Generation

Initial geometries were built for 1000 unique nitro-Michael addition reactions by varying the organic fragments at four positions of a generic Michael acceptor (MA) core using the Custom R-Group Enumeration feature of Schrödinger's Maestro (release 2020-1)[1] (Fig. S1). Thus, a total of 2001 structures were obtained; the nucleophile (MW: 60), 1000 MAs (MW: 56-380.8), and 1000 TSs for reaction of the nucleophile with each MA (MW: 116-440.8). R-groups were selected to be representative of common fragments across synthesis,[2] toxicology,[3] and covalent drug design.[4]

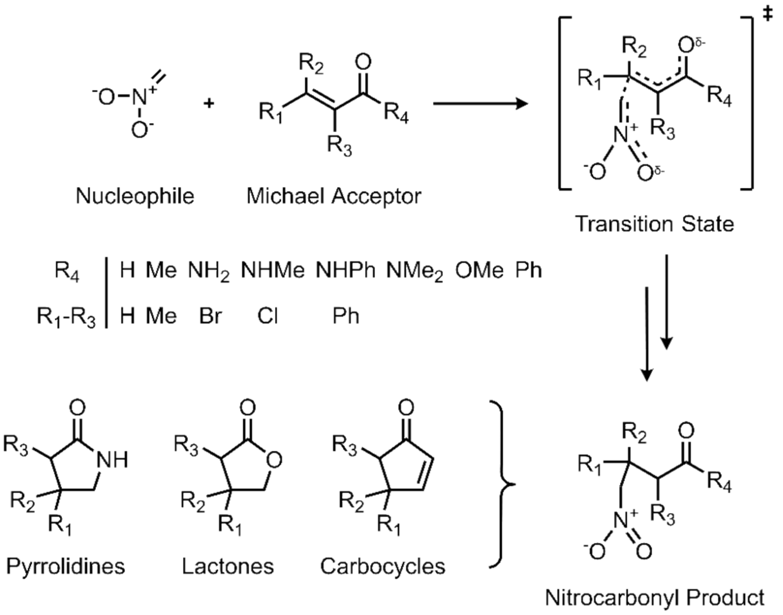

Fig. S1. C-C bond forming nitro-Michael addition used to generate the machine learning dataset.

A further 37 nitro-Michael addition reactions were generated using a set of MAs (aldehydes, ketones, and esters) from the toxicology literature (Fig. S2), leading to an additional 37 MAs (MW: 80-156.1) and 37 TSs for reaction of the nucleophile with each MA (MW: 142-216.1).[3] Where two or more C=C double bonds were present, reactions were always calculated at the β-carbon. In the original literature publication, 58 MAs were selected due to the potential impact of their reactivity in toxicity. From these 58 structures, 12 structures (coloured blue) that were already present in the enumerated dataset of 1000 reactions were removed to avoid bias when performing external validation. An additional 9 structures (coloured red) bearing triple bonds as part of the α,β-unsaturated carbonyl functional group were removed, as these represent a different form of reactivity that the ML models are not trained to predict. A previous study by Schwöbel found that separating α,β-unsaturated carbonyls with double and triple bonds is a valid approach when building models to predict the reactivity of MAs with glutathione.[5] Among the remaining 37 MAs, two reactions, E5 and E7, contain alcohol groups within their R-groups that allow intra- and intermolecular hydrogen bonding to take place in their respective MA and TS geometries (Fig. S3). As no such reactions are present in the enumerated dataset of 1000 reactions, the generated ML models cannot reasonably be expected to learn to account for hydrogen bonding. Indeed, for all models and feature subsets, reactions E5 and E7 were found to exhibit disproportionately worse predictions compared to the MAE of the other 35 structures; for example, absolute errors of 4.26 and 4.74 kcal mol^{-1} were obtained for E5 and E7, respectively, with the GPR model with the AM1 All feature subset, compared to an MAE of 0.92 kcal mol^{-1} over the other 35 reactions. Thus, the 37 literature reactions were divided in two distinct sets to assess the predictive performance of the generated ML models with and without hydrogen bonding and alcohol groups:

- Literature set 1: 35 structures (E5 and E7 removed).
- Literature set 2: 37 structures (E5 and E7 included).

Thus, a total of 1037 nitro-Michael addition reactions were generated. Except where specified, any reference to the "literature set" refers to literature set 1 (35 structures (E5 and E7 removed)). The average train, test, and literature (all sets) MAEs of the SVR, KRR, and GPR models for each feature subset are provided in Table S1. For the two literature sets, predictions are always best when E5 and E7 are omitted (literature set 1).

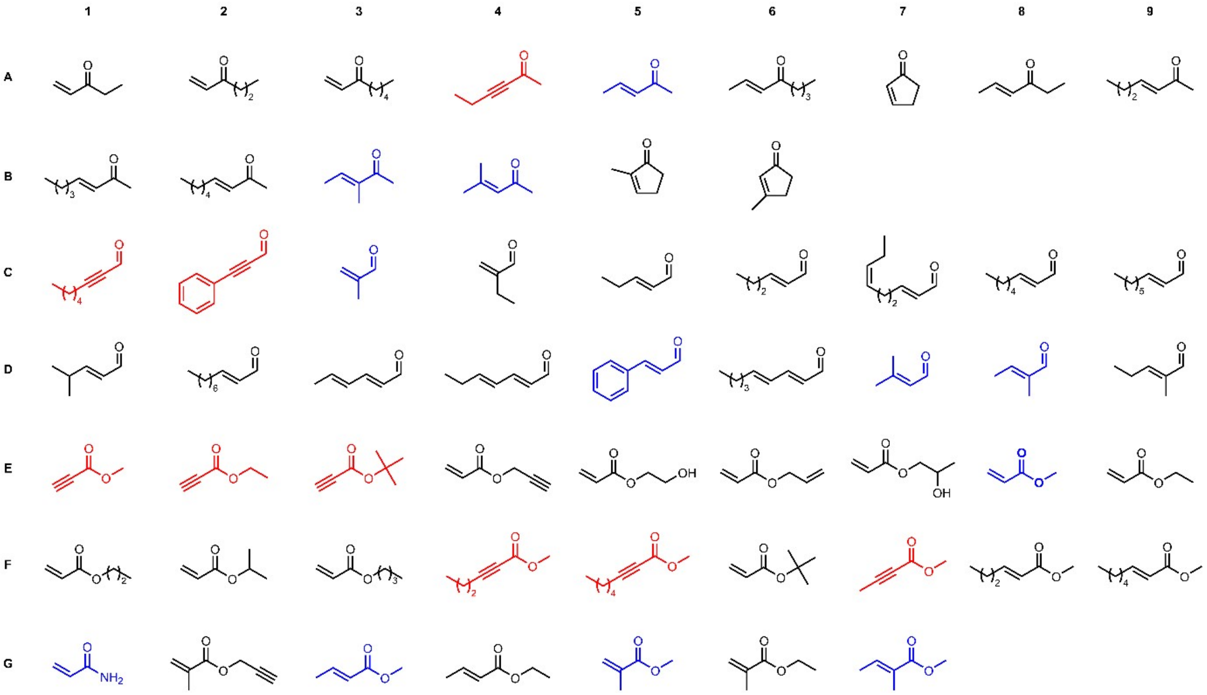

Fig. S2. 58 Michael acceptors used to build reactions in the literature dataset; all blue structures, which were already present in the enumerated dataset, and red structures, which contain triple bonds as part of the α,β-unsaturated

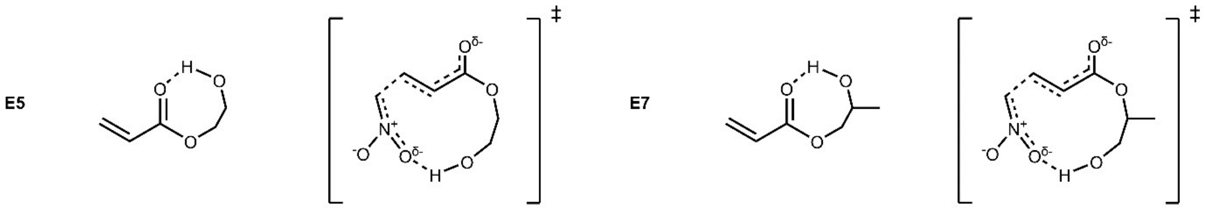

carbonyl functional group, were removed, leaving 37 structures in total.

Fig. S3. Hydrogen bonding in the MA and TS for reactions of E5 and E7.

For each reaction, temperature (298.15 K) and concentration-corrected (1 mol/l) quasiharmonic (Grimme approximation)[6] free energies were calculated using the GoodVibes python package,[7] with vibrational scaling factors of 0.975[8] for ωB97X-D/def2-TZVP and 1 for all other levels of theory. These were used to calculate free energy reaction barriers (Table S2) by equation (1). Fig. S4 illustrates the generally continuous spread of reaction barriers which are approximately normally distributed for each level of theory according to D'Agostino-Pearson tests.[9,10]

$$(1)\ \Delta G^{\ddagger} = G_{TS} - (G_{MA} + G_{nuc})$$

All structures were conformationally searched using the conformational search tool within Schrödinger's MacroModel (version 12.7)[11,12] with the OPLS3e force field.[13] A recent comparison of several force fields found OPLSe to perform the best when predicting the ordering of conformers of organic molecules based on their DFT energy.[14] A mixed Monte Carlo Multiple Minimum (MCMM)[15] and low-mode sampling approach[16,17] was used to explore the possible conformations of each structure. The lowest energy conformation of each structure, based on its OPLS3e energy, was subsequently optimised with several baseline (molecular mechanical (MM) and semi-empirical quantum mechanical (SQM)) methods and one targetline (density functional theory (DFT)) method using Gaussian16 (Revision A.03).[18]

For the baseline methods, the Universal Force Field (UFF),[19] Austin Model 1 (AM1),[20] and Parameterisation Method 6 (PM6)[21] were used; these methods are widely available in QM packages such as Gaussian. Additionally, AM1 and PM6 represent two of the more modern and better parameterised general purpose SQM methods available.[21,22] The newer PM7 method,[23] when tested on a large subset of our structures, regularly failed to reach convergence. For the DFT calculations, the long-range corrected ωB97X-D functional[24] was used with the polarised triple-ζ valence quality (def2-TZVP) basis set.[25] These types of functional have been found to perform very well for prediction of barrier heights,[26,27] and similar methods have previously been used with success in large scale generation of chemical reaction datasets.[28] For all methods, single point energy (SPE) calculations[29] were performed with the same method as the optimisation but with the addition of the integral equation formalism of the polarisable continuum model (IEFPCM)[30] with toluene.

Toluene is a widely used solvent in hydrogen-bonding catalysis and was thus selected for any calculations incorporating solvent.[31,32] To verify that the models are also predictive of geometries in solvent, the 37 reactions from the literature were also reoptimised with AM1/IEFPCM(toluene)[30] and ωB97X-D/def2-TZVP/IEFPCM(toluene). An in-house python package was used to automatically manage the calculation workflow. All calculations were performed on a High Performance Computing (HPC) architecture using 12-24 cores and one node; the approximate average time of each set of calculations (on a 16-core node) are summarised in Table S3. Gaussian16 output files for all computed structures are openly available in *Dataset for "Machine learning and semi-empirical calculations: A synergistic approach to rapid, accurate, and mechanism-based reaction barrier prediction"* in the University of Bath Research Data Archive at https://doi.org/10.15125/BATH-01092. Computed structures were illustrated in the manuscript with CYLView.[33]

**Feature Subset**	**MAE / kcal mol^{-1}**			
	**Train**	**Test**	**Lit. 1**	**Lit. 2**
**UFF MA**	0.98	1.02	1.68	1.91
**AM1 MA**	0.99	1.04	1.44	1.63
**AM1 TS**	0.94	0.99	1.17	1.36
**AM1 All**	0.91	0.96	1.03	1.25
**PM6 MA**	0.98	1.05	1.43	1.63
**PM6 TS**	0.99	1.04	1.42	1.55
**PM6 All**	0.98	1.06	1.28	1.47
**DFT MA**	0.95	1.01	1.65	1.83

Table S1. Average MAEs of all SVR, KRR, and GPR models.

**Feature Subset**	**Enumerated Dataset**	**Literature Dataset**
AM1	7.83-42.38	12.04-22.26
PM6	2.54-42.01	12.95-21.92
DFT MA	3.17-39.35	9.56-16.47

Table S2. Barrier ranges (kcal mol^{-1}) for each level of theory across the enumerated and literature datasets.

	**Enumerated Dataset**		**Literature Dataset**	
**Feature Subset**	**Optimisation**	**SPE**	**Optimisation**	**SPE**
UFF MA	00:00:05	00:00:03	00:00:04	00:00:02
AM1 MA	00:00:14	00:00:02	00:00:09	00:00:03
AM1 TS	00:00:37	00:00:03	00:00:37	00:00:05
PM6 MA	00:00:32	00:00:05	00:00:11	00:00:04
PM6 TS	00:01:00	00:00:07	00:00:46	00:00:05
DFT MA	01:11:34	00:04:54	01:10:14	00:02:19
DFT TS	05:41:27	00:09:59	03:47:10	00:04:55

Table S3. Approximate average calculation times on a 16-core node (hours:minutes:seconds).

The enumerated dataset of 1000 reactions was randomly split into an 80% train set (800 reactions) and 20% test set (200 reactions) using the Scikit-learn (sklearn) python package.[34] The relationships between the SQM and DFT reaction barriers for each level of theory are summarised in Fig. S5-6. In each case, the mean absolute error (MAE) between the SQM and DFT barriers are substantially above the accepted threshold for chemical accuracy of 1 kcal mol^{-1}.[35,36]

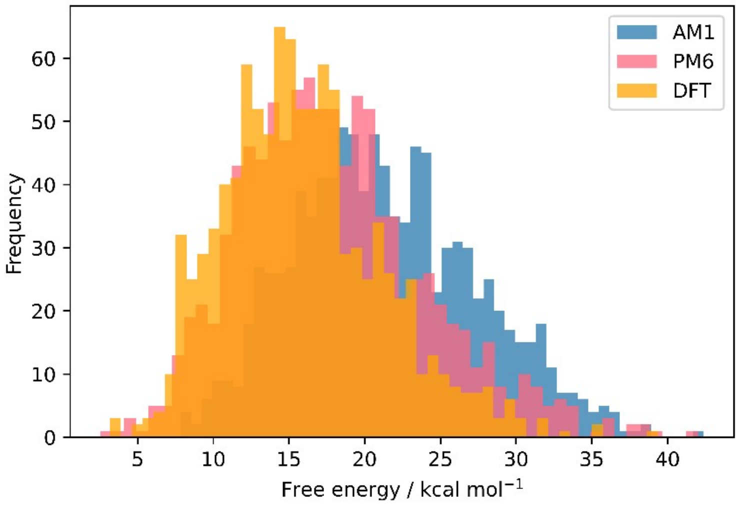

Fig. S4. Reaction barrier distribution for the enumerated dataset of 1000 reactions.

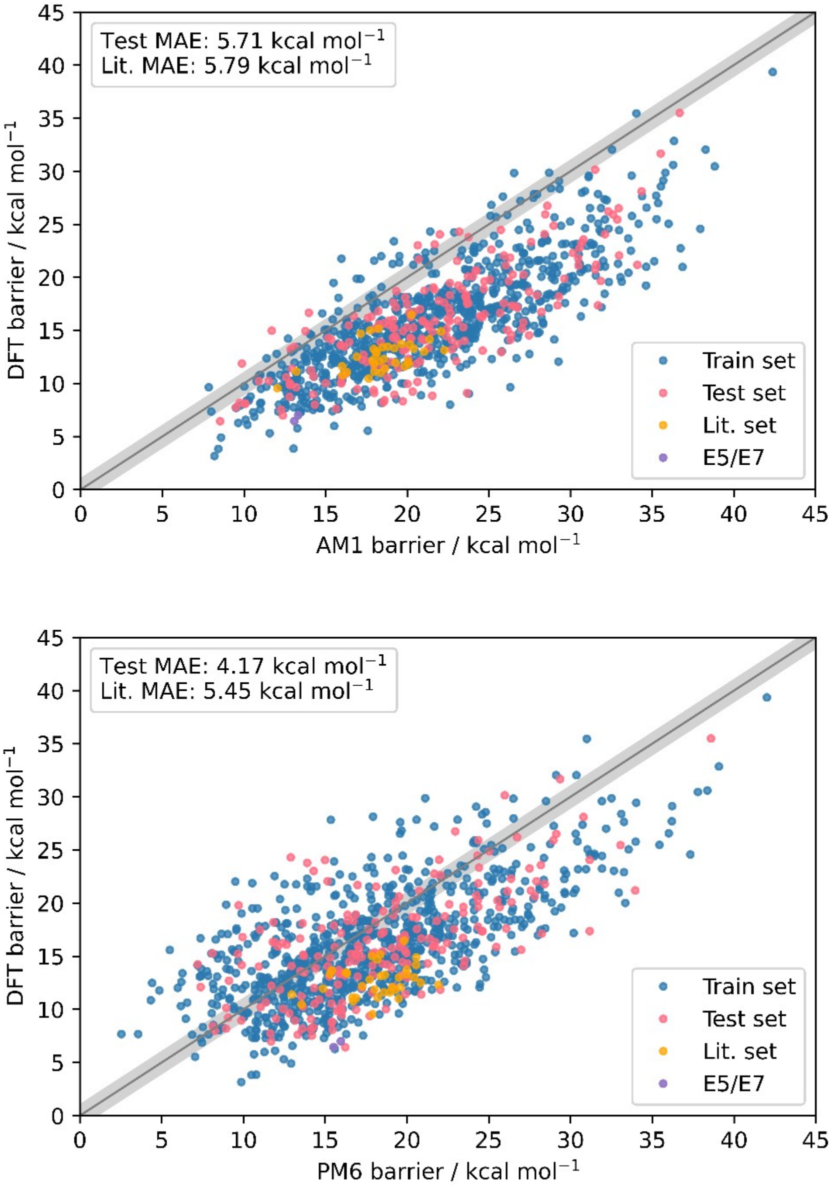

Fig. S5. AM1 and DFT barriers with respect to the identity line (grey band corresponds to ± 1 kcal mol^{-1}).

Fig. S6. PM6 and DFT barriers with respect to the identity line (grey band corresponds to ± 1 kcal mol^{-1}).

2. Feature Extraction

A variety of physical organic features were extracted for the 1037 MAs and TSs at all levels of theory using a series of python packages (Table S4, Fig. S7). To avoid data leakage, all subsequent feature processing was performed only on the train set (800 reactions) and the same transformations then applied to the test and literature sets. Prior to fitting, features with no variance were removed using sklearn's VarianceThreshold feature selector, and features were standardised (the mean of each feature was subtracted from it before dividing by its standard deviation) using sklearn's StandardScaler to ensure they had zero mean and unit variance. Linear correlations between features were measured by Pearson's correlation coefficient (*r*) using SciPy,[10] revealing that many of the calculated features are highly correlated with each other. Although collinear features like this are not redundant and theoretically should not hinder the predictive power or generalisability of a model, they do introduce unnecessary complexity. Thus, where features exhibited collinearity above a threshold of 0.99 (Pearson's *r*), only features with the largest correlation with the DFT barrier were kept, and the rest removed. From the remaining features, 9 subsets were defined consisting of UFF MA features, AM1 MA features, AM1 TS features, AM1 MA and TS features, PM6 MA features, PM6 TS features, PM6 MA and TS features, DFT MA features, and AM1 and PM6 MA and TS features (Table S5). The free energy reaction barrier for each level of theory was included only in the combined MA and TS ("All") feature subsets. To further reduce the number of features in these All feature subsets, equivalent MA and TS features (for example, PBV (C_1) for the MA vs. PBV (C_1) for the TS) with correlations above a threshold of 0.84 (Pearson's *r*) were identified and only the feature with the largest correlation with the DFT barrier was kept. The choice of such lenient thresholds for Pearson's *r* was made to minimise the loss of information through the removal of collinear features and thus maximise prediction accuracy.

**Feature Notation**	**Feature Description**	**Source**
Distance	Bond forming distance (between C_1 and C_5) (TS feature only)	
Barrier	Quasiharmonic free energy reaction barrier (MA+TS feature only)	Goodvibes[7]
Energy	Electronic energy	Goodvibes[7]
Energy (SPE)	Electronic energy (with SPE correction)	Goodvibes[7]
Enthalpy	Enthalpy	Goodvibes[7]
QH Entropy	Quasiharmonic entropy	Goodvibes[7]
ZPE	Zero-point energy	Goodvibes[7]
QH GFE (SPE)	Quasiharmonic Gibbs free energy with SPE correction	Goodvibes[7]
HB Acceptors	Number of hydrogen bond acceptors	Pybel[37]
HB Donors	Number of hydrogen bond donors	Pybel[37]
Amide Bonds	Number of amide bonds	RDKit[38]
Chem. Pot.	Global chemical potential	HSAB[39]
Electrophilicity	Global electrophilicity	HSAB[39]
Hardness	Global hardness	HSAB[39]
Softness	Global softness	HSAB[39]
HOMO	Highest occupied molecular orbital (HOMO) energy	CCLIB[40]
LUMO	Lowest unoccupied molecular orbital (HOMO) energy	CCLIB[40]
Vib. Freq.	Lowest vibrational frequency (this is the imaginary frequency for TSs)	CCLIB[40]
Vib. IR	Lowest infrared intensity	CCLIB[40]
Mulliken (*n*)	Mulliken atomic charge (for each atom *n*)	CCLIB[40]
SASA (*n*)	Solvent accessible surface area (for each atom *n*)	Freesasa[41]
TPSA	Global topological polar surface area	RDKit[38,42]
Surface Area	Global surface area	Morfeus[43]
Surface Vol.	Global surface volume	Morfeus[43]
Pint (*n*)	Universal quantitative dispersion descriptor, P_{int} (for each atom *n*)	Morfeus[43,44]
PBV (*n*)	Percent buried volume (3.5Å radius) (for each atom *n*)	Morfeus[43,45,46]
Sterimol B1 (R_n)	Sterimol B_{min} parameter for R_1-R_4 substituents	Morfeus[43,47]
Sterimol B5 (R_n)	Sterimol B_{max} parameter for R_1-R_4 substituents	Morfeus[43,47]
Sterimol L (R_n)	Sterimol L parameter for R_1-R_4 substituents	Morfeus[43,47]
PEOE (*n*)	Partial equalisation of orbital electronegativities (for each atom *n*)	RDKit[38,48]
LogP (*n*)	Wildman-Crippen partition coefficient (for each atom *n*)	RDKit[38,49]
MR (*n*)	Wildman-Crippen molar refractivity (for each atom *n*)	RDKit[38,49]
EState (*n*)	Electrotopological state index (for each atom *n*)	RDKit[38,50,51]

Table S4. All extracted molecular and atomic features. All features were extracted for both the MA and TS and included in the respective feature subsets, except for the bond forming distance, which was only available for the TS, and the reaction barrier, which was only included in the relevant combined MA and TS feature subset. Atomic features denoted

with n (n = C_1, C_2, C_3, O_4, C_5, H_6, H_7, N_8, O_9, O_{10}, R_1, R_2, R_3, R_4) were extracted for all applicable atoms as per Fig. S7. Features highlighted in yellow were not available for UFF.

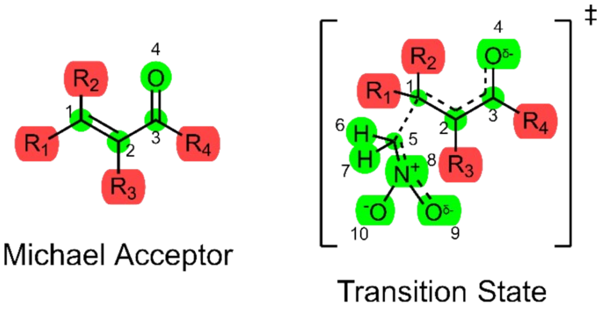

Fig. S7. Atomic properties were extracted for all applicable core atoms (highlighted green), as well as the first connected atom of each substituent (highlighted red).

Feature Subset	Full Feature Set
UFF MA	73
AM1 MA	87
AM1 TS	116
AM1 MA and TS (All)	138
PM6 MA	87
PM6 TS	116
PM6 MA and TS (All)	134
DFT MA	86
AM1 + PM6 MA and TS (All)	248

Table S5. Number of features in each feature subset.

3. Machine Learning

Each feature subset was trained on a variety of sklearn regression algorithms using the 80% train set to predict the DFT free energy reaction barrier. The algorithms used are summarised in Table S6; except for ridge regression, all algorithms were trained using their default sklearn parameters. For ridge regression, a large regularisation strength (α = 50) was used to account for the nature of the data; linear models, such as ridge regression, are parametric tests that are particularly prone to overfitting when large numbers of features are provided that exhibit some degree of multicollinearity.[52] Both the radial basis function (RBF) and polynomial kernels were employed for KRR and SVR, whilst GPR models were trained with the Matern kernel based on its previous success predicting reaction barriers.[53] Preliminary studies were also performed using elastic net regression, least absolute shrinkages and selection operator (LASSO) regression, KRR with a linear and sigmoid kernel, SVR with a linear and sigmoid kernel, GPR with a rational quadratic, RBF, and dot product kernel, and a multi-layer perceptron (MLP) neural network regressor, however these all either produced poor initial metrics or suffered from very large computational costs. To reduce the number of features in each model and prevent overfitting,[54] we employed feature selection algorithms within the train set prior to training each feature subset on each regressor. Sklearn's recursive feature elimination with 5-fold cross validation (RFECV) was used for regressors that are able to generate feature coefficients or feature importances, and mlxtend's sequential forward selection (SequentialFeatureSelector) with 5-fold cross validation (SFSCV) otherwise.[55] These algorithms calculate the 5-fold cross validation (CV) MAEs for each feature subset trained on the relevant regressor, allowing selection of the best combination of features for each subset. To avoid the use of these expensive algorithms with Gaussian process regression (GPR), each GPR model was instead fit initially with all features and then refit excluding any features with MAE-derived permutation feature importances below a specified threshold (a multiplier (0.5) of the mean importance) from the original model. Except for ridge regression (α = 50), feature selection was performed for all algorithms using their default sklearn parameters.

Regression Model	Feature Selection	Hyperparameter Tuning
Ridge regression (Ridge)[56]	RFECV	None
K-Nearest neighbour regression (NNR)[57]	SFSCV	Grid Search
Random forest regression (RFR)[58,59]	RFECV	Grid Search
Gradient boosting regression (GBR)[60]	RFECV	Grid Search
Support vector regression: RBF (SVR(RBF))[58,61]	SFSCV	Grid Search
Support vector regression: polynomial (SVR(poly))[58,61]	SFSCV	Grid Search
Kernel ridge regression: RBF (KRR(RBF))[62]	SFSCV	Grid Search
Kernel ridge regression: polynomial (KRR(poly))[62]	SFSCV	Grid Search
Gaussian process regression: Matern (GPR)[63]	Feature importances	Grid Search

Table S6. All regressors, feature selection methods, and hyperparameter tuning methods used for machine learning.

For each combination of regressor and optimised feature subset, hyperparameter tuning was performed within the train set using sklearn's GridSearchCV to search the hyperparameter space for the best CV MAE scores. Each regressor was subsequently refit using its optimised feature subset and optimised hyperparameters; the resulting models allow direct prediction of the DFT free energy reaction barrier. The grid of sklearn hyperparameters searched for each regressor are given below:

- **Ridge**: alpha = 50 (no hyperparameter tuning)
- **NNR**: n_neighbors = [1, 2, 3, 5, 10], weights = [uniform, distance], leaf_size = [1, 5, 10, 30, 50, 100], p = [1, 2]
- **RFR**: n_estimators = [10,50,100,250,500], max_depth = [1,5,10,50,100]
- **GBR**: n_estimators = [10,50,100,250,500], max_depth = [1,5,10,50,100]
- **SVR(RBF)**: C = [0.1, 0.5, 1, 2, 5, 10], epsilon = [0.1, 0.25, 0.5, 0.75, 1]
- **SVR(Poly)**: C = [0.1, 0.5, 1, 2, 5, 10], epsilon = [0.25, 0.5, 0.75, 1], coef0 = [1, 2, 3, 4, 5], degree = [1, 2, 3, 4, 5, 6, 7, 8, 9, 10]
- **KRR(RBF)**: alpha = [0.01, 0.1, 0.25, 0.5, 1, 2, 5, 10, 25, 50, 100], gamma = [None, 0.01, 0.05, 0.1, 0.5, 1]
- **KRR(Poly)**: alpha = [0.01, 0.1, 0.25, 0.5, 1, 2, 5, 10, 25, 50, 100], gamma = [0.01, 0.05, 0.1, 0.5, 1], coef0 = [1, 2, 3, 4, 5], degree = [1, 2, 3, 4, 5, 6, 7, 8, 9, 10].
- **GPR**: alpha = [1e-10, 1e-9, 1e-8, 1e-7, 1e-6, 1e-5, 1e-4, 1e-3, 0.01, 0.1, 1]

4. Model Analysis

To check for potential overfitting of the models, sklearn was used to perform 5-fold CV within the train set to generate MAE and coefficient of determination (R2) scores, as per their standard sklearn definitions, for each fitted model. To assess the individual model performances, external validation was performed using the 20% test set to calculate MAE (with standard errors) and R^2 scores between the observed and predicted DFT barriers for each fitted model. Standard errors were calculated by dividing the standard deviation of the individual absolute errors by the square root of the number of samples (200 for test set, and 35 or 37 for the literature sets). To further assess the generalisability of the models, external validation was performed using the two literature sets to calculate MAE scores (with standard errors), which are comparable between different sample sizes, between the observed and predicted DFT barriers for each fitted model. R^2 scores were not calculated for the literature sets due to their smaller size (n=35/37) and incomparability with the respective train (5-fold CV) and test scores (n=200).[64]

MAE-derived permutation feature importances (with standard errors) were calculated with sklearn over 10 repeats for each fitted model for both the train, test, and literature sets to identify which features were most important to the models. Learning curves were generated for each fitted model by training with increasingly smaller subsets of the train data (80 reactions were removed at each stage) and making predictions on the constant test set. The resulting train and test MAEs for each train set size were plotted to validate the size of the training set, identify potential overfitting, and indicate whether the equivalent metrics could be produced using less data.

5. All Machine Learning Metrics, Features, Hyperparameters

Metrics, features, and sklearn hyperparameters for all computed models are provided below. All train results are 5-fold CV. All MAEs (and standard errors) in kcal mol^{-1}. Except where specified, all models are derived from the original train-test splitting (random state = 0). For readability and comparative purposes, train, test, and literature (both sets) metrics for all computed models are also provided in Tables S7-10.

Model	AM1 MA	AM1 TS	AM1 All
Ridge	1.431 / 0.878 (87)	1.329 / 0.895 (105)	1.237 / 0.91 (98)
NNR	1.261 / 0.893 (16)	1.258 / 0.89 (54)	1.202 / 0.906 (44)
RFR	1.288 / 0.894 (49)	1.356 / 0.878 (25)	1.283 / 0.897 (26)
GBR	1.147 / 0.911 (40)	1.22 / 0.898 (51)	1.102 / 0.921 (58)
SVR(RBF)	0.956 / 0.935 (36)	0.916 / 0.94 (33)	0.907 / 0.939 (60)
SVR(poly)	1.027 / 0.931 (29)	1.011 / 0.931 (20)	0.967 / 0.938 (36)
KRR(RBF)	0.993 / 0.934 (35)	0.975 / 0.935 (38)	0.927 / 0.942 (42)
KRR(poly)	0.963 / 0.938 (21)	0.85 / 0.949 (42)	0.833 / 0.951 (48)
GPR	0.994 / 0.935 (72)	0.935 / 0.939 (87)	0.925 / 0.943 (101)
**Model**	**PM6 MA**	**PM6 TS**	**PM6 All**
Ridge	1.482 / 0.868 (67)	1.345 / 0.89 (67)	1.329 / 0.891 (126)
NNR	1.221 / 0.894 (41)	1.298 / 0.886 (21)	1.211 / 0.898 (31)
RFR	1.355 / 0.882 (28)	1.517 / 0.852 (32)	1.423 / 0.869 (26)
GBR	1.274 / 0.894 (61)	1.329 / 0.881 (55)	1.29 / 0.889 (56)
SVR(RBF)	0.968 / 0.929 (20)	0.922 / 0.937 (30)	0.936 / 0.934 (22)
SVR(poly)	0.993 / 0.932 (23)	1.09 / 0.921 (22)	1.038 / 0.924 (31)
KRR(RBF)	0.998 / 0.931 (42)	0.983 / 0.934 (30)	0.977 / 0.935 (40)
KRR(poly)	0.96 / 0.936 (31)	0.919 / 0.941 (47)	0.913 / 0.942 (40)
GPR	1.0 / 0.931 (78)	1.041 / 0.925 (85)	1.045 / 0.926 (105)
**Model**	**UFF MA**	**DFT MA**	**AM1 + PM6 All**
Ridge	1.557 / 0.855 (58)	1.45 / 0.868 (49)	1.166 / 0.918 (146)
NNR	1.235 / 0.899 (26)	1.239 / 0.897 (45)	1.127 / 0.905 (46)
RFR	1.282 / 0.895 (20)	1.298 / 0.894 (31)	1.301 / 0.892 (62)
GBR	1.168 / 0.909 (48)	1.161 / 0.913 (64)	1.12 / 0.916 (38)
SVR(RBF)	0.945 / 0.935 (30)	0.928 / 0.937 (31)	0.877 / 0.943 (58)
SVR(poly)	1.034 / 0.929 (22)	0.993 / 0.931 (23)	0.979 / 0.933 (51)
KRR(RBF)	0.975 / 0.933 (25)	0.961 / 0.936 (36)	0.915 / 0.944 (47)
KRR(poly)	0.943 / 0.941 (28)	0.918 / 0.942 (42)	0.815 / 0.955 (76)
GPR	0.986 / 0.932 (66)	0.957 / 0.939 (74)	0.955 / 0.938 (192)

Table S7. 5-fold CV train MAE / 5-fold CV train R^2 (number of features) for each regressor and feature subset.

Model	AM1 MA	AM1 TS	AM1 All
Ridge	1.531 ± 0.09 / 0.853 (87)	1.375 ± 0.08 / 0.882 (105)	1.255 ± 0.07 / 0.899 (98)
NNR	1.427 ± 0.09 / 0.862 (16)	1.438 ± 0.09 / 0.859 (54)	1.502 ± 0.08 / 0.861 (44)
RFR	1.361 ± 0.08 / 0.878 (49)	1.436 ± 0.08 / 0.872 (25)	1.363 ± 0.08 / 0.883 (26)
GBR	1.176 ± 0.08 / 0.903 (40)	1.284 ± 0.08 / 0.891 (51)	1.191 ± 0.07 / 0.904 (58)
SVR(RBF)	0.952 ± 0.08 / 0.922 (36)	0.927 ± 0.06 / 0.936 (33)	0.957 ± 0.07 / 0.93 (60)
SVR(poly)	1.117 ± 0.07 / 0.914 (29)	1.063 ± 0.07 / 0.919 (20)	0.953 ± 0.07 / 0.934 (36)
KRR(RBF)	1.002 ± 0.07 / 0.925 (35)	0.982 ± 0.07 / 0.931 (38)	0.987 ± 0.06 / 0.934 (42)
KRR(poly)	1.115 ± 0.07 / 0.914 (21)	0.988 ± 0.07 / 0.929 (42)	0.945 ± 0.06 / 0.935 (48)
GPR	1.018 ± 0.07 / 0.918 (72)	0.992 ± 0.08 / 0.918 (87)	0.964 ± 0.07 / 0.931 (101)
**Model**	**PM6 MA**	**PM6 TS**	**PM6 All**
Ridge	1.553 ± 0.09 / 0.849 (67)	1.484 ± 0.08 / 0.865 (67)	1.421 ± 0.08 / 0.872 (126)
NNR	1.361 ± 0.09 / 0.866 (41)	1.534 ± 0.1 / 0.835 (21)	1.398 ± 0.09 / 0.862 (31)
RFR	1.355 ± 0.08 / 0.881 (28)	1.608 ± 0.09 / 0.844 (32)	1.547 ± 0.08 / 0.857 (26)
GBR	1.267 ± 0.08 / 0.887 (61)	1.332 ± 0.08 / 0.887 (55)	1.253 ± 0.07 / 0.9 (56)
SVR(RBF)	1.021 ± 0.08 / 0.91 (20)	0.985 ± 0.07 / 0.926 (30)	1.033 ± 0.08 / 0.914 (22)
SVR(poly)	1.113 ± 0.08 / 0.907 (23)	1.066 ± 0.07 / 0.924 (22)	1.014 ± 0.07 / 0.926 (31)
KRR(RBF)	1.008 ± 0.07 / 0.922 (42)	1.007 ± 0.07 / 0.928 (30)	1.084 ± 0.07 / 0.92 (40)
KRR(poly)	1.042 ± 0.07 / 0.921 (31)	1.118 ± 0.07 / 0.92 (47)	1.159 ± 0.07 / 0.915 (40)
GPR	1.048 ± 0.08 / 0.912 (78)	1.017 ± 0.07 / 0.928 (85)	1.012 ± 0.07 / 0.927 (105)
**Model**	**UFF MA**	**DFT MA**	**AM1 + PM6 All**
Ridge	1.616 ± 0.09 / 0.837 (58)	1.561 ± 0.09 / 0.85 (49)	1.241 ± 0.07 / 0.902 (146)
NNR	1.361 ± 0.08 / 0.877 (26)	1.341 ± 0.09 / 0.869 (45)	1.346 ± 0.09 / 0.864 (46)
RFR	1.304 ± 0.09 / 0.878 (20)	1.296 ± 0.08 / 0.891 (31)	1.334 ± 0.08 / 0.888 (62)
GBR	1.146 ± 0.08 / 0.9 (48)	1.124 ± 0.07 / 0.91 (64)	1.201 ± 0.07 / 0.909 (38)
SVR(RBF)	0.975 ± 0.07 / 0.925 (30)	0.928 ± 0.07 / 0.927 (31)	0.976 ± 0.07 / 0.929 (58)
SVR(poly)	1.053 ± 0.07 / 0.922 (22)	1.035 ± 0.07 / 0.919 (23)	0.985 ± 0.07 / 0.93 (51)
KRR(RBF)	1.005 ± 0.07 / 0.921 (25)	1.044 ± 0.07 / 0.916 (36)	1.011 ± 0.06 / 0.932 (47)
KRR(poly)	1.057 ± 0.07 / 0.923 (28)	1.043 ± 0.07 / 0.921 (42)	0.883 ± 0.06 / 0.945 (76)
GPR	1.002 ± 0.08 / 0.918 (66)	1.013 ± 0.07 / 0.927 (74)	0.926 ± 0.06 / 0.937 (192)

Table S8. Test MAE ± standard error / test R^2 (number of features) for each regressor and feature subset.

Model	AM1 MA	AM1 TS	AM1 All
Ridge	1.653 ± 0.28 (87)	1.242 ± 0.21 (105)	1.412 ± 0.24 (98)
NNR	1.889 ± 0.27 (16)	1.484 ± 0.22 (54)	1.354 ± 0.28 (44)
RFR	1.261 ± 0.22 (49)	1.158 ± 0.24 (25)	1.343 ± 0.2 (26)
GBR	1.31 ± 0.2 (40)	1.33 ± 0.21 (51)	1.133 ± 0.17 (58)
SVR(RBF)	1.541 ± 0.24 (36)	1.424 ± 0.25 (33)	1.369 ± 0.25 (60)
SVR(poly)	1.805 ± 0.27 (29)	1.25 ± 0.18 (20)	1.539 ± 0.28 (36)
KRR(RBF)	1.638 ± 0.24 (35)	1.496 ± 0.23 (38)	1.109 ± 0.16 (42)
KRR(poly)	1.784 ± 0.27 (21)	1.236 ± 0.2 (42)	1.093 ± 0.16 (48)
GPR	1.389 ± 0.21 (72)	1.372 ± 0.22 (87)	1.118 ± 0.22 (101)
**Model**	**PM6 MA**	**PM6 TS**	**PM6 All**
Ridge	1.459 ± 0.25 (67)	1.186 ± 0.19 (67)	1.346 ± 0.24 (126)
NNR	1.456 ± 0.27 (41)	1.467 ± 0.28 (21)	1.253 ± 0.21 (31)
RFR	1.308 ± 0.22 (28)	1.08 ± 0.24 (32)	1.067 ± 0.24 (26)
GBR	1.473 ± 0.22 (61)	1.19 ± 0.22 (55)	1.153 ± 0.24 (56)
SVR(RBF)	1.872 ± 0.31 (20)	1.447 ± 0.24 (30)	1.484 ± 0.23 (22)
SVR(poly)	1.842 ± 0.25 (23)	1.857 ± 0.25 (22)	1.456 ± 0.26 (31)
KRR(RBF)	1.4 ± 0.23 (42)	1.316 ± 0.2 (30)	1.337 ± 0.18 (40)
KRR(poly)	1.732 ± 0.21 (31)	1.719 ± 0.22 (47)	1.745 ± 0.24 (40)
GPR	1.288 ± 0.21 (78)	1.402 ± 0.17 (85)	1.337 ± 0.19 (105)
**Model**	**UFF MA**	**DFT MA**	**AM1 + PM6 All**
Ridge	1.747 ± 0.21 (58)	1.611 ± 0.23 (49)	1.439 ± 0.25 (146)
NNR	1.692 ± 0.27 (26)	1.255 ± 0.2 (45)	1.497 ± 0.29 (46)
RFR	1.378 ± 0.21 (20)	1.162 ± 0.21 (31)	1.303 ± 0.19 (62)
GBR	1.326 ± 0.21 (48)	1.126 ± 0.21 (64)	1.154 ± 0.18 (38)
SVR(RBF)	1.557 ± 0.26 (30)	1.687 ± 0.23 (31)	1.289 ± 0.22 (58)
SVR(poly)	2.28 ± 0.31 (22)	2.511 ± 0.32 (23)	1.534 ± 0.24 (51)
KRR(RBF)	1.889 ± 0.29 (25)	1.691 ± 0.23 (36)	1.113 ± 0.16 (47)
KRR(poly)	2.414 ± 0.32 (28)	1.805 ± 0.2 (42)	1.461 ± 0.18 (76)
GPR	1.421 ± 0.23 (66)	1.464 ± 0.22 (74)	1.172 ± 0.2 (192)

Table S9. Literature (set 1) MAE ± standard error (number of features) for each regressor and feature subset.

Table S10. Literature (set 2) MAE ± standard error (number of features) for each regressor and feature subset.

Ridge, UFF MA (RFECV)

- Train MAE: 1.56
- Test MAE: 1.62 ± 0.09
- Lit. 1 MAE: 1.67 ± 0.21
- Lit. 2 MAE: 1.75 ± 0.21
- Lit. 3 MAE: 1.72 ± 0.2
- Train R^2: 0.86
- Test R^2: 0.84
- Lit. 1 R^2: -0.97
- Lit. 2 R^2: -0.19
- Lit. 3 R^2: -0.3

Hyperparameters: Ridge(alpha=50)

Features (58): EState (C1), EState (C2), EState (C3), EState (R1), EState (R3), Energy, HB Acceptors, HB Donors, LogP (C2), LogP (O4), LogP (R3), LogP (R4), MR (C1), MR (C2), MR (R3), MR (R4), Amide Bonds, PBV (C1), PBV (C2), PBV (C3), PBV (O4), PBV (R1), PBV (R2), PBV (R3), PBV (R4), PEOE (C1), PEOE (C2), PEOE (C3), PEOE (O4), PEOE (R1), PEOE (R2), PEOE (R4), Pint (C1), Pint (C2), Pint (C3), Pint (O4), Pint (R1), Pint (R2), Pint (R3), Pint (R4), Surface Area, SASA (C1), SASA (C2), SASA (C3), SASA (O4), SASA (R2), SASA (R3), SASA (R4), Sterimol B1 (R1), Sterimol L (R1), Sterimol B1 (R2), Sterimol B5 (R2), Sterimol L (R2), Sterimol B1 (R3), Sterimol L (R3), Sterimol B1 (R4), Sterimol L (R4), Vib. Freq.

Ridge, AM1 MA (RFECV)

- Train MAE: 1.43
- Test MAE: 1.53 ± 0.09
- Lit. 1 MAE: 1.46 ± 0.26
- Lit. 2 MAE: 1.65 ± 0.28
- Lit. 3 MAE: 1.59 ± 0.27
- Train R^2: 0.88
- Test R^2: 0.85
- Lit. 1 R^2: -1.06
- Lit. 2 R^2: -0.47
- Lit. 3 R^2: -0.5

Hyperparameters: Ridge(alpha=50)

Features (87): Chem. Pot., EState (C1), EState (C2), EState (C3), EState (O4), EState (R1), EState (R2), EState (R3), EState (R4), Electrophilicity, Energy, Enthalpy, QH Entropy, HB Acceptors, HB Donors, HOMO, Hardness, LUMO, LogP (O4), LogP (R1), LogP (R2), LogP (R3), LogP (R4), MR (C1), MR (C2), MR (R1), MR (R2), MR (R3), MR (R4), Mulliken (C1), Mulliken (C2), Mulliken (C3), Mulliken (O4), Mulliken (R1), Mulliken (R2), Mulliken (R3), Mulliken (R4), Amide Bonds, PBV (C1), PBV (C2), PBV (C3), PBV (O4), PBV (R1), PBV (R2), PBV (R3), PBV (R4), PEOE (C1), PEOE (C2), PEOE (C3), PEOE (O4), PEOE (R1), PEOE (R2), PEOE (R3), PEOE (R4), Pint (C1), Pint (C2), Pint (C3), Pint (O4), Pint (R1), Pint (R2), Pint (R3), Pint (R4), Surface Area, SASA (C1), SASA (C2), SASA (C3), SASA (O4), SASA (R1), SASA (R2), SASA (R3), SASA (R4), Sterimol B1 (R1), Sterimol B5 (R1), Sterimol L (R1), Sterimol B1 (R2), Sterimol B5 (R2), Sterimol L (R2), Sterimol B1 (R3), Sterimol B5 (R3), Sterimol L (R3), Sterimol B1 (R4), Sterimol B5 (R4), Sterimol L (R4), TPSA, Vib. Freq., Vib. IR, ZPE

Ridge, AM1 TS (RFECV)

- Train MAE: 1.33
- Test MAE: 1.38 ± 0.08
- Lit. 1 MAE: 1.18 ± 0.22
- Lit. 2 MAE: 1.24 ± 0.21
- Lit. 3 MAE: 1.13 ± 0.21
- Train R^2: 0.9
- Test R^2: 0.88
- Lit. 1 R^2: -0.39
- Lit. 2 R^2: 0.17
- Lit. 3 R^2: 0.15

Hyperparameters: Ridge(alpha=50)

Features (105): Chem. Pot., Distance, EState (C1), EState (C2), EState (C3), EState (N8), EState (O4), EState (R3), Electrophilicity, Energy (SPE), Enthalpy, QH Entropy, HB Acceptors, HB Donors, HOMO, Hardness, LUMO, LogP (C1), LogP (C2), LogP (O4), LogP (R1), LogP (R2), LogP (R3), LogP (R4), MR (C1), MR (C2), MR (R1), MR (R2), MR (R3), MR (R4), Mulliken (C1), Mulliken (C2), Mulliken (C3), Mulliken (C5), Mulliken (H6), Mulliken (H7), Mulliken (N8), Mulliken (O10), Mulliken (O4), Mulliken (O9), Mulliken (R2), Mulliken (R3), Mulliken (R4), Amide Bonds, PBV

(C1), PBV (C2), PBV (C3), PBV (C5), PBV (H6), PBV (H7), PBV (N8), PBV (O4), PBV (R2), PBV (R3), PBV (R4), PEOE (C1), PEOE (C2), PEOE (C3), PEOE (O4), PEOE (R1), PEOE (R2), PEOE (R3), PEOE (R4), Pint (C1), Pint (C2), Pint (C3), Pint (C5), Pint (H6), Pint (H7), Pint (N8), Pint (O10), Pint (O4), Pint (O9), Pint (R1), Pint (R2), Pint (R3), Pint (R4), Surface Vol., SASA (C1), SASA (C2), SASA (C3), SASA (C5), SASA (H6), SASA (N8), SASA (O10), SASA (O4), SASA (O9), SASA (R1), SASA (R2), SASA (R3), SASA (R4), Sterimol B1 (R1), Sterimol B5 (R1), Sterimol B1 (R2), Sterimol B5 (R2), Sterimol L (R2), Sterimol B1 (R3), Sterimol B5 (R3), Sterimol L (R3), Sterimol B5 (R4), Sterimol L (R4), TPSA, Vib. Freq., Vib. IR, ZPE

Ridge, AM1 All (RFECV)

- Train MAE: 1.24
- Test MAE: 1.25 ± 0.07
- Lit. 1 MAE: 1.31 ± 0.25
- Lit. 2 MAE: 1.41 ± 0.24
- Lit. 3 MAE: 1.36 ± 0.24
- Train R^2: 0.91
- Test R^2: 0.9
- Lit. 1 R^2: -0.73
- Lit. 2 R^2: -0.08
- Lit. 3 R^2: -0.13

Hyperparameters: Ridge(alpha=50)

Features (98): Barrier, MA Chem. Pot., MA EState (C1), MA Electrophilicity, MA Energy, MA QH Entropy, MA HOMO, MA Hardness, MA MR (C2), MA Mulliken (C1), MA Mulliken (O4), MA Mulliken (R3), MA PBV (R4), MA Pint (C1), MA Pint (C3), MA Pint (R1), MA Pint (R4), MA SASA (C1), MA SASA (C3), MA SASA (R1), MA SASA (R4), MA Sterimol B1 (R1), MA Sterimol L (R1), MA Sterimol L (R2), MA Sterimol B5 (R3), MA Sterimol B1 (R4), MA Sterimol L (R4), MA Vib. Freq., MA Vib. IR, TS Chem. Pot., TS Distance, TS EState (C1), TS EState (C2), TS EState (C3), TS EState (N8), TS EState (O4), TS EState (R1), TS EState (R2), TS EState (R3), TS Electrophilicity, TS Energy (SPE), TS Enthalpy, TS HB Acceptors, TS HB Donors, TS HOMO, TS Hardness, TS LUMO, TS LogP (C1), TS LogP (R2), TS LogP (R3), TS LogP (R4), TS MR (R3), TS MR (R4), TS Mulliken (C1), TS Mulliken (C2), TS Mulliken (C3), TS Mulliken (C5), TS Mulliken (H6), TS Mulliken (H7), TS Mulliken (N8), TS Mulliken (O10), TS Mulliken (O4), TS Mulliken (R3), TS Mulliken (R4), TS Amide Bonds, TS PBV (C1), TS PBV (C3), TS PBV (C5), TS PBV (H6), TS PBV (H7), TS PBV (N8), TS PBV (O9), TS PBV (R3), TS PEOE (C1), TS PEOE (C2), TS PEOE (R2), TS PEOE (R4), TS Pint (C5), TS Pint (H6), TS Pint (H7), TS Pint (O10), TS Pint (O4), TS Pint (O9), TS SASA (C5), TS SASA (N8), TS SASA (O10), TS SASA (O4), TS SASA (O9), TS SASA (R3), TS Sterimol B1 (R1), TS Sterimol B5 (R2), TS Sterimol B1 (R3), TS Sterimol B5 (R3), TS Sterimol L (R3), TS Sterimol B5 (R4), TS Vib. Freq., TS Vib. IR, TS ZPE

Ridge, PM6 MA (RFECV)

- Train MAE: 1.48
- Test MAE: 1.55 ± 0.09
- Lit. 1 MAE: 1.27 ± 0.22
- Lit. 2 MAE: 1.46 ± 0.25
- Lit. 3 MAE: 1.4 ± 0.23
- Train R^2: 0.87
- Test R^2: 0.85
- Lit. 1 R^2: -0.52
- Lit. 2 R^2: -0.14
- Lit. 3 R^2: -0.13

Hyperparameters: Ridge(alpha=50)

Features (67): EState (C1), EState (C2), EState (C3), EState (R2), EState (R3), EState (R4), HB Acceptors, HB Donors, HOMO, LUMO, LogP (O4), LogP (R2), LogP (R4), MR (C1), MR (C2), MR (R2), MR (R3), MR (R4), Mulliken (C3), Mulliken (O4), Mulliken (R2), Mulliken (R3), Mulliken (R4), Amide Bonds, PBV (C1), PBV (C2), PBV (C3), PBV (R2), PBV (R3), PBV (R4), PEOE (C1), PEOE (C3), PEOE (O4), PEOE (R1), PEOE (R2), PEOE (R3), PEOE (R4), Pint (C1), Pint (C2), Pint (C3), Pint (O4), Pint (R1), Pint (R2), Pint (R3), Pint (R4), Surface Area, SASA (C1), SASA (C2), SASA (C3), SASA (O4), SASA (R2), SASA (R3), SASA (R4), Softness, Sterimol B1 (R1), Sterimol B5 (R1), Sterimol B1 (R2), Sterimol B5 (R2), Sterimol L (R2), Sterimol B1 (R3), Sterimol B5 (R3), Sterimol L (R3), Sterimol B1 (R4), Sterimol L (R4), TPSA, Vib. Freq., Vib. IR

Ridge, PM6 TS (RFECV)

- Train MAE: 1.34
- Test MAE: 1.48 ± 0.08
- Lit. 1 MAE: 1.14 ± 0.19
- Lit. 2 MAE: 1.19 ± 0.19
- Lit. 3 MAE: 1.14 ± 0.18
- Train R^2: 0.89
- Test R^2: 0.86
- Lit. 1 R^2: -0.17
- Lit. 2 R^2: 0.31
- Lit. 3 R^2: 0.26

Hyperparameters: Ridge(alpha=50)

Features (67): Chem. Pot., EState (C2), EState (C3), EState (O4), EState (R3), EState (R4), Electrophilicity, Energy, Enthalpy, HB Acceptors, HB Donors, HOMO, LUMO, LogP (O4), LogP (R2), LogP (R3), LogP (R4), MR (C1), MR (R2), MR (R4), Mulliken (C1), Mulliken (C2), Mulliken (C3), Mulliken (C5), Mulliken (H6), Mulliken (O4), Mulliken (R3), Mulliken (R4), Amide Bonds, PBV (C1), PBV (C3), PBV (C5), PBV (H6), PBV (N8), PBV (O10), PBV (R3), PBV (R4), PEOE (C1), PEOE (C3), PEOE (R1), PEOE (R2), PEOE (R4), Pint (C1), Pint (C2), Pint (C3), Pint (C5), Pint (H7), Pint (R3), Pint (R4), SASA (C1), SASA (C5), SASA (O10), SASA (O4), SASA (O9), SASA (R1), SASA (R2), SASA (R3), SASA (R4), Softness, Sterimol B1 (R1), Sterimol L (R1), Sterimol B1 (R2), Sterimol B5 (R2), Sterimol L (R3), Sterimol L (R4), Vib. Freq., Vib. IR

Ridge, PM6 All (RFECV)

- Train MAE: 1.33
- Test MAE: 1.42 ± 0.08
- Lit. 1 MAE: 1.22 ± 0.24
- Lit. 2 MAE: 1.35 ± 0.24
- Lit. 3 MAE: 1.24 ± 0.23
- Train R^2: 0.89
- Test R^2: 0.87
- Lit. 1 R^2: -0.58
- Lit. 2 R^2: -0.04
- Lit. 3 R^2: -0.0

Hyperparameters: Ridge(alpha=50)

Features (126): Barrier, MA Chem. Pot., MA EState (C1), MA Electrophilicity, MA QH Entropy, MA HOMO, MA LUMO, MA MR (C1), MA MR (C2), MA Mulliken (C2), MA Mulliken (O4), MA Mulliken (R1), MA Mulliken (R2), MA Mulliken (R3), MA PBV (C1), MA PBV (O4), MA PBV (R1), MA PBV (R2), MA PEOE (C2), MA PEOE (R3), MA Pint (C1), MA Pint (C2), MA Pint (C3), MA Pint (R1), MA Pint (R2), MA Pint (R3), MA Pint (R4), MA SASA (C1), MA SASA (C3), MA SASA (O4), MA SASA (R1), MA SASA (R2), MA SASA (R4), MA Softness, MA Sterimol B1 (R1), MA Sterimol B5 (R1), MA Sterimol B1 (R2), MA Sterimol L (R2), MA Sterimol B1 (R3), MA Sterimol B5 (R3), MA Sterimol B1 (R4), MA Sterimol B5 (R4), MA Sterimol L (R4), MA TPSA, MA Vib. Freq., MA Vib. IR, TS Chem. Pot., TS Distance, TS EState (C2), TS EState (C3), TS EState (H7), TS EState (N8), TS EState (O4), TS EState (R1), TS EState (R2), TS EState (R3), TS EState (R4), TS Electrophilicity, TS Energy, TS Enthalpy, TS HB Acceptors, TS HB Donors, TS HOMO, TS LUMO, TS LogP (C2), TS LogP (O4), TS LogP (R1), TS LogP (R2), TS LogP

(R3), TS LogP (R4), TS MR (C1), TS MR (R1), TS MR (R2), TS MR (R3), TS MR (R4), TS Mulliken (C1), TS Mulliken (C2), TS Mulliken (C3), TS Mulliken (C5), TS Mulliken (H6), TS Mulliken (H7), TS Mulliken (N8), TS Mulliken (O10), TS Mulliken (O4), TS Mulliken (O9), TS Mulliken (R4), TS Amide Bonds, TS PBV (C2), TS PBV (C3), TS PBV (C5), TS PBV (H6), TS PBV (H7), TS PBV (N8), TS PBV (O10), TS PBV (O9), TS PBV (R3), TS PBV (R4), TS PEOE (C1), TS PEOE (R1), TS PEOE (R2), TS PEOE (R4), TS Pint (C5), TS Pint (H6), TS Pint (H7), TS Pint (N8), TS Pint (O10), TS Pint (O4), TS Pint (O9), TS SASA (C2), TS SASA (C5), TS SASA (H6), TS SASA (H7), TS SASA (N8), TS SASA (O10), TS SASA (O4), TS SASA (O9), TS SASA (R3), TS Softness, TS Sterimol B1 (R1), TS Sterimol B5 (R2), TS Sterimol B1 (R3), TS Sterimol L (R3), TS Sterimol B5 (R4), TS Vib. Freq., TS Vib. IR, TS ZPE

Ridge, DFT MA (RFECV)

- Train MAE: 1.45
- Test MAE: 1.56 ± 0.09
- Lit. 1 MAE: 1.43 ± 0.2
- Lit. 2 MAE: 1.61 ± 0.23
- Lit. 3 MAE: 1.56 ± 0.21
- Train R^2: 0.87
- Test R^2: 0.85
- Lit. 1 R^2: -0.56
- Lit. 2 R^2: -0.17
- Lit. 3 R^2: -0.2

Hyperparameters: Ridge(alpha=50)

Features (49): EState (C2), EState (C3), EState (R3), Enthalpy, HB Acceptors, HB Donors, HOMO, Hardness, LUMO, LogP (O4), LogP (R4), MR (C1), MR (C2), MR (R3), MR (R4), Mulliken (C3), Mulliken (O4), Mulliken (R3), Mulliken (R4), Amide Bonds, PBV (C1), PBV (C2), PBV (C3), PBV (R1), PBV (R3), PEOE (C1), PEOE (C2), PEOE (C3), Pint (C1), Pint (C3), Pint (O4), Pint (R1), Pint (R2), Pint (R3), Pint (R4), SASA (C1), SASA (C2), SASA (C3), SASA (R3), SASA (R4), Sterimol B1 (R1), Sterimol B5 (R1), Sterimol B5 (R2), Sterimol L (R2), Sterimol B1 (R3), Sterimol B5 (R3), Sterimol L (R3), Sterimol B1 (R4), Sterimol L (R4)

Ridge, AM1 PM6 All (RFECV)

- Train MAE: 1.17
- Test MAE: 1.24 ± 0.07
- Lit. 1 MAE: 1.32 ± 0.24
- Lit. 2 MAE: 1.44 ± 0.25
- Lit. 3 MAE: 1.35 ± 0.23
- Train R^2: 0.92
- Test R^2: 0.9
- Lit. 1 R^2: -0.73
- Lit. 2 R^2: -0.11
- Lit. 3 R^2: -0.12

Hyperparameters: Ridge(alpha=50)

Features (146): AM1 Barrier, AM1 MA Chem. Pot., AM1 MA Electrophilicity, AM1 MA Energy, AM1 MA HOMO, AM1 MA Hardness, AM1 MA Mulliken (C1), AM1 MA Mulliken (O4), AM1 MA Mulliken (R3), AM1 MA PBV (R4), AM1 MA Pint (C1), AM1 MA Pint (C2), AM1 MA Pint (C3), AM1 MA Pint (R1), AM1 MA Pint (R4), AM1 MA SASA (C1), AM1 MA SASA (C3), AM1 MA Sterimol B1 (R1), AM1 MA Sterimol B5 (R1), AM1 MA Sterimol L (R2), AM1 MA Sterimol B1 (R4), AM1 MA Sterimol B5 (R4), AM1 MA Vib. Freq., AM1 MA Vib. IR, AM1 TS Chem. Pot., AM1 TS Distance, AM1 TS EState (C2), AM1 TS EState (C3), AM1 TS EState (N8), AM1 TS Electrophilicity, AM1 TS Energy (SPE), AM1 TS Enthalpy, AM1 TS HOMO, AM1 TS Hardness, AM1 TS LogP (C1), AM1 TS Mulliken (C1), AM1 TS Mulliken (C2), AM1 TS Mulliken (C3), AM1 TS Mulliken (C5), AM1 TS Mulliken (H6), AM1 TS Mulliken (H7), AM1 TS Mulliken (N8), AM1 TS Mulliken (R3), AM1 TS Mulliken (R4), AM1 TS PBV (C1), AM1 TS PBV (C2), AM1 TS PBV (C5), AM1 TS PBV (H6), AM1 TS PBV (H7), AM1 TS PBV (N8), AM1 TS PBV (O9), AM1 TS PBV (R3), AM1 TS PEOE (C1), AM1 TS PEOE (R2), AM1 TS PEOE (R4), AM1 TS Pint (C5), AM1 TS Pint (O10), AM1 TS Pint (O4), AM1 TS Pint (O9), AM1 TS SASA (C5), AM1 TS SASA (N8), AM1 TS SASA (O10), AM1 TS SASA (O4), AM1 TS SASA (O9), AM1 TS SASA (R2), AM1 TS Sterimol B1 (R1), AM1 TS Sterimol B5 (R2), AM1 TS Sterimol B1 (R3), AM1 TS Sterimol B5 (R3), AM1 TS Sterimol L (R3), PM6 Barrier, PM6 MA EState (C1), PM6 MA Mulliken (O4), PM6 MA Mulliken (R2), PM6 MA PBV (C1), PM6 MA PBV (R2), PM6 MA Pint (C1), PM6 MA Pint (C2), PM6 MA Pint (C3), PM6 MA Pint (R2), PM6 MA Pint (R3), PM6 MA Pint (R4), PM6 MA SASA (C1), PM6 MA SASA (C3), PM6 MA SASA (O4), PM6 MA SASA (R1), PM6 MA SASA (R4), PM6 MA Sterimol B5 (R1), PM6 MA Sterimol L (R2), PM6 MA Sterimol B1 (R3), PM6 MA Vib. Freq., PM6 TS Chem. Pot., PM6 TS Distance, PM6 TS EState (C2), PM6 TS EState (C3), PM6 TS EState (H7), PM6 TS EState (R1), PM6 TS EState (R3), PM6 TS Electrophilicity, PM6 TS Energy, PM6 TS HB Acceptors, PM6 TS HB Donors, PM6 TS HOMO, PM6 TS LUMO, PM6 TS LogP (R2), PM6 TS LogP (R3), PM6 TS LogP (R4), PM6 TS MR (C1), PM6 TS MR (R3), PM6 TS MR (R4), PM6 TS Mulliken (C1), PM6 TS Mulliken (C2), PM6 TS Mulliken (C5), PM6 TS Mulliken (H6), PM6 TS Mulliken (N8), PM6 TS Mulliken (O4), PM6 TS Mulliken (R4), PM6 TS Amide Bonds, PM6 TS PBV (C3), PM6 TS PBV (C5), PM6 TS PBV (H6), PM6 TS PBV (N8), PM6 TS PBV (O10), PM6 TS PBV (O9), PM6 TS PBV (R1), PM6 TS PBV (R3), PM6 TS PEOE (C1), PM6 TS PEOE (R2), PM6 TS Pint (C5), PM6 TS Pint (H6), PM6 TS Pint (H7), PM6 TS Pint (O10), PM6 TS SASA (C2), PM6 TS SASA (C5), PM6 TS SASA (H6), PM6 TS SASA (O10), PM6 TS SASA (O4), PM6 TS SASA (R3), PM6 TS Sterimol B1 (R1), PM6 TS Sterimol B5 (R2), PM6 TS Sterimol B1 (R3), PM6 TS Sterimol L (R3), PM6 TS Sterimol B1 (R4), PM6 TS Sterimol B5 (R4), PM6 TS Vib. Freq., PM6 TS Vib. IR

NNR, UFF MA (SFS)

- Train MAE: 1.24
- Test MAE: 1.36 ± 0.08
- Lit. 1 MAE: 1.37 ± 0.16
- Lit. 2 MAE: 1.69 ± 0.27
- Lit. 3 MAE: 1.65 ± 0.24
- Train R^2: 0.9
- Test R^2: 0.88
- Lit. 1 R^2: -0.27
- Lit. 2 R^2: -0.42
- Lit. 3 R^2: -0.43

Hyperparameters: KNeighborsRegressor(leaf_size=1, p=1, weights='distance')

Features (26): EState (C2), EState (C3), QH Entropy, HB Acceptors, LogP (R3), LogP (R4), MR (C2), MR (R4), Amide Bonds, PBV (C1), PBV (C2), PBV (C3), PBV (R3), PEOE (R4), Pint (C1), Pint (C2), Pint (C3), Pint (R3), SASA (C1), SASA (C2), SASA (R3), Sterimol B1 (R1), Sterimol B5 (R2), Sterimol B5 (R3), Sterimol B1 (R4), ZPE

NNR, AM1 MA (SFS)

- Train MAE: 1.26
- Test MAE: 1.43 ± 0.09
- Lit. 1 MAE: 1.58 ± 0.18
- Lit. 2 MAE: 1.89 ± 0.27
- Lit. 3 MAE: 1.84 ± 0.24
- Train R^2: 0.89
- Test R^2: 0.86
- Lit. 1 R^2: -0.61
- Lit. 2 R^2: -0.62
- Lit. 3 R^2: -0.62

Hyperparameters: KNeighborsRegressor(leaf_size=1, weights='distance')

Features (16): EState (C2), EState (C3), EState (O4), LogP (O4), LogP (R4), MR (R4), Mulliken (R3), Mulliken (R4), Amide Bonds, PBV (C1), PBV (C3), Pint (C1), Pint (R4), SASA (C2), Sterimol B1 (R1), ZPE

NNR, AM1 TS (SFS)

- Train MAE: 1.26
- Test MAE: 1.44 ± 0.09
- Lit. 1 MAE: 1.26 ± 0.17
- Lit. 2 MAE: 1.48 ± 0.22
- Lit. 3 MAE: 1.46 ± 0.21
- Train R^2: 0.89
- Test R^2: 0.86
- Lit. 1 R^2: -0.16
- Lit. 2 R^2: -0.03
- Lit. 3 R^2: -0.1

Hyperparameters: KNeighborsRegressor(leaf_size=1, weights='distance')

Features (54): Chem. Pot., Distance, EState (C2), EState (C3), EState (R2), EState (R3), EState (R4), Enthalpy, HB Acceptors, HB Donors, HOMO, Hardness, LUMO, LogP (O4), LogP (R4), MR (R4), Mulliken (C1), Mulliken (C3), Mulliken (C5), Mulliken (O4), Mulliken (O9), Mulliken (R3), Mulliken (R4), Amide Bonds, PBV (C1), PBV (C2), PBV (C3), PBV (C5), PBV (H6), PBV (H7), PBV (R4), PEOE (C1), PEOE (C3), PEOE (O4), PEOE (R2), PEOE (R4), Pint (C1), Pint (C2), Pint (C3), Pint (O4), Pint (R4), SASA (C1), SASA (C3), SASA (C5), SASA (H6), SASA (N8), SASA (R3), Sterimol B1 (R1), Sterimol B1 (R2), Sterimol B5 (R2), Sterimol L (R4), TPSA, Vib. Freq., ZPE

NNR, AM1 All (SFS)

- Train MAE: 1.2
- Test MAE: 1.5 ± 0.08
- Lit. 1 MAE: 1.02 ± 0.17
- Lit. 2 MAE: 1.35 ± 0.28
- Lit. 3 MAE: 1.26 ± 0.23
- Train R^2: 0.91
- Test R^2: 0.86
- Lit. 1 R^2: 0.06
- Lit. 2 R^2: -0.21
- Lit. 3 R^2: -0.03

Hyperparameters: KNeighborsRegressor(leaf_size=1, weights='distance')

Features (44): Barrier, MA Electrophilicity, MA Mulliken (C1), MA Mulliken (O4), MA Mulliken (R3), MA PBV (R4), MA PEOE (C3), MA PEOE (O4), MA Pint (C2), MA Pint (R4), MA SASA (C1), MA SASA (C3), MA Sterimol B1 (R2), MA Sterimol B1 (R3), MA TPSA, MA Vib. IR, TS Chem. Pot., TS EState (C2), TS EState (R4), TS HB Acceptors, TS HB Donors, TS HOMO, TS LogP (O4), TS LogP (R4), TS MR (R2), TS MR (R3), TS MR (R4), TS Mulliken (C1), TS Mulliken (C3), TS Mulliken (O4), TS Mulliken (R4), TS Amide Bonds, TS PBV (C1), TS PBV (C2), TS PBV (C3), TS PBV (C5), TS PBV (H7), TS PEOE (R4), TS Pint (O4), TS SASA (C2), TS SASA (C5), TS SASA (N8), TS Sterimol B1 (R1), TS Sterimol B5 (R2)

NNR, PM6 MA (SFS)

- Train MAE: 1.22
- Test MAE: 1.36 ± 0.09
- Lit. 1 MAE: 1.13 ± 0.15
- Lit. 2 MAE: 1.46 ± 0.27
- Lit. 3 MAE: 1.41 ± 0.24
- Train R^2: 0.89
- Test R^2: 0.87
- Lit. 1 R^2: 0.07
- Lit. 2 R^2: -0.22
- Lit. 3 R^2: -0.21

Hyperparameters: KNeighborsRegressor(leaf_size=1, n_neighbors=3, weights='distance')

Features (41): EState (C2), EState (C3), EState (R4), Energy, QH Entropy, HB Acceptors, HB Donors, LogP (O4), LogP (R4), MR (R2), MR (R4), Mulliken (C1), Mulliken (C2), Mulliken (C3), Mulliken (R3), Mulliken (R4), Amide Bonds, PBV (C1), PBV (C2), PBV (C3), PEOE (C2), PEOE (C3), PEOE (O4), PEOE (R2), PEOE (R3), PEOE (R4), Pint (C1), Pint (C2), Pint (C3), Pint (O4), Pint (R4), Surface Area, SASA (C1), SASA (C2), SASA (R1), Sterimol B1 (R2), Sterimol B5 (R2), Sterimol B1 (R3), Sterimol L (R4), TPSA, ZPE

NNR, PM6 TS (SFS)

- Train MAE: 1.3
- Test MAE: 1.53 ± 0.1
- Lit. 1 MAE: 1.15 ± 0.18
- Lit. 2 MAE: 1.47 ± 0.28
- Lit. 3 MAE: 1.28 ± 0.19
- Train R^2: 0.89
- Test R^2: 0.84
- Lit. 1 R^2: -0.11
- Lit. 2 R^2: -0.29
- Lit. 3 R^2: 0.11

Hyperparameters: KNeighborsRegressor(leaf_size=1, n_neighbors=3, p=1, weights='distance')

Features (21): EState (C3), EState (R4), HB Acceptors, HB Donors, LogP (O4), MR (R4), Mulliken (C3), Mulliken (R3), Mulliken (R4), Amide Bonds, PBV (C1), PBV (C2), PBV (C5), PEOE (C3), PEOE (O4), PEOE (R4), Pint (C1), Pint (R3), SASA (C1), Sterimol L (R4), ZPE

NNR, PM6 All (SFS)

- Train MAE: 1.21
- Test MAE: 1.4 ± 0.09
- Lit. 1 MAE: 1.04 ± 0.16
- Lit. 2 MAE: 1.25 ± 0.21
- Lit. 3 MAE: 1.18 ± 0.18
- Train R^2: 0.9
- Test R^2: 0.86
- Lit. 1 R^2: 0.09
- Lit. 2 R^2: 0.16
- Lit. 3 R^2: 0.24

Hyperparameters: KNeighborsRegressor(leaf_size=1, weights='distance')

Features (31): MA Mulliken (R3), MA PBV (C1), MA PEOE (C2), MA PEOE (C3), MA PEOE (O4), MA Pint (C1), MA Pint (C2), MA Pint (R1), MA Pint (R4), MA SASA (C1), MA SASA (C3), MA Sterimol B1 (R1), MA Sterimol B1 (R3), TS EState (C2), TS EState (R4), TS HB Acceptors, TS HB Donors, TS HOMO, TS LogP (O4), TS MR (R4), TS Mulliken (C1), TS Mulliken (C3), TS Mulliken (O4), TS Mulliken (R4), TS Amide Bonds, TS PBV (C2), TS PBV (C3), TS PBV (C5), TS PEOE (R4), TS SASA (C5), TS ZPE

NNR, DFT MA (SFS)

- Train MAE: 1.24
- Test MAE: 1.34 ± 0.09
- Lit. 1 MAE: 1.02 ± 0.13
- Lit. 2 MAE: 1.25 ± 0.2
- Lit. 3 MAE: 1.18 ± 0.16
- Train R^2: 0.9

- Test R^2: 0.87
- Lit. 1 R^2: 0.27
- Lit. 2 R^2: 0.21
- Lit. 3 R^2: 0.31

Hyperparameters: KNeighborsRegressor(leaf_size=1, weights='distance')

Features (45): Chem. Pot., EState (C2), EState (C3), EState (O4), EState (R1), EState (R2), EState (R3), EState (R4), QH Entropy, HB Acceptors, HB Donors, LogP (O4), LogP (R4), MR (R4), Mulliken (C1), Mulliken (C2), Mulliken (C3), Mulliken (O4), Mulliken (R3), Mulliken (R4), Amide Bonds, PBV (C1), PBV (C2), PBV (C3), PBV (O4), PBV (R4), PEOE (C3), PEOE (O4), PEOE (R3), PEOE (R4), Pint (C1), Pint (C2), Pint (O4), Pint (R4), Surface Area, SASA (C1), SASA (C3), SASA (O4), SASA (R4), Sterimol B1 (R1), Sterimol B1 (R4), Sterimol L (R4), TPSA, Vib. IR, ZPE

NNR, AM1 PM6 All (SFS)

- Train MAE: 1.13
- Test MAE: 1.35 ± 0.09
- Lit. 1 MAE: 1.14 ± 0.16
- Lit. 2 MAE: 1.5 ± 0.29
- Lit. 3 MAE: 1.29 ± 0.18
- Train R^2: 0.9
- Test R^2: 0.86
- Lit. 1 R^2: 0.01
- Lit. 2 R^2: -0.35
- Lit. 3 R^2: 0.16

Hyperparameters: KNeighborsRegressor(leaf_size=1, n_neighbors=3, weights='distance')

Features (46): AM1 Barrier, AM1 MA HOMO, AM1 MA Mulliken (O4), AM1 MA PBV (R4), AM1 MA SASA (C1), AM1 MA Sterimol B1 (R1), AM1 MA Sterimol B1 (R2), AM1 MA Sterimol L (R4), AM1 MA Vib. Freq., AM1 MA Vib. IR, AM1 TS EState (C2), AM1 TS EState (C3), AM1 TS LUMO, AM1 TS Mulliken (C3), AM1 TS Mulliken (O4), AM1 TS PBV (C1), AM1 TS PBV (C5), AM1 TS PEOE (C3), AM1 TS PEOE (O4), AM1 TS PEOE (R4), AM1 TS Sterimol B5 (R2), PM6 MA QH Entropy, PM6 MA HOMO, PM6 MA Mulliken (C2), PM6 MA Mulliken (O4), PM6 MA Mulliken (R3), PM6 MA PBV (C1), PM6 MA PBV (O4), PM6 MA PEOE (C3), PM6 MA PEOE (O4), PM6 MA PEOE (R3), PM6 MA Pint (C1), PM6 MA Sterimol L (R4), PM6 TS EState (C3), PM6 TS EState (H7), PM6 TS HB Acceptors, PM6 TS HB Donors, PM6 TS HOMO, PM6 TS LogP (O4), PM6 TS LogP (R4), PM6 TS MR (R4), PM6 TS Amide Bonds, PM6 TS PBV (C5), PM6 TS PEOE (C1), PM6 TS PEOE (R2), PM6 TS SASA (C5)

RFR, UFF MA (RFECV)

- Train MAE: 1.28
- Test MAE: 1.3 ± 0.09
- Lit. 1 MAE: 1.12 ± 0.12
- Lit. 2 MAE: 1.38 ± 0.21
- Lit. 3 MAE: 1.29 ± 0.18
- Train R^2: 0.9
- Test R^2: 0.88
- Lit. 1 R^2: 0.19
- Lit. 2 R^2: 0.08
- Lit. 3 R^2: 0.18

Hyperparameters: RandomForestRegressor(max_depth=100, n_estimators=500)

Features (20): EState (C2), EState (C3), EState (R3), EState (R4), LogP (R4), PBV (C1), PBV (C3), PBV (R1), PBV (R3), PEOE (C3), PEOE (O4), PEOE (R4), SASA (C1), SASA (C2), SASA (C3), SASA (R4), Sterimol L (R2), Sterimol B5 (R3), Sterimol L (R3), Sterimol B1 (R4)

RFR, AM1 MA (RFECV)

- Train MAE: 1.29
- Test MAE: 1.36 ± 0.08
- Lit. 1 MAE: 0.98 ± 0.12
- Lit. 2 MAE: 1.26 ± 0.22
- Lit. 3 MAE: 1.2 ± 0.2
- Train R^2: 0.89
- Test R^2: 0.88
- Lit. 1 R^2: 0.34
- Lit. 2 R^2: 0.12
- Lit. 3 R^2: 0.17

Hyperparameters: RandomForestRegressor(max_depth=100, n_estimators=500)

Features (49): EState (C2), EState (C3), EState (R1), EState (R3), EState (R4), QH Entropy, HOMO, Hardness, LUMO, Mulliken (C3), Mulliken (O4), Mulliken (R1), Mulliken (R2), Mulliken (R3), Mulliken (R4), PBV (C1), PBV (C2), PBV (C3), PBV (R1), PBV (R2), PBV (R3), PBV (R4), PEOE (C1), PEOE (C2), PEOE (C3), PEOE (O4), PEOE (R3), PEOE (R4), Pint (C1), Pint (R2), Pint (R4), SASA (C1), SASA (C2), SASA (C3), SASA (R4), Sterimol B1 (R1), Sterimol B5 (R1), Sterimol L (R1), Sterimol B1 (R2), Sterimol B5 (R2), Sterimol L (R2), Sterimol B1 (R3), Sterimol B5 (R3), Sterimol L (R3), Sterimol B5 (R4), Sterimol L (R4), Vib. Freq., Vib. IR, ZPE

RFR, AM1 TS (RFECV)

- Train MAE: 1.36
- Test MAE: 1.44 ± 0.08
- Lit. 1 MAE: 0.87 ± 0.14
- Lit. 2 MAE: 1.16 ± 0.24
- Lit. 3 MAE: 1.14 ± 0.21
- Train R^2: 0.88
- Test R^2: 0.87
- Lit. 1 R^2: 0.33
- Lit. 2 R^2: 0.11
- Lit. 3 R^2: 0.15

Hyperparameters: RandomForestRegressor(max_depth=100, n_estimators=250)

Features (25): EState (C2), EState (C3), EState (R3), HOMO, Hardness, Mulliken (C1), Mulliken (C3), Mulliken (O4), Mulliken (R3), Mulliken (R4), PBV (C1), PBV (C3), PBV (C5), PBV (H6), PBV (H7), PBV (R3), PEOE (C1), PEOE (R4), SASA (C1), SASA (C5), SASA (H7), SASA (R4), Sterimol B1 (R1), Sterimol L (R3), Sterimol L (R4)

RFR, AM1 All (RFECV)

- Train MAE: 1.28
- Test MAE: 1.36 ± 0.08
- Lit. 1 MAE: 1.15 ± 0.15
- Lit. 2 MAE: 1.34 ± 0.2
- Lit. 3 MAE: 1.36 ± 0.21
- Train R^2: 0.9
- Test R^2: 0.88
- Lit. 1 R^2: 0.03
- Lit. 2 R^2: 0.17
- Lit. 3 R^2: -0.03

Hyperparameters: RandomForestRegressor(max_depth=50, n_estimators=500)

Features (26): Barrier, MA Mulliken (O4), MA PBV (R4), MA PEOE (C3), MA PEOE (O4), MA SASA (C1), MA SASA (R4), MA Vib. Freq., MA Vib. IR, TS EState (C3), TS EState (R3), TS EState (R4), TS HOMO, TS Hardness, TS Mulliken (C1), TS Mulliken (C3), TS Mulliken (H6), TS Mulliken (O4), TS Mulliken (R4), TS PBV (C5),

TS PBV (H6), TS PBV (R3), TS PEOE (R4), TS SASA (C5), TS SASA (H6), TS Sterimol B1 (R4)

RFR, PM6 MA (RFECV)

- Train MAE: 1.36
- Test MAE: 1.36 ± 0.08
- Lit. 1 MAE: 1.03 ± 0.13
- Lit. 2 MAE: 1.31 ± 0.22
- Lit. 3 MAE: 1.21 ± 0.19
- Train R^2: 0.88
- Test R^2: 0.88
- Lit. 1 R^2: 0.26
- Lit. 2 R^2: 0.09
- Lit. 3 R^2: 0.18

Hyperparameters: RandomForestRegressor(max_depth=50, n_estimators=500)

Features (28): EState (C2), EState (C3), EState (R3), EState (R4), Mulliken (C2), Mulliken (C3), Mulliken (O4), Mulliken (R1), Mulliken (R2), Mulliken (R3), Mulliken (R4), PBV (C1), PBV (C2), PBV (C3), PBV (R2), PBV (R3), PEOE (C3), PEOE (O4), PEOE (R4), SASA (C1), SASA (C3), SASA (R4), Sterimol B1 (R1), Sterimol B1 (R2), Sterimol B1 (R3), Sterimol B5 (R3), Sterimol L (R3), ZPE

RFR, PM6 TS (RFECV)

- Train MAE: 1.52
- Test MAE: 1.61 ± 0.09
- Lit. 1 MAE: 0.79 ± 0.14
- Lit. 2 MAE: 1.08 ± 0.24
- Lit. 3 MAE: 1.03 ± 0.2
- Train R^2: 0.85
- Test R^2: 0.84
- Lit. 1 R^2: 0.4
- Lit. 2 R^2: 0.14
- Lit. 3 R^2: 0.26

Hyperparameters: RandomForestRegressor(max_depth=100, n_estimators=500)

Features (32): Distance, EState (C2), EState (C3), EState (O4), EState (R3), EState (R4), HOMO, Mulliken (H6), Mulliken (O4), Mulliken (R3), Mulliken (R4), PBV (C1), PBV (C3), PBV (C5), PBV (H6), PBV (H7), PBV (O10), PBV (O4), PBV (R3), PEOE (C1), PEOE (O4), PEOE (R4), Pint (R4), SASA (C1), SASA (C5), SASA (R4), Softness, Sterimol B1 (R1), Sterimol L (R3), Sterimol L (R4), TPSA, ZPE

RFR, PM6 All (RFECV)

- Train MAE: 1.42
- Test MAE: 1.55 ± 0.08
- Lit. 1 MAE: 0.79 ± 0.15
- Lit. 2 MAE: 1.07 ± 0.24
- Lit. 3 MAE: 0.97 ± 0.2
- Train R^2: 0.87
- Test R^2: 0.86
- Lit. 1 R^2: 0.36
- Lit. 2 R^2: 0.18
- Lit. 3 R^2: 0.29

Hyperparameters: RandomForestRegressor(max_depth=50, n_estimators=250)

Features (26): Barrier, MA Mulliken (O4), MA PBV (C1), MA PEOE (C3), MA Pint (C1), MA Pint (R4), MA SASA (C1), MA SASA (C3), MA SASA (R4), TS Distance, TS EState (C2), TS EState (C3), TS EState (R3), TS EState (R4), TS HOMO, TS Mulliken (C1), TS Mulliken (C5), TS Mulliken (O4), TS Mulliken (R4), TS PBV (C3), TS PBV (C5), TS PBV (R3), TS PEOE (R4), TS SASA (C5), TS Sterimol B1 (R1), TS Sterimol L (R3)

RFR, DFT MA (RFECV)

- Train MAE: 1.3
- Test MAE: 1.3 ± 0.08
- Lit. 1 MAE: 0.93 ± 0.14
- Lit. 2 MAE: 1.16 ± 0.21
- Lit. 3 MAE: 1.2 ± 0.21
- Train R^2: 0.89
- Test R^2: 0.89
- Lit. 1 R^2: 0.29
- Lit. 2 R^2: 0.25
- Lit. 3 R^2: 0.09

Hyperparameters: RandomForestRegressor(max_depth=50, n_estimators=500)

Features (31): Chem. Pot., EState (C2), EState (C3), EState (R3), EState (R4), Electrophilicity, Hardness, LUMO, LogP (R4), Mulliken (C1), Mulliken (C3), Mulliken (O4), Mulliken (R3), Mulliken (R4), PBV (C1), PBV (C3), PBV (R1), PBV (R2), PBV (R3), PEOE (C1), PEOE (C3), PEOE (O4), PEOE (R4), SASA (C1), SASA (C2), SASA (R4), Sterimol B1 (R1), Sterimol L (R1), Sterimol B5 (R2), Sterimol L (R2), Sterimol L (R3)

RFR, AM1 PM6 All (RFECV)

- Train MAE: 1.3
- Test MAE: 1.33 ± 0.08
- Lit. 1 MAE: 1.13 ± 0.16
- Lit. 2 MAE: 1.3 ± 0.19
- Lit. 3 MAE: 1.36 ± 0.22
- Train R^2: 0.89
- Test R^2: 0.89
- Lit. 1 R^2: 0.02
- Lit. 2 R^2: 0.22
- Lit. 3 R^2: -0.04

Hyperparameters: RandomForestRegressor(max_depth=100, n_estimators=500)

Features (62): AM1 Barrier, AM1 MA Hardness, AM1 MA Mulliken (O4), AM1 MA Mulliken (R1), AM1 MA PBV (R4), AM1 MA SASA (C1), AM1 MA SASA (C3), AM1 MA SASA (R4), AM1 MA Sterimol L (R4), AM1 MA Vib. Freq., AM1 MA Vib. IR, AM1 TS EState (C3), AM1 TS HOMO, AM1 TS Hardness, AM1 TS Mulliken (C1), AM1 TS Mulliken (C3), AM1 TS Mulliken (H6), AM1 TS Mulliken (N8), AM1 TS Mulliken (O4), AM1 TS Mulliken (R4), AM1 TS PBV (C3), AM1 TS PBV (C5), AM1 TS PBV (H6), AM1 TS PBV (O10), AM1 TS PBV (R3), AM1 TS PEOE (C3), AM1 TS PEOE (O4), AM1 TS PEOE (R4), AM1 TS SASA (C5), AM1 TS SASA (H6), AM1 TS SASA (H7), AM1 TS SASA (N8), AM1 TS Sterimol B1 (R1), AM1 TS Sterimol B5 (R2), AM1 TS Sterimol B1 (R4), PM6 Barrier, PM6 MA Mulliken (O4), PM6 MA Mulliken (R3), PM6 MA PEOE (C3), PM6 MA PEOE (O4), PM6 MA SASA (C1), PM6 MA SASA (C3), PM6 MA SASA (R4), PM6 MA Sterimol B1 (R4), PM6 TS Distance, PM6 TS EState (C2), PM6 TS EState (C3), PM6 TS EState (R3), PM6 TS EState (R4), PM6 TS HOMO, PM6 TS Mulliken (C1), PM6 TS Mulliken (C5), PM6 TS Mulliken (H6), PM6 TS Mulliken (O4), PM6 TS Mulliken (R4), PM6 TS PBV (C5), PM6 TS PBV (H6), PM6 TS PBV (R1), PM6 TS PEOE (C1), PM6 TS SASA (C5), PM6 TS Sterimol B1 (R1), PM6 TS Sterimol L (R3)

GBR, UFF MA (RFECV)

- Train MAE: 1.17
- Test MAE: 1.15 ± 0.08
- Lit. 1 MAE: 1.08 ± 0.14
- Lit. 2 MAE: 1.33 ± 0.21
- Lit. 3 MAE: 1.32 ± 0.18

- Train R^2: 0.91
- Test R^2: 0.9
- Lit. 1 R^2: 0.18
- Lit. 2 R^2: 0.13
- Lit. 3 R^2: 0.15

Hyperparameters: GradientBoostingRegressor(max_depth=5, n_estimators=500)

Features (48): EState (C1), EState (C2), EState (C3), EState (R1), EState (R3), EState (R4), QH Entropy, MR (C2), MR (R3), Amide Bonds, PBV (C1), PBV (C2), PBV (C3), PBV (O4), PBV (R1), PBV (R2), PBV (R3), PBV (R4), PEOE (C1), PEOE (C3), PEOE (O4), PEOE (R1), PEOE (R2), PEOE (R3), PEOE (R4), Pint (R3), Pint (R4), SASA (C1), SASA (C2), SASA (C3), SASA (O4), SASA (R1), SASA (R2), SASA (R3), SASA (R4), Sterimol B1 (R1), Sterimol B5 (R1), Sterimol L (R1), Sterimol B1 (R2), Sterimol B5 (R2), Sterimol L (R2), Sterimol B1 (R3), Sterimol B5 (R3), Sterimol L (R3), Sterimol B1 (R4), Sterimol B5 (R4), Sterimol L (R4), ZPE

GBR, AM1 MA (RFECV)

- Train MAE: 1.15
- Test MAE: 1.18 ± 0.08
- Lit. 1 MAE: 1.08 ± 0.13
- Lit. 2 MAE: 1.31 ± 0.2
- Lit. 3 MAE: 1.21 ± 0.16
- Train R^2: 0.91
- Test R^2: 0.9
- Lit. 1 R^2: 0.2
- Lit. 2 R^2: 0.18
- Lit. 3 R^2: 0.28

Hyperparameters: GradientBoostingRegressor(max_depth=5, n_estimators=500)

Features (40): EState (C2), EState (C3), EState (R3), EState (R4), QH Entropy, HOMO, LUMO, MR (R3), Mulliken (C1), Mulliken (O4), Mulliken (R1), Mulliken (R2), Mulliken (R3), Mulliken (R4), PBV (C1), PBV (C2), PBV (C3), PBV (O4), PBV (R2), PBV (R3), PBV (R4), PEOE (C3), PEOE (O4), PEOE (R1), PEOE (R4), Pint (C1), Pint (R4), SASA (C1), SASA (C2), SASA (C3), SASA (R3), SASA (R4), Sterimol B1 (R1), Sterimol B5 (R1), Sterimol L (R1), Sterimol B1 (R3), Sterimol B5 (R3), Sterimol L (R3), Sterimol L (R4), Vib. IR

GBR, AM1 TS (RFECV)

- Train MAE: 1.22
- Test MAE: 1.28 ± 0.08
- Lit. 1 MAE: 1.13 ± 0.16
- Lit. 2 MAE: 1.33 ± 0.21
- Lit. 3 MAE: 1.29 ± 0.19
- Train R^2: 0.9
- Test R^2: 0.89
- Lit. 1 R^2: 0.02
- Lit. 2 R^2: 0.14
- Lit. 3 R^2: 0.13

Hyperparameters: GradientBoostingRegressor(max_depth=5, n_estimators=500)

Features (51): Chem. Pot., EState (C2), EState (C3), EState (O4), EState (R2), EState (R3), EState (R4), Electrophilicity, HOMO, Hardness, LUMO, MR (R3), MR (R4), Mulliken (C1), Mulliken (C2), Mulliken (C3), Mulliken (O4), Mulliken (R3), Mulliken (R4), PBV (C1), PBV (C2), PBV (C3), PBV (C5), PBV (H6), PBV (H7), PBV (O10), PBV (O4), PBV (R3), PBV (R4), PEOE (C1), PEOE (C2), PEOE (O4), PEOE (R2), PEOE (R4), Pint (C2), Pint (H7), SASA (C1), SASA (C3), SASA (C5), SASA (H6), SASA (H7), SASA (N8), SASA (R3), SASA (R4), Sterimol B1 (R1), Sterimol B5 (R1), Sterimol B1 (R3), Sterimol B5 (R3), Sterimol L (R3), Sterimol B1 (R4), Sterimol L (R4)

GBR, AM1 All (RFECV)

- Train MAE: 1.1
- Test MAE: 1.19 ± 0.08
- Lit. 1 MAE: 1.01 ± 0.16
- Lit. 2 MAE: 1.13 ± 0.17
- Lit. 3 MAE: 1.2 ± 0.21
- Train R^2: 0.92
- Test R^2: 0.9
- Lit. 1 R^2: 0.15
- Lit. 2 R^2: 0.38
- Lit. 3 R^2: 0.11

Hyperparameters: GradientBoostingRegressor(max_depth=5, n_estimators=500)

Features (58): Barrier, MA Mulliken (C1), MA Mulliken (O4), MA Mulliken (R1), MA Mulliken (R2), MA Mulliken (R3), MA PBV (R4), MA PEOE (C3), MA PEOE (O4), MA PEOE (R3), MA Pint (C2), MA Pint (R1), MA Pint (R4), MA SASA (C1), MA SASA (C3), MA SASA (R4), MA Sterimol B1 (R1), MA Sterimol B1 (R2), MA Sterimol B1 (R3), MA Sterimol B5 (R3), MA Sterimol B1 (R4), MA Vib. IR, TS Chem. Pot., TS EState (C2), TS EState (C3), TS EState (R3), TS EState (R4), TS HOMO, TS Hardness, TS MR (R3), TS Mulliken (C1), TS Mulliken (C5), TS Mulliken (H6), TS Mulliken (N8), TS Mulliken (O4), TS Mulliken (R3), TS Mulliken (R4), TS PBV (C2), TS PBV (C3), TS PBV (C5), TS PBV (H6), TS PBV (H7), TS PBV (R3), TS PEOE (C1), TS PEOE (R2), TS PEOE (R4), TS Pint (H6), TS SASA (C5), TS SASA (H6), TS SASA (H7), TS SASA (N8), TS SASA (O10), TS SASA (O9), TS Sterimol B5 (R2), TS Sterimol B1 (R3), TS Sterimol B5 (R3), TS Sterimol L (R3), TS Sterimol B1 (R4)

GBR, PM6 MA (RFECV)

- Train MAE: 1.27
- Test MAE: 1.27 ± 0.08
- Lit. 1 MAE: 1.22 ± 0.14
- Lit. 2 MAE: 1.47 ± 0.22
- Lit. 3 MAE: 1.39 ± 0.18
- Train R^2: 0.89
- Test R^2: 0.89
- Lit. 1 R^2: 0.03
- Lit. 2 R^2: -0.0
- Lit. 3 R^2: 0.11

Hyperparameters: GradientBoostingRegressor(max_depth=5, n_estimators=500)

Features (61): Chem. Pot., EState (C1), EState (C2), EState (C3), EState (R1), EState (R2), EState (R3), EState (R4), Energy, Enthalpy, HOMO, LogP (R4), MR (R3), Mulliken (C1), Mulliken (C2), Mulliken (C3), Mulliken (O4), Mulliken (R1), Mulliken (R2), Mulliken (R3), Mulliken (R4), PBV (C1), PBV (C2), PBV (C3), PBV (O4), PBV (R1), PBV (R2), PBV (R3), PBV (R4), PEOE (C1), PEOE (C2), PEOE (C3), PEOE (O4), PEOE (R1), PEOE (R2), PEOE (R3), PEOE (R4), Pint (C1), Pint (C2), Pint (O4), Pint (R1), Pint (R2), Pint (R4), SASA (C1), SASA (C2), SASA (C3), SASA (O4), SASA (R3), SASA (R4), Softness, Sterimol B1 (R1), Sterimol B5 (R1), Sterimol B1 (R2), Sterimol B5 (R2), Sterimol L (R2), Sterimol B1 (R3), Sterimol L (R3), Sterimol B1 (R4), Sterimol L (R4), Vib. Freq., Vib. IR

GBR, PM6 TS (RFECV)

- Train MAE: 1.33
- Test MAE: 1.33 ± 0.08
- Lit. 1 MAE: 0.95 ± 0.15
- Lit. 2 MAE: 1.19 ± 0.22
- Lit. 3 MAE: 1.1 ± 0.18

- Train R^2: 0.88
- Test R^2: 0.89
- Lit. 1 R^2: 0.23
- Lit. 2 R^2: 0.18
- Lit. 3 R^2: 0.29

Hyperparameters: GradientBoostingRegressor(max_depth=5, n_estimators=500)

Features (55): Chem. Pot., EState (C2), EState (C3), EState (R2), EState (R3), EState (R4), HOMO, LUMO, MR (R3), MR (R4), Mulliken (C1), Mulliken (C5), Mulliken (H6), Mulliken (N8), Mulliken (O4), Mulliken (R2), Mulliken (R3), Mulliken (R4), PBV (C1), PBV (C2), PBV (C3), PBV (C5), PBV (H6), PBV (O9), PBV (R1), PBV (R3), PEOE (C1), PEOE (C3), PEOE (O4), PEOE (R1), PEOE (R2), PEOE (R3), PEOE (R4), Pint (C1), Pint (C5), Pint (H7), Pint (R2), Pint (R4), SASA (C1), SASA (C3), SASA (C5), SASA (N8), SASA (O4), SASA (R2), SASA (R4), Softness, Sterimol B1 (R1), Sterimol B5 (R2), Sterimol B1 (R3), Sterimol L (R3), Sterimol B1 (R4), Sterimol B5 (R4), Sterimol L (R4), TPSA, Vib. IR

GBR, PM6 All (RFECV)

- Train MAE: 1.29
- Test MAE: 1.25 ± 0.07
- Lit. 1 MAE: 0.9 ± 0.17
- Lit. 2 MAE: 1.15 ± 0.24
- Lit. 3 MAE: 1.07 ± 0.2
- Train R^2: 0.89
- Test R^2: 0.9
- Lit. 1 R^2: 0.18
- Lit. 2 R^2: 0.12
- Lit. 3 R^2: 0.25

Hyperparameters: GradientBoostingRegressor(max_depth=5, n_estimators=500)

Features (56): Barrier, MA EState (C1), MA Mulliken (C2), MA Mulliken (O4), MA Mulliken (R1), MA Mulliken (R3), MA PBV (C1), MA PBV (O4), MA PBV (R1), MA PEOE (C3), MA PEOE (O4), MA Pint (C1), MA Pint (R1), MA Pint (R4), MA SASA (C1), MA SASA (C3), MA SASA (O4), MA SASA (R4), MA Sterimol B1 (R1), MA Sterimol B5 (R1), MA Sterimol B5 (R3), MA Sterimol B1 (R4), MA Sterimol L (R4), MA TPSA, MA Vib. Freq., TS Distance, TS EState (C3), TS EState (R2), TS EState (R3), TS EState (R4), TS HOMO, TS MR (R3), TS Mulliken (C1), TS Mulliken (C3), TS Mulliken (C5), TS Mulliken (H6), TS Mulliken (H7), TS Mulliken (N8), TS Mulliken (O4), TS PBV (C2), TS PBV (C3), TS PBV (C5), TS PBV (H6), TS PBV (O10), TS PBV (R3), TS PEOE (C1), TS PEOE (R1), TS PEOE (R4), TS Pint (N8), TS SASA (C5), TS SASA (N8), TS SASA (R3), TS Sterimol B1 (R1), TS Sterimol B5 (R2), TS Sterimol L (R3), TS Vib. IR

GBR, DFT MA (RFECV)

- Train MAE: 1.16
- Test MAE: 1.12 ± 0.08
- Lit. 1 MAE: 0.88 ± 0.14
- Lit. 2 MAE: 1.13 ± 0.21
- Lit. 3 MAE: 1.13 ± 0.2
- Train R^2: 0.91
- Test R^2: 0.91
- Lit. 1 R^2: 0.36
- Lit. 2 R^2: 0.24
- Lit. 3 R^2: 0.18

Hyperparameters: GradientBoostingRegressor(max_depth=5, n_estimators=500)

Features (64): Chem. Pot., EState (C1), EState (C2), EState (C3), EState (R1), EState (R2), EState (R3), EState (R4), Electrophilicity, Enthalpy, LUMO, LogP (R4), MR (R2), MR (R3), Mulliken (C1), Mulliken (C2), Mulliken (C3), Mulliken (O4), Mulliken (R1), Mulliken (R3), Mulliken (R4), PBV (C1), PBV (C2), PBV (C3), PBV (O4), PBV (R1), PBV (R2), PBV (R3), PBV (R4), PEOE (C1), PEOE (C3), PEOE (O4), PEOE (R2), PEOE (R3), PEOE (R4), Pint (C1), Pint (C2), Pint (C3), Pint (O4), Pint (R1), Pint (R2), Pint (R3), Pint (R4), Surface Area, SASA (C1), SASA (C2), SASA (C3), SASA (O4), SASA (R3), SASA (R4), Sterimol B1 (R1), Sterimol L (R1), Sterimol B1 (R2), Sterimol B5 (R2), Sterimol L (R2), Sterimol B1 (R3), Sterimol B5 (R3), Sterimol L (R3), Sterimol B1 (R4), Sterimol B5 (R4), Sterimol L (R4), Vib. Freq., Vib. IR, ZPE

GBR, AM1 PM6 All (RFECV)

- Train MAE: 1.12
- Test MAE: 1.2 ± 0.07
- Lit. 1 MAE: 1.01 ± 0.16
- Lit. 2 MAE: 1.15 ± 0.18
- Lit. 3 MAE: 1.24 ± 0.22
- Train R^2: 0.92
- Test R^2: 0.91
- Lit. 1 R^2: 0.14
- Lit. 2 R^2: 0.36
- Lit. 3 R^2: 0.05

Hyperparameters: GradientBoostingRegressor(max_depth=5, n_estimators=500)

Features (38): AM1 Barrier, AM1 MA Mulliken (C1), AM1 MA PBV (R4), AM1 MA Pint (R4), AM1 MA SASA (C1), AM1 MA SASA (R4), AM1 MA Sterimol B1 (R3), AM1 MA Vib. IR, AM1 TS HOMO, AM1 TS Hardness, AM1 TS Mulliken (C1), AM1 TS Mulliken (H6), AM1 TS Mulliken (O4), AM1 TS Mulliken (R4), AM1 TS PBV (C3), AM1 TS PBV (C5), AM1 TS PBV (H6), AM1 TS PBV (R3), AM1 TS PEOE (R4), AM1 TS SASA (C5), AM1 TS SASA (R2), AM1 TS Sterimol B5 (R3), AM1 TS Sterimol L (R3), PM6 Barrier, PM6 MA Mulliken (O4), PM6 MA PBV (O4), PM6 MA PEOE (C3), PM6 MA PEOE (O4), PM6 MA SASA (C3), PM6 MA SASA (R4), PM6 TS EState (C3), PM6 TS EState (R3), PM6 TS HOMO, PM6 TS Mulliken (C5), PM6 TS PBV (C5), PM6 TS PBV (R3), PM6 TS PEOE (C1), PM6 TS SASA (C5)

SVR(RBF), UFF MA (SFS)

- Train MAE: 0.94
- Test MAE: 0.98 ± 0.07
- Lit. 1 MAE: 1.26 ± 0.17
- Lit. 2 MAE: 1.56 ± 0.26
- Lit. 3 MAE: 1.52 ± 0.24
- Train R^2: 0.94
- Test R^2: 0.92
- Lit. 1 R^2: -0.17
- Lit. 2 R^2: -0.26
- Lit. 3 R^2: -0.31

Hyperparameters: SVR(C=10, epsilon=0.25)

Features (30): EState (C2), EState (R1), EState (R2), EState (R3), EState (R4), HB Acceptors, HB Donors, LogP (R1), LogP (R2), LogP (R4), MR (R4), Amide Bonds, PBV (C1), PBV (O4), PEOE (C1), PEOE (C2), PEOE (O4), PEOE (R2), PEOE (R3), Pint (C2), Pint (R2), Pint (R4), SASA (C2), SASA (R2), Sterimol B5 (R1), Sterimol B1 (R3), Sterimol B5 (R3), Sterimol L (R3), Sterimol B1 (R4), Sterimol L (R4)

SVR(RBF), AM1 MA (SFS)

- Train MAE: 0.96
- Test MAE: 0.95 ± 0.08
- Lit. 1 MAE: 1.27 ± 0.16
- Lit. 2 MAE: 1.54 ± 0.24
- Lit. 3 MAE: 1.49 ± 0.22

- Train R^2: 0.94
- Test R^2: 0.92
- Lit. 1 R^2: -0.16
- Lit. 2 R^2: -0.17
- Lit. 3 R^2: -0.15

Hyperparameters: SVR(C=10)

Features (36): EState (C2), EState (R3), EState (R4), HB Donors, Hardness, LogP (O4), LogP (R2), LogP (R3), LogP (R4), MR (R2), MR (R4), Mulliken (O4), Mulliken (R1), Mulliken (R2), Mulliken (R3), Amide Bonds, PBV (C1), PBV (C2), PBV (C3), PBV (R2), PEOE (C1), PEOE (C2), PEOE (C3), PEOE (O4), PEOE (R2), PEOE (R3), Pint (C1), Pint (C2), Pint (R4), SASA (R1), SASA (R2), SASA (R4), Sterimol B5 (R3), Sterimol L (R3), Sterimol B1 (R4), Vib. IR

SVR(RBF), AM1 TS (SFS)

- Train MAE: 0.92
- Test MAE: 0.93 ± 0.06
- Lit. 1 MAE: 1.14 ± 0.16
- Lit. 2 MAE: 1.42 ± 0.25
- Lit. 3 MAE: 1.34 ± 0.2
- Train R^2: 0.94
- Test R^2: 0.94
- Lit. 1 R^2: 0.02
- Lit. 2 R^2: -0.1
- Lit. 3 R^2: 0.04

Hyperparameters: SVR(C=10)

Features (33): EState (O4), EState (R2), EState (R3), EState (R4), HB Donors, HOMO, Hardness, LogP (R1), LogP (R3), LogP (R4), MR (R2), MR (R3), MR (R4), Mulliken (H6), Mulliken (R1), Mulliken (R3), Amide Bonds, PBV (C1), PBV (C5), PEOE (C3), PEOE (R2), PEOE (R3), Pint (R4), SASA (C2), SASA (C5), SASA (N8), SASA (O4), SASA (R1), SASA (R2), Sterimol B5 (R2), Sterimol B5 (R3), Sterimol L (R3), Sterimol L (R4)

SVR(RBF), AM1 All (SFS)

- Train MAE: 0.91
- Test MAE: 0.96 ± 0.07
- Lit. 1 MAE: 1.05 ± 0.14
- Lit. 2 MAE: 1.37 ± 0.25
- Lit. 3 MAE: 1.3 ± 0.21
- Train R^2: 0.94
- Test R^2: 0.93
- Lit. 1 R^2: 0.2
- Lit. 2 R^2: -0.09
- Lit. 3 R^2: 0.02

Hyperparameters: SVR(C=5)

Features (60): Barrier, MA HOMO, MA Mulliken (C1), MA Mulliken (O4), MA Mulliken (R1), MA Mulliken (R2), MA Mulliken (R3), MA PBV (R2), MA PBV (R4), MA PEOE (C3), MA PEOE (O4), MA PEOE (R3), MA Pint (C2), MA Pint (R4), MA SASA (O4), MA SASA (R1), MA SASA (R4), MA Sterimol B1 (R1), MA Sterimol B5 (R1), MA Sterimol B5 (R3), MA Sterimol B1 (R4), MA Sterimol L (R4), MA TPSA, MA Vib. IR, TS EState (C3), TS EState (O4), TS EState (R2), TS EState (R3), TS EState (R4), TS HB Acceptors, TS HB Donors, TS HOMO, TS Hardness, TS LogP (R2), TS LogP (R4), TS MR (R2), TS MR (R3), TS MR (R4), TS Mulliken (C3), TS Mulliken (N8), TS Mulliken (O4), TS Mulliken (R3), TS Amide Bonds, TS PBV (C3), TS PBV (C5), TS PBV (H6), TS PBV (H7), TS PBV (O4), TS PEOE (C1), TS PEOE (C2), TS PEOE (R2), TS Pint (O4), TS SASA (C2), TS SASA (C5), TS SASA (N8), TS Sterimol B5 (R2), TS Sterimol B5 (R3), TS Sterimol L (R3), TS Sterimol B1 (R4), TS Sterimol B5 (R4)

SVR(RBF), PM6 MA (SFS)

- Train MAE: 0.97
- Test MAE: 1.02 ± 0.08
- Lit. 1 MAE: 1.55 ± 0.22
- Lit. 2 MAE: 1.87 ± 0.31
- Lit. 3 MAE: 1.8 ± 0.27
- Train R^2: 0.93
- Test R^2: 0.91
- Lit. 1 R^2: -0.9
- Lit. 2 R^2: -0.79
- Lit. 3 R^2: -0.76

Hyperparameters: SVR(C=10)

Features (20): EState (C3), EState (R2), Enthalpy, HB Donors, LogP (R3), LogP (R4), Mulliken (O4), Mulliken (R3), Amide Bonds, PBV (C1), PBV (C2), PBV (C3), PBV (R2), PEOE (R2), PEOE (R3), Pint (O4), SASA (C2), SASA (O4), SASA (R1), SASA (R2)

SVR(RBF), PM6 TS (SFS)

- Train MAE: 0.92
- Test MAE: 0.98 ± 0.07
- Lit. 1 MAE: 1.14 ± 0.13
- Lit. 2 MAE: 1.45 ± 0.24
- Lit. 3 MAE: 1.36 ± 0.19
- Train R^2: 0.94
- Test R^2: 0.93
- Lit. 1 R^2: 0.12
- Lit. 2 R^2: -0.1
- Lit. 3 R^2: 0.06

Hyperparameters: SVR(C=10)

Features (30): EState (R2), EState (R3), EState (R4), Energy, HB Acceptors, HB Donors, HOMO, LogP (R1), MR (R2), MR (R3), MR (R4), Mulliken (C2), Mulliken (R1), Mulliken (R3), Mulliken (R4), Amide Bonds, PBV (C1), PBV (C3), PBV (C5), PEOE (C3), PEOE (O4), PEOE (R1), PEOE (R2), PEOE (R3), SASA (C2), SASA (C5), SASA (R2), Sterimol B5 (R2), Sterimol L (R4), Vib. Freq.

SVR(RBF), PM6 All (SFS)

- Train MAE: 0.94
- Test MAE: 1.03 ± 0.08
- Lit. 1 MAE: 1.22 ± 0.15
- Lit. 2 MAE: 1.48 ± 0.23
- Lit. 3 MAE: 1.37 ± 0.18
- Train R^2: 0.93
- Test R^2: 0.91
- Lit. 1 R^2: -0.04
- Lit. 2 R^2: -0.08
- Lit. 3 R^2: 0.11

Hyperparameters: SVR(C=10)

Features (22): MA Mulliken (O4), MA Mulliken (R3), MA PBV (C1), MA PBV (R2), MA PEOE (C3), MA PEOE (R3), MA Pint (C3), MA Pint (R4), MA SASA (O4), MA SASA (R1), MA Sterimol B5 (R3), TS EState (R2), TS HB Donors, TS MR (R2), TS MR (R3), TS MR (R4), TS Mulliken (C2), TS Amide Bonds, TS PBV (C5), TS PEOE (R2), TS SASA (C2), TS Vib. Freq.

SVR(RBF), DFT MA (SFS)

- Train MAE: 0.93
- Test MAE: 0.93 ± 0.07
- Lit. 1 MAE: 1.48 ± 0.19
- Lit. 2 MAE: 1.69 ± 0.23
- Lit. 3 MAE: 1.6 ± 0.2
- Train R^2: 0.94

- Test R^2: 0.93
- Lit. 1 R^2: -0.59
- Lit. 2 R^2: -0.24
- Lit. 3 R^2: -0.18

Hyperparameters: SVR(C=10)

Features (31): EState (R2), EState (R3), EState (R4), HB Donors, LUMO, LogP (O4), LogP (R1), LogP (R2), LogP (R4), MR (R2), MR (R3), MR (R4), Mulliken (C3), Mulliken (O4), Mulliken (R3), Amide Bonds, PBV (C1), PBV (C2), PEOE (C1), PEOE (C2), PEOE (C3), PEOE (R2), PEOE (R3), Pint (C1), Pint (O4), SASA (C2), SASA (R2), SASA (R4), Sterimol B1 (R1), Sterimol B5 (R2), Sterimol B1 (R4)

SVR(RBF), AM1 PM6 All (SFS)

- Train MAE: 0.88
- Test MAE: 0.98 ± 0.07
- Lit. 1 MAE: 1.03 ± 0.14
- Lit. 2 MAE: 1.29 ± 0.22
- Lit. 3 MAE: 1.19 ± 0.17
- Train R^2: 0.94
- Test R^2: 0.93
- Lit. 1 R^2: 0.23
- Lit. 2 R^2: 0.11
- Lit. 3 R^2: 0.27

Hyperparameters: SVR(C=5)

Features (58): AM1 Barrier, AM1 MA Mulliken (O4), AM1 MA Mulliken (R1), AM1 MA Mulliken (R2), AM1 MA PBV (R4), AM1 MA Pint (R4), AM1 MA SASA (R4), AM1 MA Sterimol B1 (R1), AM1 MA Sterimol B5 (R1), AM1 MA Sterimol B1 (R4), AM1 MA Sterimol L (R4), AM1 MA Vib. IR, AM1 TS Mulliken (C3), AM1 TS Mulliken (C5), AM1 TS Mulliken (N8), AM1 TS Mulliken (O4), AM1 TS Mulliken (R3), AM1 TS PBV (C3), AM1 TS PBV (C5), AM1 TS PEOE (C2), AM1 TS PEOE (C3), AM1 TS PEOE (R1), AM1 TS PEOE (R2), AM1 TS PEOE (R3), AM1 TS Pint (C5), AM1 TS Pint (O9), AM1 TS SASA (C2), AM1 TS SASA (C5), AM1 TS SASA (R2), PM6 MA Mulliken (C2), PM6 MA Mulliken (O4), PM6 MA Mulliken (R3), PM6 MA PBV (C1), PM6 MA PEOE (R3), PM6 MA Pint (R4), PM6 MA SASA (O4), PM6 MA Sterimol B1 (R3), PM6 MA Sterimol B1 (R4), PM6 MA Sterimol L (R4), PM6 MA TPSA, PM6 TS EState (R2), PM6 TS EState (R4), PM6 TS HB Donors, PM6 TS HOMO, PM6 TS LogP (R2), PM6 TS LogP (R4), PM6 TS MR (R2), PM6 TS MR (R3), PM6 TS MR (R4), PM6 TS Mulliken (C2), PM6 TS Amide Bonds, PM6 TS PBV (R1), PM6 TS PEOE (C1), PM6 TS PEOE (R2), PM6 TS SASA (C2), PM6 TS SASA (C5), PM6 TS SASA (O4), PM6 TS Vib. Freq.

SVR(poly), UFF MA (SFS)

- Train MAE: 1.03
- Test MAE: 1.05 ± 0.07
- Lit. 1 MAE: 1.96 ± 0.23
- Lit. 2 MAE: 2.28 ± 0.31
- Lit. 3 MAE: 2.26 ± 0.3
- Train R^2: 0.93
- Test R^2: 0.92
- Lit. 1 R^2: -1.55
- Lit. 2 R^2: -1.24
- Lit. 3 R^2: -1.45

Hyperparameters: SVR(C=0.1, coef0=4, degree=4, epsilon=0.25, kernel='poly')

Features (22): EState (C2), EState (C3), EState (R3), EState (R4), HB Donors, LogP (R3), LogP (R4), MR (C2), MR (R3), MR (R4), Amide Bonds, PBV (C3), PEOE (C1), PEOE (C2), PEOE (R2), PEOE (R3), Pint (C1), SASA (R3), SASA (R4), Sterimol B5 (R2), Sterimol L (R2), Sterimol B1 (R3)

SVR(poly), AM1 MA (SFS)

- Train MAE: 1.03
- Test MAE: 1.12 ± 0.07
- Lit. 1 MAE: 1.51 ± 0.2
- Lit. 2 MAE: 1.8 ± 0.27
- Lit. 3 MAE: 1.73 ± 0.24
- Train R^2: 0.93
- Test R^2: 0.91
- Lit. 1 R^2: -0.64
- Lit. 2 R^2: -0.55
- Lit. 3 R^2: -0.49

Hyperparameters: SVR(C=2, coef0=1, epsilon=0.25, kernel='poly')

Features (29): EState (C2), EState (C3), EState (R3), HB Acceptors, HB Donors, HOMO, LogP (R3), LogP (R4), MR (C2), MR (R3), MR (R4), Mulliken (C1), Mulliken (O4), Mulliken (R3), Amide Bonds, PBV (C1), PBV (C3), PBV (R3), PEOE (C1), PEOE (C2), PEOE (O4), PEOE (R2), PEOE (R3), Pint (C1), Pint (R4), SASA (R3), SASA (R4), Sterimol B5 (R2), Sterimol B1 (R3)

SVR(poly), AM1 TS (SFS)

- Train MAE: 1.01
- Test MAE: 1.06 ± 0.07
- Lit. 1 MAE: 1.17 ± 0.18
- Lit. 2 MAE: 1.25 ± 0.18
- Lit. 3 MAE: 1.12 ± 0.17
- Train R^2: 0.93
- Test R^2: 0.92
- Lit. 1 R^2: -0.12
- Lit. 2 R^2: 0.29
- Lit. 3 R^2: 0.32

Hyperparameters: SVR(C=0.5, coef0=2, degree=4, epsilon=0.25, kernel='poly')

Features (20): EState (R2), EState (R3), HB Acceptors, HB Donors, HOMO, LogP (R2), LogP (R3), Mulliken (C5), Mulliken (O4), Amide Bonds, PBV (C3), PBV (C5), PBV (R1), PBV (R2), PEOE (C1), PEOE (R3), PEOE (R4), SASA (C5), SASA (R3), SASA (R4)

SVR(poly), AM1 All (SFS)

- Train MAE: 0.97
- Test MAE: 0.95 ± 0.06
- Lit. 1 MAE: 1.22 ± 0.18
- Lit. 2 MAE: 1.54 ± 0.28
- Lit. 3 MAE: 1.54 ± 0.28
- Train R^2: 0.94
- Test R^2: 0.93
- Lit. 1 R^2: -0.17
- Lit. 2 R^2: -0.36
- Lit. 3 R^2: -0.54

Hyperparameters: SVR(C=0.1, coef0=3, degree=4, epsilon=0.25, kernel='poly')

Features (36): Barrier, MA HOMO, MA Hardness, MA MR (C1), MA Mulliken (C1), MA Mulliken (O4), MA Mulliken (R2), MA Mulliken (R3), MA PBV (R4), MA PEOE (C3), MA PEOE (R3), MA Pint (C1), MA Pint (R4), MA SASA (R4), MA TPSA, TS EState (C3), TS EState (R3), TS HB Acceptors, TS HB Donors, TS HOMO, TS Hardness, TS LogP (R3), TS MR (R3), TS MR (R4), TS Mulliken (N8), TS Mulliken (R3), TS Mulliken (R4), TS Amide Bonds, TS PBV (C3), TS PBV (C5), TS PEOE (C1), TS PEOE (R4), TS SASA (C5), TS SASA (R3), TS Sterimol B5 (R2), TS Sterimol L (R3)

SVR(poly), PM6 MA (SFS)

- Train MAE: 0.99
- Test MAE: 1.11 ± 0.08
- Lit. 1 MAE: 1.68 ± 0.24
- Lit. 2 MAE: 1.84 ± 0.25
- Lit. 3 MAE: 1.78 ± 0.23
- Train R^2: 0.93
- Test R^2: 0.91
- Lit. 1 R^2: -1.16
- Lit. 2 R^2: -0.46
- Lit. 3 R^2: -0.5

Hyperparameters: SVR(C=0.5, coef0=1, degree=4, epsilon=0.25, kernel='poly')

Features (23): EState (C3), EState (R3), HB Acceptors, HB Donors, LogP (R3), LogP (R4), MR (C2), MR (R4), Mulliken (O4), Mulliken (R3), Amide Bonds, PBV (C1), PBV (C3), PEOE (C1), PEOE (C3), PEOE (R2), PEOE (R3), Pint (C1), Pint (R4), SASA (R3), SASA (R4), Softness, Sterimol B1 (R3)

SVR(poly), PM6 TS (SFS)

- Train MAE: 1.09
- Test MAE: 1.07 ± 0.07
- Lit. 1 MAE: 1.68 ± 0.23
- Lit. 2 MAE: 1.86 ± 0.25
- Lit. 3 MAE: 1.73 ± 0.22
- Train R^2: 0.92
- Test R^2: 0.92
- Lit. 1 R^2: -1.14
- Lit. 2 R^2: -0.48
- Lit. 3 R^2: -0.41

Hyperparameters: SVR(C=0.1, coef0=2, degree=5, epsilon=0.5, kernel='poly')

Features (22): EState (R2), EState (R3), EState (R4), HB Acceptors, HB Donors, HOMO, LogP (R2), LogP (R3), MR (R2), MR (R4), Mulliken (O4), Mulliken (R4), Amide Bonds, PBV (C1), PBV (C3), PBV (C5), PEOE (C1), PEOE (C2), SASA (R3), SASA (R4), Sterimol L (R1), Sterimol L (R2)

SVR(poly), PM6 All (SFS)

- Train MAE: 1.04
- Test MAE: 1.01 ± 0.07
- Lit. 1 MAE: 1.18 ± 0.18
- Lit. 2 MAE: 1.46 ± 0.26
- Lit. 3 MAE: 1.34 ± 0.21
- Train R^2: 0.92
- Test R^2: 0.93
- Lit. 1 R^2: -0.17
- Lit. 2 R^2: -0.18
- Lit. 3 R^2: 0.02

Hyperparameters: SVR(C=0.1, coef0=4, degree=4, epsilon=0.25, kernel='poly')

Features (31): Barrier, MA MR (C2), MA Mulliken (O4), MA Mulliken (R3), MA PBV (C1), MA PBV (R1), MA PEOE (R3), MA Pint (R4), MA SASA (R4), MA Sterimol B1 (R3), MA Sterimol B5 (R3), TS EState (R2), TS EState (R3), TS HB Acceptors, TS HB Donors, TS HOMO, TS LogP (R2), TS LogP (R3), TS MR (R3), TS MR (R4), TS Mulliken (N8), TS Mulliken (R4), TS Amide Bonds, TS PBV (C3), TS PBV (C5), TS PBV (R3), TS PBV (R4), TS PEOE (C1), TS PEOE (R4), TS Pint (H6), TS SASA (R3)

SVR(poly), DFT MA (SFS)

- Train MAE: 0.99
- Test MAE: 1.03 ± 0.07
- Lit. 1 MAE: 2.25 ± 0.29
- Lit. 2 MAE: 2.51 ± 0.32
- Lit. 3 MAE: 2.31 ± 0.27
- Train R^2: 0.93
- Test R^2: 0.92
- Lit. 1 R^2: -2.59
- Lit. 2 R^2: -1.61
- Lit. 3 R^2: -1.36

Hyperparameters: SVR(C=0.1, coef0=4, degree=4, epsilon=0.25, kernel='poly')

Features (23): EState (R2), EState (R3), HB Donors, LUMO, LogP (R2), LogP (R3), LogP (R4), MR (C1), MR (R4), Mulliken (C1), Mulliken (O4), Mulliken (R3), Amide Bonds, PBV (C1), PBV (C3), PEOE (C2), Pint (R3), Pint (R4), SASA (R3), SASA (R4), Sterimol B1 (R3), Sterimol B5 (R3), Sterimol L (R4)

SVR(poly), AM1 PM6 All (SFS)

- Train MAE: 0.98
- Test MAE: 0.98 ± 0.07
- Lit. 1 MAE: 1.29 ± 0.19
- Lit. 2 MAE: 1.53 ± 0.24
- Lit. 3 MAE: 1.42 ± 0.2
- Train R^2: 0.93
- Test R^2: 0.93
- Lit. 1 R^2: -0.3
- Lit. 2 R^2: -0.16
- Lit. 3 R^2: -0.02

Hyperparameters: SVR(C=0.1, coef0=3, degree=4, epsilon=0.25, kernel='poly')

Features (51): AM1 Barrier, AM1 MA LUMO, AM1 MA Mulliken (C1), AM1 MA Mulliken (O4), AM1 MA Mulliken (R2), AM1 MA Mulliken (R3), AM1 MA PBV (R4), AM1 MA Pint (C1), AM1 MA Pint (R4), AM1 MA SASA (R4), AM1 MA Sterimol B5 (R3), AM1 TS Electrophilicity, AM1 TS Mulliken (C3), AM1 TS Mulliken (R3), AM1 TS PBV (C3), AM1 TS PBV (C5), AM1 TS PBV (H7), AM1 TS PBV (R3), AM1 TS PEOE (R3), AM1 TS PEOE (R4), AM1 TS SASA (H6), AM1 TS SASA (N8), AM1 TS SASA (R3), AM1 TS Vib. IR, PM6 MA MR (C1), PM6 MA MR (C2), PM6 MA Mulliken (O4), PM6 MA Mulliken (R3), PM6 MA PEOE (O4), PM6 MA PEOE (R3), PM6 MA Pint (R4), PM6 MA SASA (R4), PM6 MA Softness, PM6 MA Sterimol B1 (R4), PM6 MA Sterimol B5 (R4), PM6 MA Sterimol L (R4), PM6 MA TPSA, PM6 TS EState (R3), PM6 TS HB Acceptors, PM6 TS HB Donors, PM6 TS HOMO, PM6 TS LogP (R3), PM6 TS MR (R3), PM6 TS MR (R4), PM6 TS Mulliken (R4), PM6 TS Amide Bonds, PM6 TS PBV (C5), PM6 TS PBV (R3), PM6 TS PEOE (C1), PM6 TS PEOE (C2), PM6 TS SASA (R3)

KRR(RBF), UFF MA (SFS)

- Train MAE: 0.98
- Test MAE: 1.0 ± 0.07
- Lit. 1 MAE: 1.77 ± 0.3
- Lit. 2 MAE: 1.89 ± 0.29
- Lit. 3 MAE: 1.84 ± 0.28
- Train R^2: 0.93
- Test R^2: 0.92
- Lit. 1 R^2: -1.79
- Lit. 2 R^2: -0.72
- Lit. 3 R^2: -0.86

Hyperparameters: KernelRidge(alpha=0.1, kernel='rbf')

Features (25): EState (C2), EState (R1), EState (R2), EState (R3), EState (R4), HB Donors, LogP (R2), LogP (R3), MR (R2), MR (R4), Amide Bonds, PBV (C1), PBV (C2), PEOE (C1), PEOE (C3), PEOE

(R2), PEOE (R3), Pint (C2), Pint (C3), SASA (C2), SASA (C3), SASA (R1), SASA (R3), Sterimol B5 (R3), Sterimol L (R3)

KRR(RBF), AM1 MA (SFS)

- Train MAE: 0.99
- Test MAE: 1.0 ± 0.07
- Lit. 1 MAE: 1.58 ± 0.24
- Lit. 2 MAE: 1.64 ± 0.24
- Lit. 3 MAE: 1.58 ± 0.23
- Train R^2: 0.93
- Test R^2: 0.92
- Lit. 1 R^2: -1.08
- Lit. 2 R^2: -0.22
- Lit. 3 R^2: -0.31

Hyperparameters: KernelRidge(alpha=0.1, kernel='rbf')

Features (35): EState (C2), EState (R2), EState (R3), EState (R4), HB Acceptors, HB Donors, LogP (R2), LogP (R3), MR (C2), MR (R2), MR (R3), MR (R4), Mulliken (C3), Mulliken (O4), Mulliken (R1), Mulliken (R3), Amide Bonds, PBV (C1), PBV (C2), PEOE (C1), PEOE (C2), PEOE (C3), PEOE (R3), Pint (C1), Pint (C3), Pint (R4), SASA (C2), SASA (C3), SASA (R1), SASA (R2), SASA (R3), Sterimol B1 (R1), Sterimol B5 (R3), Sterimol L (R3), Sterimol B1 (R4)

KRR(RBF), AM1 TS (SFS)

- Train MAE: 0.98
- Test MAE: 0.98 ± 0.06
- Lit. 1 MAE: 1.24 ± 0.16
- Lit. 2 MAE: 1.5 ± 0.23
- Lit. 3 MAE: 1.46 ± 0.21
- Train R^2: 0.94
- Test R^2: 0.93
- Lit. 1 R^2: -0.1
- Lit. 2 R^2: -0.08
- Lit. 3 R^2: -0.1

Hyperparameters: KernelRidge(alpha=0.01, gamma=0.01, kernel='rbf')

Features (38): EState (C3), EState (O4), EState (R1), EState (R2), EState (R3), EState (R4), Energy (SPE), HB Acceptors, HB Donors, LogP (R2), LogP (R3), MR (R1), MR (R2), MR (R3), MR (R4), Mulliken (C3), Mulliken (N8), Mulliken (O4), Mulliken (R2), Mulliken (R3), Amide Bonds, PBV (C1), PBV (R4), PEOE (C1), PEOE (C2), PEOE (C3), PEOE (O4), PEOE (R3), PEOE (R4), Pint (C3), SASA (C2), SASA (C3), SASA (C5), SASA (R1), SASA (R2), SASA (R3), Sterimol L (R3), Sterimol L (R4)

KRR(RBF), AM1 All (SFS)

- Train MAE: 0.93
- Test MAE: 0.99 ± 0.06
- Lit. 1 MAE: 0.97 ± 0.14
- Lit. 2 MAE: 1.11 ± 0.16
- Lit. 3 MAE: 1.11 ± 0.16
- Train R^2: 0.94
- Test R^2: 0.93
- Lit. 1 R^2: 0.28
- Lit. 2 R^2: 0.43
- Lit. 3 R^2: 0.36

Hyperparameters: KernelRidge(alpha=0.1, gamma=0.01, kernel='rbf')

Features (42): Barrier, MA MR (C2), MA Mulliken (O4), MA Mulliken (R2), MA Mulliken (R3), MA PEOE (C3), MA PEOE (O4), MA PEOE (R3), MA Pint (C1), MA Pint (C3), MA Pint (R4), MA SASA (C3), MA SASA (R1), MA SASA (R2), MA Sterimol B1 (R1), MA Sterimol B5 (R3), MA Sterimol L (R4), TS EState (C2), TS EState (R1), TS EState (R3), TS EState (R4), TS HB Acceptors, TS HB Donors, TS Hardness, TS LogP (R1), TS LogP (R2), TS LogP (R3), TS MR (R2), TS MR (R3), TS MR (R4), TS Mulliken (C3), TS Mulliken (R3), TS Mulliken (R4), TS Amide Bonds, TS PBV (C5), TS PBV (H6), TS PBV (H7), TS PBV (R3), TS PEOE (R2), TS SASA (C2), TS SASA (N8), TS SASA (R3)

KRR(RBF), PM6 MA (SFS)

- Train MAE: 1.0
- Test MAE: 1.01 ± 0.07
- Lit. 1 MAE: 1.31 ± 0.23
- Lit. 2 MAE: 1.4 ± 0.23
- Lit. 3 MAE: 1.32 ± 0.22
- Train R^2: 0.93
- Test R^2: 0.92
- Lit. 1 R^2: -0.65
- Lit. 2 R^2: -0.0
- Lit. 3 R^2: -0.04

Hyperparameters: KernelRidge(alpha=0.1, kernel='rbf')

Features (42): EState (O4), EState (R1), EState (R2), EState (R3), EState (R4), HB Acceptors, HB Donors, LogP (O4), LogP (R2), LogP (R3), MR (C1), MR (C2), MR (R1), MR (R2), MR (R3), MR (R4), Mulliken (O4), Mulliken (R2), Mulliken (R3), Mulliken (R4), Amide Bonds, PBV (C1), PBV (C2), PEOE (C1), PEOE (C2), PEOE (C3), PEOE (O4), PEOE (R2), PEOE (R3), Pint (C1), Pint (C2), Pint (C3), SASA (C2), SASA (R1), SASA (R2), SASA (R3), Sterimol B1 (R1), Sterimol L (R2), Sterimol B5 (R3), Sterimol L (R3), Sterimol B1 (R4), Sterimol L (R4)

KRR(RBF), PM6 TS (SFS)

- Train MAE: 0.98
- Test MAE: 1.01 ± 0.07
- Lit. 1 MAE: 1.31 ± 0.22
- Lit. 2 MAE: 1.32 ± 0.2
- Lit. 3 MAE: 1.25 ± 0.21
- Train R^2: 0.93
- Test R^2: 0.93
- Lit. 1 R^2: -0.51
- Lit. 2 R^2: 0.16
- Lit. 3 R^2: 0.08

Hyperparameters: KernelRidge(alpha=0.1, kernel='rbf')

Features (30): EState (O4), EState (R1), EState (R2), EState (R3), EState (R4), Energy, HB Acceptors, HB Donors, LogP (R1), LogP (R3), MR (R2), MR (R3), MR (R4), Mulliken (R1), Mulliken (R2), Mulliken (R4), Amide Bonds, PBV (C1), PBV (C5), PEOE (O4), PEOE (R2), PEOE (R3), PEOE (R4), SASA (C2), SASA (C3), SASA (R1), SASA (R2), SASA (R3), Sterimol L (R3), Sterimol L (R4)

KRR(RBF), PM6 All (SFS)

- Train MAE: 0.98
- Test MAE: 1.08 ± 0.07
- Lit. 1 MAE: 1.29 ± 0.18
- Lit. 2 MAE: 1.34 ± 0.18
- Lit. 3 MAE: 1.22 ± 0.18
- Train R^2: 0.94
- Test R^2: 0.92
- Lit. 1 R^2: -0.27
- Lit. 2 R^2: 0.25
- Lit. 3 R^2: 0.23

Hyperparameters: KernelRidge(alpha=0.1, kernel='rbf')

Features (40): MA HOMO, MA MR (C2), MA Mulliken (O4), MA PBV (C1), MA PEOE (C2), MA PEOE (C3), MA PEOE (O4), MA PEOE (R3), MA Pint (C1), MA Pint (R1), MA SASA (R1), MA SASA (R2), MA Sterimol B5 (R1), MA Sterimol B1 (R2), MA Sterimol L (R2), MA Sterimol L (R4), TS EState (O4), TS EState (R2), TS EState (R3), TS EState (R4), TS Energy, TS HB Acceptors, TS HB Donors, TS LogP (O4), TS LogP (R1), TS LogP (R2), TS LogP (R3), TS MR (R2), TS MR (R3), TS MR (R4), TS Mulliken (R4), TS Amide Bonds, TS PBV (C5), TS PBV (R3), TS PEOE (C1), TS PEOE (R2), TS PEOE (R4), TS SASA (C2), TS SASA (R3), TS Vib. Freq.

KRR(RBF), DFT MA (SFS)

- Train MAE: 0.96
- Test MAE: 1.04 ± 0.08
- Lit. 1 MAE: 1.65 ± 0.24
- Lit. 2 MAE: 1.69 ± 0.23
- Lit. 3 MAE: 1.64 ± 0.23
- Train R^2: 0.94
- Test R^2: 0.92
- Lit. 1 R^2: -1.16
- Lit. 2 R^2: -0.24
- Lit. 3 R^2: -0.35

Hyperparameters: KernelRidge(alpha=0.1, kernel='rbf')

Features (36): EState (C2), EState (R1), EState (R2), EState (R3), EState (R4), HB Acceptors, HB Donors, LUMO, LogP (O4), LogP (R2), MR (C1), MR (R2), MR (R3), MR (R4), Mulliken (O4), Mulliken (R2), Mulliken (R3), Amide Bonds, PBV (C1), PBV (C2), PEOE (C1), PEOE (C2), PEOE (O4), PEOE (R2), PEOE (R3), Pint (C1), Pint (C2), Pint (C3), Pint (R4), SASA (C3), SASA (R1), SASA (R3), Sterimol B5 (R3), Sterimol L (R3), Sterimol B1 (R4), Sterimol L (R4)

KRR(RBF), AM1 PM6 All (SFS)

- Train MAE: 0.92
- Test MAE: 1.01 ± 0.06
- Lit. 1 MAE: 0.95 ± 0.12
- Lit. 2 MAE: 1.11 ± 0.16
- Lit. 3 MAE: 1.08 ± 0.15
- Train R^2: 0.94
- Test R^2: 0.93
- Lit. 1 R^2: 0.35
- Lit. 2 R^2: 0.43
- Lit. 3 R^2: 0.42

Hyperparameters: KernelRidge(alpha=0.1, gamma=0.01, kernel='rbf')

Features (47): AM1 Barrier, AM1 MA HOMO, AM1 MA Mulliken (O4), AM1 MA Mulliken (R1), AM1 MA Mulliken (R2), AM1 MA Sterimol L (R4), AM1 TS EState (C2), AM1 TS Energy (SPE), AM1 TS Hardness, AM1 TS Mulliken (C3), AM1 TS Mulliken (R3), AM1 TS Mulliken (R4), AM1 TS PEOE (C1), AM1 TS PEOE (R3), AM1 TS SASA (C2), AM1 TS SASA (C5), AM1 TS SASA (R1), AM1 TS SASA (R2), AM1 TS SASA (R3), AM1 TS Sterimol L (R3), AM1 TS Vib. Freq., PM6 MA MR (C2), PM6 MA Mulliken (O4), PM6 MA PEOE (C3), PM6 MA PEOE (O4), PM6 MA PEOE (R3), PM6 MA Pint (C1), PM6 MA Sterimol B1 (R2), PM6 MA Sterimol L (R2), PM6 TS EState (R2), PM6 TS EState (R3), PM6 TS EState (R4), PM6 TS HB Acceptors, PM6 TS HB Donors, PM6 TS LogP (R1), PM6 TS LogP (R2), PM6 TS LogP (R3), PM6 TS MR (R2), PM6 TS MR (R3), PM6 TS MR (R4), PM6 TS Mulliken (R4), PM6 TS Amide Bonds, PM6 TS PBV (C5), PM6 TS PBV (R3), PM6 TS SASA (C2), PM6 TS SASA (R3), PM6 TS Vib. Freq.

KRR(poly), UFF MA (SFS)

- Train MAE: 0.94
- Test MAE: 1.06 ± 0.07
- Lit. 1 MAE: 2.2 ± 0.3
- Lit. 2 MAE: 2.41 ± 0.32
- Lit. 3 MAE: 2.4 ± 0.32
- Train R^2: 0.94
- Test R^2: 0.92
- Lit. 1 R^2: -2.63
- Lit. 2 R^2: -1.48
- Lit. 3 R^2: -1.75

Hyperparameters: KernelRidge(alpha=10, coef0=2, degree=4, gamma=0.05, kernel='polynomial')

Features (28): EState (C2), EState (R2), EState (R3), EState (R4), HB Donors, LogP (C2), LogP (R2), LogP (R3), LogP (R4), MR (R4), Amide Bonds, PBV (C1), PBV (C2), PBV (C3), PBV (O4), PEOE (C1), PEOE (C2), PEOE (R2), PEOE (R3), Pint (C1), Pint (O4), SASA (C1), SASA (C2), SASA (R3), Sterimol B5 (R1), Sterimol B1 (R3), Sterimol B1 (R4), Vib. Freq.

KRR(poly), AM1 MA (SFS)

- Train MAE: 0.96
- Test MAE: 1.12 ± 0.07
- Lit. 1 MAE: 1.62 ± 0.26
- Lit. 2 MAE: 1.78 ± 0.27
- Lit. 3 MAE: 1.73 ± 0.25
- Train R^2: 0.94
- Test R^2: 0.91
- Lit. 1 R^2: -1.24
- Lit. 2 R^2: -0.5
- Lit. 3 R^2: -0.56

Hyperparameters: KernelRidge(alpha=100, coef0=2, degree=10, gamma=0.01, kernel='polynomial')

Features (21): EState (C2), EState (R2), HB Donors, LogP (R3), MR (R3), MR (R4), Mulliken (O4), Mulliken (R3), Amide Bonds, PBV (C1), PEOE (C2), PEOE (C3), PEOE (R2), PEOE (R3), Sterimol B5 (R2), Sterimol L (R3), Sterimol B1 (R4), Sterimol B5 (R4), TPSA, Vib. Freq., Vib. IR

KRR(poly), AM1 TS (SFS)

- Train MAE: 0.85
- Test MAE: 0.99 ± 0.07
- Lit. 1 MAE: 1.1 ± 0.19
- Lit. 2 MAE: 1.24 ± 0.2
- Lit. 3 MAE: 1.12 ± 0.18
- Train R^2: 0.95
- Test R^2: 0.93
- Lit. 1 R^2: -0.14
- Lit. 2 R^2: 0.21
- Lit. 3 R^2: 0.27

Hyperparameters: KernelRidge(alpha=100, coef0=5, degree=4, gamma=0.05, kernel='polynomial')

Features (42): EState (O4), EState (R3), EState (R4), HB Donors, HOMO, LogP (C2), LogP (O4), LogP (R2), LogP (R3), LogP (R4), MR (R2), MR (R4), Mulliken (N8), Mulliken (R1), Mulliken (R3), Amide Bonds, PBV (C2), PBV (C5), PBV (O4), PBV (R2), PBV (R3), PBV (R4), PEOE (C2), PEOE (C3), PEOE (R1), PEOE (R2), PEOE (R3), Pint (C1), Pint (C2), Pint (C5), Pint (O4), Pint (R1), Pint (R2), Pint (R3), SASA (C2), SASA (C5), SASA (R3), Sterimol B5 (R2), Sterimol B5 (R3), Sterimol B5 (R4), TPSA, Vib. IR

KRR(poly), AM1 All (SFS)

- Train MAE: 0.83
- Test MAE: 0.94 ± 0.06
- Lit. 1 MAE: 0.99 ± 0.15

- Lit. 2 MAE: 1.09 ± 0.16
- Lit. 3 MAE: 1.1 ± 0.16
- Train R^2: 0.95
- Test R^2: 0.94
- Lit. 1 R^2: 0.2
- Lit. 2 R^2: 0.45
- Lit. 3 R^2: 0.36

Hyperparameters: KernelRidge(alpha=10, coef0=4, gamma=0.05, kernel='polynomial')

Features (48): Barrier, MA MR (C1), MA MR (C2), MA Mulliken (O4), MA Mulliken (R3), MA PBV (R1), MA PBV (R2), MA PBV (R4), MA PEOE (R3), MA Pint (C1), MA Pint (R1), MA SASA (C1), MA SASA (O4), MA SASA (R2), MA SASA (R4), MA Sterimol B1 (R2), MA TPSA, TS EState (C2), TS EState (R1), TS Enthalpy, TS HB Donors, TS HOMO, TS LogP (O4), TS LogP (R2), TS LogP (R3), TS LogP (R4), TS MR (R2), TS MR (R3), TS Mulliken (C2), TS Mulliken (C3), TS Mulliken (C5), TS Mulliken (N8), TS Mulliken (R3), TS Mulliken (R4), TS Amide Bonds, TS PBV (C2), TS PBV (C3), TS PBV (C5), TS PBV (N8), TS PBV (O4), TS PBV (R3), TS PEOE (R2), TS SASA (C2), TS SASA (C5), TS SASA (R3), TS Sterimol B5 (R2), TS Sterimol B5 (R3), TS Sterimol B5 (R4)

KRR(poly), PM6 MA (SFS)

- Train MAE: 0.96
- Test MAE: 1.04 ± 0.07
- Lit. 1 MAE: 1.54 ± 0.18
- Lit. 2 MAE: 1.73 ± 0.21
- Lit. 3 MAE: 1.64 ± 0.18
- Train R^2: 0.94
- Test R^2: 0.92
- Lit. 1 R^2: -0.58
- Lit. 2 R^2: -0.21
- Lit. 3 R^2: -0.14

Hyperparameters: KernelRidge(alpha=0.5, degree=7, gamma=0.01, kernel='polynomial')

Features (31): Chem. Pot., EState (C2), EState (R1), EState (R4), HB Donors, LogP (O4), LogP (R2), LogP (R3), MR (C1), MR (C2), MR (R3), MR (R4), Mulliken (O4), Mulliken (R2), Mulliken (R3), Amide Bonds, PBV (C1), PBV (C2), PBV (C3), PEOE (C2), PEOE (O4), PEOE (R2), PEOE (R3), Pint (C1), SASA (C1), SASA (O4), SASA (R3), SASA (R4), Sterimol L (R2), Vib. Freq., ZPE

KRR(poly), PM6 TS (SFS)

- Train MAE: 0.92
- Test MAE: 1.12 ± 0.07
- Lit. 1 MAE: 1.68 ± 0.23
- Lit. 2 MAE: 1.72 ± 0.22
- Lit. 3 MAE: 1.62 ± 0.22
- Train R^2: 0.94
- Test R^2: 0.92
- Lit. 1 R^2: -1.12
- Lit. 2 R^2: -0.22
- Lit. 3 R^2: -0.3

Hyperparameters: KernelRidge(alpha=25, coef0=3, degree=6, gamma=0.01, kernel='polynomial')

Features (47): EState (R1), EState (R3), EState (R4), QH Entropy, HB Acceptors, HB Donors, HOMO, LogP (C2), LogP (O4), LogP (R1), LogP (R2), LogP (R3), LogP (R4), MR (C1), MR (R4), Mulliken (C2), Mulliken (C3), Mulliken (N8), Mulliken (R1), Mulliken (R3), Mulliken (R4), Amide Bonds, PBV (C1), PBV (C2), PBV (C5), PBV (N8), PBV (O4), PBV (R2), PBV (R3), PEOE (R2), PEOE (R3), Pint (C1), Pint (O4), Pint (R1), Pint (R3), SASA (C2), SASA (C3), SASA (C5), SASA (R3), Sterimol B1 (R1), Sterimol L (R1), Sterimol B1 (R3), Sterimol L (R3), Sterimol B1 (R4), TPSA, Vib. Freq., ZPE

KRR(poly), PM6 All (SFS)

- Train MAE: 0.91
- Test MAE: 1.16 ± 0.07
- Lit. 1 MAE: 1.51 ± 0.18
- Lit. 2 MAE: 1.74 ± 0.24
- Lit. 3 MAE: 1.56 ± 0.18
- Train R^2: 0.94
- Test R^2: 0.92
- Lit. 1 R^2: -0.57
- Lit. 2 R^2: -0.31
- Lit. 3 R^2: -0.05

Hyperparameters: KernelRidge(alpha=0.25, degree=5, gamma=0.01, kernel='polynomial')

Features (40): MA HOMO, MA Mulliken (C2), MA Mulliken (O4), MA Mulliken (R3), MA PBV (C1), MA PBV (O4), MA PBV (R1), MA PEOE (C2), MA PEOE (R3), MA Pint (C1), MA Pint (R1), MA Pint (R2), MA Pint (R4), MA SASA (C3), MA Sterimol L (R1), MA Sterimol B1 (R2), MA Sterimol B1 (R3), TS EState (R1), TS EState (R3), TS EState (R4), TS HB Acceptors, TS HB Donors, TS LogP (C2), TS LogP (R3), TS MR (C2), TS MR (R4), TS Mulliken (C3), TS Mulliken (N8), TS Mulliken (R4), TS Amide Bonds, TS PBV (C5), TS PBV (N8), TS PBV (R3), TS PEOE (C1), TS PEOE (R2), TS SASA (C2), TS SASA (O4), TS SASA (R3), TS Sterimol B1 (R1), TS Vib. Freq.

KRR(poly), DFT MA (SFS)

- Train MAE: 0.92
- Test MAE: 1.04 ± 0.07
- Lit. 1 MAE: 1.66 ± 0.19
- Lit. 2 MAE: 1.8 ± 0.2
- Lit. 3 MAE: 1.69 ± 0.18
- Train R^2: 0.94
- Test R^2: 0.92
- Lit. 1 R^2: -0.79
- Lit. 2 R^2: -0.23
- Lit. 3 R^2: -0.18

Hyperparameters: KernelRidge(alpha=50, coef0=3, degree=4, gamma=0.05, kernel='polynomial')

Features (42): EState (C1), EState (R1), EState (R2), EState (R3), EState (R4), QH Entropy, HB Donors, LUMO, LogP (O4), LogP (R2), LogP (R3), LogP (R4), MR (C1), MR (R4), Mulliken (C2), Mulliken (O4), Mulliken (R3), Amide Bonds, PBV (C1), PBV (C2), PBV (C3), PBV (R4), PEOE (C1), PEOE (C2), PEOE (R2), PEOE (R3), Pint (O4), Pint (R2), Surface Area, SASA (C1), SASA (C2), SASA (C3), SASA (O4), SASA (R2), SASA (R3), Sterimol B1 (R1), Sterimol B5 (R1), Sterimol B5 (R2), Sterimol L (R2), Sterimol B1 (R3), Sterimol B1 (R4), Vib. IR

KRR(poly), AM1 PM6 All (SFS)

- Train MAE: 0.82
- Test MAE: 0.88 ± 0.06
- Lit. 1 MAE: 1.3 ± 0.16
- Lit. 2 MAE: 1.46 ± 0.18
- Lit. 3 MAE: 1.43 ± 0.17
- Train R^2: 0.96
- Test R^2: 0.94
- Lit. 1 R^2: -0.16
- Lit. 2 R^2: 0.13
- Lit. 3 R^2: 0.09

Hyperparameters: KernelRidge(alpha=2, coef0=2, degree=4, gamma=0.01, kernel='polynomial')

Features (76): AM1 Barrier, AM1 MA Mulliken (O4), AM1 MA Mulliken (R3), AM1 MA PBV (R4), AM1 MA Pint (R4), AM1 MA SASA (C1), AM1 MA SASA (C3), AM1 MA SASA (O4), AM1 MA Sterimol B5 (R3), AM1 MA Sterimol B1 (R4), AM1 TS Chem. Pot., AM1 TS EState (C3), AM1 TS Electrophilicity, AM1 TS HOMO, AM1 TS Hardness, AM1 TS LUMO, AM1 TS MR (C1), AM1 TS Mulliken (C2), AM1 TS Mulliken (C3), AM1 TS Mulliken (C5), AM1 TS Mulliken (H7), AM1 TS Mulliken (N8), AM1 TS Mulliken (R3), AM1 TS Mulliken (R4), AM1 TS PBV (C1), AM1 TS PBV (C2), AM1 TS PBV (C3), AM1 TS PBV (C5), AM1 TS PBV (N8), AM1 TS PBV (R3), AM1 TS PEOE (R2), AM1 TS PEOE (R3), AM1 TS Pint (O4), AM1 TS SASA (C2), AM1 TS SASA (C5), AM1 TS SASA (R2), AM1 TS Sterimol B5 (R2), AM1 TS Sterimol B5 (R3), AM1 TS Sterimol B5 (R4), AM1 TS Vib. Freq., AM1 TS Vib. IR, AM1 TS ZPE, PM6 MA MR (C1), PM6 MA MR (C2), PM6 MA Mulliken (R2), PM6 MA Mulliken (R3), PM6 MA PEOE (R3), PM6 MA Pint (C2), PM6 MA Pint (C3), PM6 MA Pint (R2), PM6 MA Pint (R3), PM6 MA Pint (R4), PM6 MA SASA (O4), PM6 MA SASA (R2), PM6 MA Softness, PM6 MA Sterimol L (R2), PM6 MA Sterimol L (R4), PM6 MA TPSA, PM6 TS EState (R1), PM6 TS EState (R3), PM6 TS HB Donors, PM6 TS LogP (R1), PM6 TS LogP (R3), PM6 TS LogP (R4), PM6 TS Mulliken (C1), PM6 TS Mulliken (C2), PM6 TS Amide Bonds, PM6 TS PBV (N8), PM6 TS PBV (R1), PM6 TS PBV (R3), PM6 TS PBV (R4), PM6 TS PEOE (R2), PM6 TS SASA (R3), PM6 TS Sterimol B5 (R2), PM6 TS Sterimol B1 (R4), PM6 TS Sterimol B5 (R4)

GPR, UFF MA (importances-0.5*mean)

- Train MAE: 0.99
- Test MAE: 1.0 ± 0.08
- Lit. 1 MAE: 1.2 ± 0.18
- Lit. 2 MAE: 1.42 ± 0.22
- Lit. 3 MAE: 1.4 ± 0.21
- Train R^2: 0.93
- Test R^2: 0.92
- Lit. 1 R^2: -0.15
- Lit. 2 R^2: 0.0
- Lit. 3 R^2: -0.06

Hyperparameters: GaussianProcessRegressor(alpha=0.1, kernel=1**2 * Matern(length_scale=1, nu=1.5))

Features (66): EState (C1), EState (C2), EState (C3), EState (O4), EState (R1), EState (R2), EState (R3), EState (R4), Energy, QH Entropy, HB Acceptors, HB Donors, LogP (C2), LogP (R2), LogP (R3), LogP (R4), MR (C1), MR (C2), MR (R1), MR (R2), MR (R3), MR (R4), Amide Bonds, PBV (C1), PBV (C2), PBV (C3), PBV (O4), PBV (R1), PBV (R2), PBV (R4), PEOE (C1), PEOE (C2), PEOE (R1), PEOE (R2), PEOE (R3), PEOE (R4), Pint (C1), Pint (C2), Pint (C3), Pint (O4), Pint (R1), Pint (R2), Pint (R3), Pint (R4), Surface Area, SASA (C1), SASA (C2), SASA (C3), SASA (O4), SASA (R1), SASA (R2), SASA (R3), SASA (R4), Sterimol B1 (R1), Sterimol B5 (R1), Sterimol B1 (R2), Sterimol B5 (R2), Sterimol L (R2), Sterimol B1 (R3), Sterimol B5 (R3), Sterimol L (R3), Sterimol B1 (R4), Sterimol B5 (R4), Sterimol L (R4), TPSA, Vib. Freq.

GPR, AM1 MA (importances-0.5*mean)

- Train MAE: 0.99
- Test MAE: 1.02 ± 0.08
- Lit. 1 MAE: 1.21 ± 0.18
- Lit. 2 MAE: 1.39 ± 0.21
- Lit. 3 MAE: 1.34 ± 0.19
- Train R^2: 0.94
- Test R^2: 0.92
- Lit. 1 R^2: -0.14
- Lit. 2 R^2: 0.1
- Lit. 3 R^2: 0.1

Hyperparameters: GaussianProcessRegressor(alpha=0.1, kernel=1**2 * Matern(length_scale=1, nu=1.5))

Features (72): EState (C1), EState (C2), EState (C3), EState (O4), EState (R1), EState (R2), EState (R3), EState (R4), Energy, Enthalpy, QH Entropy, HB Acceptors, HB Donors, HOMO, Hardness, LUMO, LogP (R2), LogP (R3), LogP (R4), MR (C2), MR (R1), MR (R2), MR (R3), MR (R4), Mulliken (C1), Mulliken (C2), Mulliken (C3), Mulliken (O4), Mulliken (R1), Mulliken (R2), Mulliken (R3), Mulliken (R4), Amide Bonds, PBV (C1), PBV (C2), PBV (C3), PBV (R1), PBV (R2), PBV (R3), PBV (R4), PEOE (C1), PEOE (C2), PEOE (R1), PEOE (R2), PEOE (R3), PEOE (R4), Pint (C1), Pint (C2), Pint (C3), Pint (O4), Pint (R1), Pint (R3), Pint (R4), SASA (C1), SASA (C2), SASA (O4), SASA (R3), SASA (R4), Sterimol B1 (R1), Sterimol B5 (R1), Sterimol B1 (R2), Sterimol B5 (R2), Sterimol B1 (R3), Sterimol B5 (R3), Sterimol L (R3), Sterimol B1 (R4), Sterimol B5 (R4), Sterimol L (R4), TPSA, Vib. Freq., Vib. IR, ZPE

GPR, AM1 TS (importances-0.5*mean)

- Train MAE: 0.94
- Test MAE: 0.99 ± 0.08
- Lit. 1 MAE: 1.21 ± 0.2
- Lit. 2 MAE: 1.37 ± 0.22
- Lit. 3 MAE: 1.32 ± 0.2
- Train R^2: 0.94
- Test R^2: 0.92
- Lit. 1 R^2: -0.27
- Lit. 2 R^2: 0.07
- Lit. 3 R^2: 0.06

Hyperparameters: GaussianProcessRegressor(alpha=0.1, kernel=1**2 * Matern(length_scale=1, nu=1.5))

Features (87): Chem. Pot., Distance, EState (C1), EState (C2), EState (C3), EState (O4), EState (R3), EState (R4), Energy (SPE), Enthalpy, HB Acceptors, HB Donors, HOMO, Hardness, LogP (C1), LogP (O4), LogP (R1), LogP (R2), LogP (R3), LogP (R4), MR (R1), MR (R2), MR (R3), MR (R4), Mulliken (C1), Mulliken (C2), Mulliken (C5), Mulliken (H6), Mulliken (H7), Mulliken (N8), Mulliken (O10), Mulliken (O4), Mulliken (O9), Mulliken (R3), Mulliken (R4), Amide Bonds, PBV (C1), PBV (C2), PBV (C3), PBV (C5), PBV (H6), PBV (H7), PBV (N8), PBV (O4), PBV (R2), PBV (R3), PEOE (C1), PEOE (C2), PEOE (R2), PEOE (R3), PEOE (R4), Pint (C1), Pint (C2), Pint (C3), Pint (C5), Pint (H6), Pint (N8), Pint (O10), Pint (O4), Pint (O9), Pint (R1), Pint (R2), Pint (R3), Pint (R4), Surface Vol., SASA (C2), SASA (C3), SASA (C5), SASA (H7), SASA (N8), SASA (O10), SASA (R3), Sterimol B5 (R1), Sterimol L (R1), Sterimol B1 (R2), Sterimol B5 (R2), Sterimol L (R2), Sterimol B1 (R3), Sterimol B5 (R3), Sterimol L (R3), Sterimol B1 (R4), Sterimol B5 (R4), Sterimol L (R4), TPSA, Vib. Freq., Vib. IR, ZPE

GPR, AM1 All (importances-0.5*mean)

- Train MAE: 0.92
- Test MAE: 0.96 ± 0.07
- Lit. 1 MAE: 0.92 ± 0.18
- Lit. 2 MAE: 1.12 ± 0.22
- Lit. 3 MAE: 1.08 ± 0.2
- Train R^2: 0.94
- Test R^2: 0.93
- Lit. 1 R^2: 0.1
- Lit. 2 R^2: 0.24
- Lit. 3 R^2: 0.24

Hyperparameters: GaussianProcessRegressor(alpha=0.1, kernel=1**2 * Matern(length_scale=1, nu=1.5))

Features (101): Barrier, MA Chem. Pot., MA EState (C1), MA Electrophilicity, MA QH Entropy, MA HOMO, MA Mulliken (C1), MA Mulliken (C2), MA Mulliken (O4), MA Mulliken (R2), MA Mulliken (R3), MA PBV (R1), MA PBV (R2), MA PBV (R4), MA PEOE (R3), MA Pint (C1), MA Pint (C2), MA Pint (R3), MA Pint (R4), MA SASA (C1), MA SASA (O4), MA SASA (R4), MA Sterimol

B5 (R1), MA Sterimol L (R1), MA Sterimol B1 (R2), MA Sterimol B5 (R3), MA Sterimol B1 (R4), MA Sterimol B5 (R4), MA Sterimol L (R4), MA Vib. Freq., MA Vib. IR, TS Chem. Pot., TS Distance, TS EState (C1), TS EState (C2), TS EState (C3), TS EState (O4), TS EState (R1), TS EState (R2), TS EState (R3), TS EState (R4), TS Energy (SPE), TS Enthalpy, TS HB Acceptors, TS HB Donors, TS HOMO, TS Hardness, TS LogP (C1), TS LogP (R2), TS LogP (R3), TS LogP (R4), TS MR (R1), TS MR (R2), TS MR (R3), TS MR (R4), TS Mulliken (C1), TS Mulliken (C2), TS Mulliken (C3), TS Mulliken (C5), TS Mulliken (H6), TS Mulliken (H7), TS Mulliken (N8), TS Mulliken (O10), TS Mulliken (O4), TS Mulliken (O9), TS Mulliken (R3), TS Mulliken (R4), TS Amide Bonds, TS PBV (C1), TS PBV (C2), TS PBV (C3), TS PBV (C5), TS PBV (H6), TS PBV (H7), TS PBV (O4), TS PBV (R3), TS PEOE (C1), TS PEOE (C2), TS PEOE (R2), TS PEOE (R4), TS Pint (C5), TS Pint (H6), TS Pint (H7), TS Pint (N8), TS Pint (O10), TS Pint (O4), TS Pint (O9), TS SASA (C2), TS SASA (C5), TS SASA (H6), TS SASA (H7), TS SASA (N8), TS SASA (O4), TS Sterimol B5 (R2), TS Sterimol B1 (R3), TS Sterimol B5 (R3), TS Sterimol L (R3), TS Sterimol B1 (R4), TS Sterimol B5 (R4), TS Vib. Freq., TS Vib. IR

GPR, AM1 All (importances-0.5*mean, random state=1)

- Train MAE: 0.92
- Test MAE: 0.87 ± 0.06
- Lit. 1 MAE: 0.94 ± 0.17
- Lit. 2 MAE: 1.1 ± 0.2
- Lit. 3 MAE: 1.04 ± 0.18
- Train R^2: 0.94
- Test R^2: 0.95
- Lit. 1 R^2: 0.14
- Lit. 2 R^2: 0.33
- Lit. 3 R^2: 0.35

Hyperparameters: GaussianProcessRegressor(alpha=0.1, kernel=1**2 * Matern(length_scale=1, nu=1.5))

Features (112): Barrier, MA Chem. Pot., MA EState (C1), MA Electrophilicity, MA QH Entropy, MA HOMO, MA MR (C2), MA Mulliken (C1), MA Mulliken (C2), MA Mulliken (O4), MA Mulliken (R1), MA Mulliken (R2), MA Mulliken (R3), MA PBV (R1), MA PBV (R2), MA PBV (R4), MA PEOE (R3), MA Pint (C1), MA Pint (C2), MA Pint (R1), MA Pint (R2), MA Pint (R3), MA Surface Area, MA SASA (C3), MA SASA (R2), MA SASA (R4), MA Sterimol B1 (R1), MA Sterimol B5 (R1), MA Sterimol L (R1), MA Sterimol L (R2), MA Sterimol B1 (R3), MA Sterimol B5 (R3), MA Sterimol B1 (R4), MA Sterimol B5 (R4), MA Sterimol L (R4), MA TPSA, MA Vib. Freq., MA Vib. IR, TS Distance, TS EState (C1), TS EState (C3), TS EState (O4), TS EState (O9), TS EState (R1), TS EState (R2), TS EState (R3), TS EState (R4), TS Electrophilicity, TS Energy (SPE), TS Enthalpy, TS HB Acceptors, TS HB Donors, TS HOMO, TS LogP (C2), TS LogP (O4), TS LogP (R1), TS MR (C1), TS MR (C2), TS MR (R1), TS MR (R2), TS MR (R3), TS MR (R4), TS Mulliken (C1), TS Mulliken (C2), TS Mulliken (C3), TS Mulliken (C5), TS Mulliken (H6), TS Mulliken (H7), TS Mulliken (N8), TS Mulliken (O10), TS Mulliken (O4), TS Mulliken (O9), TS Mulliken (R3), TS Mulliken (R4), TS Amide Bonds, TS PBV (C1), TS PBV (C2), TS PBV (C3), TS PBV (C5), TS PBV (H6), TS PBV (N8), TS PBV (O4), TS PBV (O9), TS PBV (R3), TS PEOE (C1), TS PEOE (C2), TS PEOE (R1), TS PEOE (R2), TS PEOE (R4), TS Pint (C5), TS Pint (H6), TS Pint (H7), TS Pint (N8), TS Pint (O10), TS Pint (O4), TS Pint (O9), TS SASA (C2), TS SASA (H6), TS SASA (H7), TS SASA (O10), TS SASA (O9), TS SASA (R3), TS Sterimol B1 (R1), TS Sterimol B5 (R2), TS Sterimol B1 (R3), TS Sterimol B5 (R3), TS Sterimol L (R3), TS Sterimol B1 (R4), TS Sterimol B5 (R4), TS Vib. Freq., TS Vib. IR, TS ZPE

GPR, AM1 All (importances-0.5*mean, random state=2)

- Train MAE: 0.94
- Test MAE: 0.85 ± 0.05
- Lit. 1 MAE: 0.88 ± 0.18
- Lit. 2 MAE: 1.06 ± 0.21
- Lit. 3 MAE: 1.01 ± 0.19
- Train R^2: 0.94
- Test R^2: 0.95
- Lit. 1 R^2: 0.16
- Lit. 2 R^2: 0.31
- Lit. 3 R^2: 0.32

Hyperparameters: GaussianProcessRegressor(alpha=1, kernel=1**2 * Matern(length_scale=1, nu=1.5))

Features (105): Barrier, MA Chem. Pot., MA EState (C1), MA Electrophilicity, MA QH Entropy, MA HOMO, MA Hardness, MA LUMO, MA MR (C2), MA Mulliken (C1), MA Mulliken (C2), MA Mulliken (O4), MA Mulliken (R2), MA Mulliken (R3), MA PBV (R1), MA PBV (R2), MA PBV (R4), MA PEOE (R3), MA Pint (C1), MA Pint (C2), MA Pint (R2), MA Pint (R3), MA Surface Area, MA SASA (C3), MA SASA (R1), MA SASA (R2), MA Sterimol B1 (R1), MA Sterimol B5 (R1), MA Sterimol L (R1), MA Sterimol L (R2), MA Sterimol B1 (R3), MA Sterimol B5 (R3), MA Sterimol B1 (R4), MA Sterimol B5 (R4), MA TPSA, MA Vib. Freq., MA Vib. IR, TS Distance, TS EState (C1), TS EState (C3), TS EState (O4), TS EState (O9), TS EState (R1), TS EState (R2), TS EState (R4), TS Electrophilicity, TS Energy (SPE), TS Enthalpy, TS HB Acceptors, TS HB Donors, TS LogP (O4), TS LogP (R1), TS LogP (R2), TS MR (C1), TS MR (C2), TS MR (R1), TS MR (R2), TS MR (R3), TS MR (R4), TS Mulliken (C1), TS Mulliken (C2), TS Mulliken (C3), TS Mulliken (C5), TS Mulliken (H6), TS Mulliken (H7), TS Mulliken (N8), TS Mulliken (O10), TS Mulliken (O4), TS Mulliken (O9), TS Mulliken (R3), TS Mulliken (R4), TS Amide Bonds, TS PBV (C1), TS PBV (C2), TS PBV (C3), TS PBV (C5), TS PBV (H6), TS PBV (N8), TS PBV (O4), TS PBV (O9), TS PBV (R3), TS PEOE (C2), TS PEOE (R1), TS PEOE (R2), TS PEOE (R4), TS Pint (C5), TS Pint (H6), TS Pint (H7), TS Pint (N8), TS Pint (O10), TS Pint (O4), TS Pint (O9), TS SASA (C2), TS SASA (H6), TS SASA (H7), TS SASA (O10), TS SASA (O9), TS Sterimol B1 (R1), TS Sterimol B5 (R2), TS Sterimol B1 (R3), TS Sterimol B5 (R3), TS Sterimol L (R3), TS Sterimol B1 (R4), TS Vib. Freq., TS Vib. IR

GPR, AM1 All (importances-0.5*mean, random state=3)

- Train MAE: 0.96
- Test MAE: 0.88 ± 0.06
- Lit. 1 MAE: 0.95 ± 0.18
- Lit. 2 MAE: 1.14 ± 0.21
- Lit. 3 MAE: 1.09 ± 0.19
- Train R^2: 0.94
- Test R^2: 0.95
- Lit. 1 R^2: 0.11
- Lit. 2 R^2: 0.25
- Lit. 3 R^2: 0.26

Hyperparameters: GaussianProcessRegressor(alpha=1, kernel=1**2 * Matern(length_scale=1, nu=1.5))

Features (106): Barrier, MA EState (C1), MA QH Entropy, MA HOMO, MA Hardness, MA MR (C1), MA MR (C2), MA Mulliken (C1), MA Mulliken (C2), MA Mulliken (O4), MA Mulliken (R3), MA PBV (R1), MA PBV (R2), MA PBV (R4), MA PEOE (R3), MA Pint (C1), MA Pint (C2), MA Pint (C3), MA Pint (R2), MA Pint (R4), MA SASA (C1), MA SASA (C3), MA SASA (O4), MA SASA (R4), MA Sterimol B5 (R1), MA Sterimol L (R1), MA Sterimol B1 (R2), MA Sterimol B1 (R3), MA Sterimol B5 (R3), MA Sterimol B1 (R4), MA Sterimol B5 (R4), MA Sterimol L (R4), MA Vib. IR, TS Chem. Pot., TS Distance, TS EState (C2), TS EState (C3), TS EState (O4), TS EState (R1), TS EState (R2), TS EState (R3), TS EState (R4), TS Energy (SPE), TS Enthalpy, TS HB Acceptors, TS HB Donors, TS HOMO, TS Hardness, TS LogP (R2), TS LogP (R3), TS LogP (R4), TS MR (R1), TS MR (R2), TS MR (R3), TS MR (R4), TS Mulliken (C1), TS Mulliken (C2), TS Mulliken (C3), TS Mulliken (C5), TS Mulliken (H6), TS Mulliken (H7), TS Mulliken (N8), TS Mulliken (O10), TS Mulliken (O4), TS Mulliken (O9), TS Mulliken (R3), TS Mulliken (R4), TS Amide Bonds, TS PBV (C1), TS PBV (C2), TS PBV (C3), TS PBV (C5), TS PBV (H6), TS PBV (H7), TS PBV (N8), TS PBV (O10),

TS PBV (O4), TS PBV (R3), TS PEOE (C1), TS PEOE (C2), TS PEOE (R2), TS PEOE (R4), TS Pint (C5), TS Pint (H6), TS Pint (H7), TS Pint (N8), TS Pint (O10), TS Pint (O4), TS Pint (O9), TS SASA (C2), TS SASA (C5), TS SASA (H7), TS SASA (N8), TS SASA (O4), TS SASA (O9), TS SASA (R3), TS Sterimol B1 (R1), TS Sterimol B5 (R2), TS Sterimol B1 (R3), TS Sterimol B5 (R3), TS Sterimol L (R3), TS Sterimol B1 (R4), TS Sterimol B5 (R4), TS Vib. Freq., TS Vib. IR, TS ZPE

GPR, AM1 All (importances-0.5*mean, random state=4)

- Train MAE: 0.92
- Test MAE: 0.87 ± 0.06
- Lit. 1 MAE: 1.12 ± 0.19
- Lit. 2 MAE: 1.25 ± 0.2
- Lit. 3 MAE: 1.23 ± 0.19
- Train R^2: 0.94
- Test R^2: 0.95
- Lit. 1 R^2: -0.12
- Lit. 2 R^2: 0.24
- Lit. 3 R^2: 0.16

Hyperparameters: GaussianProcessRegressor(alpha=0.1, kernel=1**2 * Matern(length_scale=1, nu=1.5))

Features (105): Barrier, MA EState (C1), MA Electrophilicity, MA QH Entropy, MA MR (C2), MA Mulliken (C1), MA Mulliken (C2), MA Mulliken (O4), MA Mulliken (R2), MA Mulliken (R3), MA PBV (R1), MA PBV (R2), MA PBV (R4), MA PEOE (R3), MA Pint (C1), MA Pint (C2), MA Pint (R2), MA Pint (R3), MA Surface Area, MA SASA (C1), MA SASA (C3), MA SASA (R2), MA SASA (R4), MA Sterimol B1 (R1), MA Sterimol L (R1), MA Sterimol L (R2), MA Sterimol B1 (R3), MA Sterimol B5 (R3), MA Sterimol B1 (R4), MA Sterimol B5 (R4), MA Sterimol L (R4), MA TPSA, MA Vib. Freq., MA Vib. IR, TS Distance, TS EState (C1), TS EState (C3), TS EState (O4), TS EState (O9), TS EState (R1), TS EState (R2), TS EState (R4), TS Electrophilicity, TS Energy (SPE), TS Enthalpy, TS HB Acceptors, TS HB Donors, TS LogP (C1), TS LogP (C2), TS LogP (O4), TS LogP (R4), TS MR (C1), TS MR (C2), TS MR (R1), TS MR (R2), TS MR (R3), TS MR (R4), TS Mulliken (C1), TS Mulliken (C2), TS Mulliken (C3), TS Mulliken (C5), TS Mulliken (H6), TS Mulliken (H7), TS Mulliken (N8), TS Mulliken (O10), TS Mulliken (O4), TS Mulliken (O9), TS Mulliken (R3), TS Mulliken (R4), TS Amide Bonds, TS PBV (C1), TS PBV (C3), TS PBV (C5), TS PBV (H6), TS PBV (H7), TS PBV (O10), TS PBV (O4), TS PBV (O9), TS PEOE (C1), TS PEOE (C2), TS PEOE (R1), TS PEOE (R2), TS PEOE (R4), TS Pint (C5), TS Pint (H6), TS Pint (H7), TS Pint (N8), TS Pint (O10), TS Pint (O4), TS Pint (O9), TS SASA (C2), TS SASA (H6), TS SASA (H7), TS SASA (N8), TS SASA (O10), TS SASA (O9), TS Sterimol B1 (R1), TS Sterimol B5 (R2), TS Sterimol B1 (R3), TS Sterimol B5 (R3), TS Sterimol L (R3), TS Sterimol B1 (R4), TS Vib. Freq., TS Vib. IR, TS ZPE

GPR, AM1 All (importances-0.5*mean, random state=5)

- Train MAE: 0.94
- Test MAE: 0.92 ± 0.07
- Lit. 1 MAE: 1.08 ± 0.18
- Lit. 2 MAE: 1.21 ± 0.2
- Lit. 3 MAE: 1.21 ± 0.2
- Train R^2: 0.94
- Test R^2: 0.94
- Lit. 1 R^2: -0.06
- Lit. 2 R^2: 0.25
- Lit. 3 R^2: 0.16

Hyperparameters: GaussianProcessRegressor(alpha=0.1, kernel=1**2 * Matern(length_scale=1, nu=1.5))

Features (104): Barrier, MA Chem. Pot., MA EState (C1), MA Electrophilicity, MA QH Entropy, MA HOMO, MA LUMO, MA MR (C2), MA Mulliken (C1), MA Mulliken (C2), MA Mulliken (O4), MA Mulliken (R2), MA Mulliken (R3), MA PBV (R1), MA PBV (R2), MA PBV (R4), MA PEOE (R3), MA Pint (C1), MA Pint (C2), MA Pint (C3), MA Pint (R4), MA SASA (C1), MA SASA (O4), MA Sterimol B1 (R1), MA Sterimol B1 (R2), MA Sterimol B1 (R3), MA Sterimol B5 (R3), MA Sterimol B1 (R4), MA Sterimol B5 (R4), MA Sterimol L (R4), MA TPSA, MA Vib. Freq., MA Vib. IR, TS Chem. Pot., TS EState (C1), TS EState (C2), TS EState (N8), TS EState (O9), TS EState (R1), TS EState (R2), TS EState (R3), TS Electrophilicity, TS Energy (SPE), TS Enthalpy, TS HB Acceptors, TS HB Donors, TS HOMO, TS Hardness, TS LogP (O4), TS LogP (R1), TS LogP (R2), TS MR (R1), TS MR (R2), TS MR (R3), TS MR (R4), TS Mulliken (C1), TS Mulliken (C2), TS Mulliken (C3), TS Mulliken (C5), TS Mulliken (H6), TS Mulliken (H7), TS Mulliken (O10), TS Mulliken (O4), TS Mulliken (O9), TS Mulliken (R3), TS Mulliken (R4), TS Amide Bonds, TS PBV (C1), TS PBV (C2), TS PBV (C3), TS PBV (C5), TS PBV (H6), TS PBV (H7), TS PBV (O10), TS PBV (O9), TS PBV (R3), TS PEOE (C1), TS PEOE (R1), TS PEOE (R2), TS PEOE (R4), TS Pint (C5), TS Pint (H6), TS Pint (H7), TS Pint (N8), TS Pint (O10), TS Pint (O4), TS Pint (O9), TS SASA (C2), TS SASA (C5), TS SASA (H6), TS SASA (H7), TS SASA (N8), TS SASA (O10), TS SASA (O4), TS SASA (O9), TS Sterimol B1 (R1), TS Sterimol B5 (R2), TS Sterimol B1 (R3), TS Sterimol B5 (R3), TS Sterimol L (R3), TS Sterimol B1 (R4), TS Vib. Freq., TS Vib. IR, TS ZPE

GPR, PM6 MA (importances-0.5*mean)

- Train MAE: 1.0
- Test MAE: 1.05 ± 0.08
- Lit. 1 MAE: 1.06 ± 0.15
- Lit. 2 MAE: 1.29 ± 0.21
- Lit. 3 MAE: 1.22 ± 0.18
- Train R^2: 0.93
- Test R^2: 0.91
- Lit. 1 R^2: 0.12
- Lit. 2 R^2: 0.14
- Lit. 3 R^2: 0.21

Hyperparameters: GaussianProcessRegressor(alpha=0.1, kernel=1**2 * Matern(length_scale=1, nu=1.5))

Features (78): Chem. Pot., EState (C1), EState (C2), EState (C3), EState (O4), EState (R1), EState (R2), EState (R3), EState (R4), Electrophilicity, Energy, Enthalpy, QH Entropy, HB Acceptors, HB Donors, HOMO, LUMO, LogP (R2), LogP (R3), LogP (R4), MR (C1), MR (C2), MR (R1), MR (R2), MR (R3), MR (R4), Mulliken (C1), Mulliken (C2), Mulliken (C3), Mulliken (O4), Mulliken (R1), Mulliken (R2), Mulliken (R3), Mulliken (R4), Amide Bonds, PBV (C1), PBV (C2), PBV (C3), PBV (O4), PBV (R1), PBV (R2), PBV (R4), PEOE (C1), PEOE (C2), PEOE (R1), PEOE (R2), PEOE (R3), PEOE (R4), Pint (C1), Pint (C2), Pint (C3), Pint (O4), Pint (R1), Pint (R2), Pint (R3), Pint (R4), SASA (C1), SASA (C2), SASA (C3), SASA (O4), SASA (R2), SASA (R3), SASA (R4), Softness, Sterimol B1 (R1), Sterimol B5 (R1), Sterimol B1 (R2), Sterimol B5 (R2), Sterimol L (R2), Sterimol B1 (R3), Sterimol L (R3), Sterimol B1 (R4), Sterimol B5 (R4), Sterimol L (R4), TPSA, Vib. Freq., Vib. IR, ZPE

GPR, PM6 TS (importances-0.5*mean)

- Train MAE: 1.04
- Test MAE: 1.02 ± 0.07
- Lit. 1 MAE: 1.3 ± 0.16
- Lit. 2 MAE: 1.4 ± 0.17
- Lit. 3 MAE: 1.38 ± 0.16
- Train R^2: 0.92
- Test R^2: 0.93
- Lit. 1 R^2: -0.15
- Lit. 2 R^2: 0.23
- Lit. 3 R^2: 0.17

Hyperparameters: GaussianProcessRegressor(alpha=0.1, kernel=1**2 * Matern(length_scale=1, nu=1.5))

Features (85): Chem. Pot., Distance, EState (C2), EState (C3), EState (O4), EState (R1), EState (R2), EState (R3), EState (R4), Energy, Enthalpy, HB Acceptors, HB Donors, HOMO, LUMO, LogP (R2), LogP (R3), LogP (R4), MR (C1), MR (R1), MR (R2), MR (R3), MR (R4), Mulliken (C1), Mulliken (C2), Mulliken (C5), Mulliken (H6), Mulliken (H7), Mulliken (N8), Mulliken (O4), Mulliken (R3), Mulliken (R4), Amide Bonds, PBV (C1), PBV (C2), PBV (C3), PBV (C5), PBV (H6), PBV (H7), PBV (N8), PBV (O10), PBV (O9), PBV (R1), PBV (R2), PBV (R3), PEOE (C1), PEOE (R1), PEOE (R2), PEOE (R3), PEOE (R4), Pint (C1), Pint (C2), Pint (C3), Pint (C5), Pint (H7), Pint (N8), Pint (O10), Pint (O9), Pint (R2), Pint (R3), Pint (R4), Surface Vol., SASA (C2), SASA (C3), SASA (C5), SASA (H6), SASA (H7), SASA (N8), SASA (O10), SASA (O9), SASA (R3), SASA (R4), Softness, Sterimol B1 (R1), Sterimol B5 (R1), Sterimol B5 (R2), Sterimol L (R2), Sterimol B1 (R3), Sterimol L (R3), Sterimol B1 (R4), Sterimol B5 (R4), Sterimol L (R4), Vib. Freq., Vib. IR, ZPE

GPR, PM6 All (importances-0.5*mean)

- Train MAE: 1.04
- Test MAE: 1.01 ± 0.07
- Lit. 1 MAE: 1.2 ± 0.18
- Lit. 2 MAE: 1.34 ± 0.19
- Lit. 3 MAE: 1.27 ± 0.18
- Train R^2: 0.93
- Test R^2: 0.93
- Lit. 1 R^2: -0.15
- Lit. 2 R^2: 0.18
- Lit. 3 R^2: 0.2

Hyperparameters: GaussianProcessRegressor(alpha=0.1, kernel=1**2 * Matern(length_scale=1, nu=1.5))

Features (105): Barrier, MA EState (C1), MA QH Entropy, MA MR (C2), MA Mulliken (C2), MA Mulliken (O4), MA Mulliken (R1), MA Mulliken (R2), MA Mulliken (R3), MA PBV (C1), MA PBV (R1), MA PEOE (C2), MA PEOE (R3), MA Pint (C1), MA Pint (C2), MA Pint (C3), MA Pint (R1), MA Pint (R3), MA Pint (R4), MA SASA (C1), MA SASA (O4), MA SASA (R1), MA SASA (R2), MA SASA (R4), MA Sterimol B1 (R1), MA Sterimol B5 (R1), MA Sterimol B1 (R2), MA Sterimol B1 (R3), MA Sterimol B5 (R3), MA Sterimol B1 (R4), MA Sterimol B5 (R4), MA Sterimol L (R4), MA TPSA, MA Vib. Freq., MA Vib. IR, TS Chem. Pot., TS Distance, TS EState (C2), TS EState (C3), TS EState (O4), TS EState (R1), TS EState (R2), TS EState (R3), TS EState (R4), TS Energy, TS Enthalpy, TS HB Acceptors, TS HB Donors, TS HOMO, TS LUMO, TS LogP (R1), TS LogP (R2), TS LogP (R3), TS LogP (R4), TS MR (C1), TS MR (R1), TS MR (R3), TS MR (R4), TS Mulliken (C1), TS Mulliken (C2), TS Mulliken (C5), TS Mulliken (H6), TS Mulliken (H7), TS Mulliken (N8), TS Mulliken (O4), TS Mulliken (O9), TS Mulliken (R4), TS Amide Bonds, TS PBV (C2), TS PBV (C3), TS PBV (C5), TS PBV (H6), TS PBV (H7), TS PBV (N8), TS PBV (O10), TS PBV (R3), TS PBV (R4), TS PEOE (C1), TS PEOE (R2), TS PEOE (R4), TS Pint (C5), TS Pint (H6), TS Pint (H7), TS Pint (N8), TS Pint (O10), TS Pint (O4), TS Pint (O9), TS SASA (C2), TS SASA (C5), TS SASA (H6), TS SASA (H7), TS SASA (N8), TS SASA (O10), TS SASA (O4), TS SASA (O9), TS SASA (R3), TS Softness, TS Sterimol B1 (R1), TS Sterimol B5 (R2), TS Sterimol L (R3), TS Sterimol B1 (R4), TS Sterimol B5 (R4), TS Vib. Freq., TS Vib. IR, TS ZPE

GPR, DFT MA (importances-0.5*mean)

- Train MAE: 0.96
- Test MAE: 1.01 ± 0.07
- Lit. 1 MAE: 1.21 ± 0.14
- Lit. 2 MAE: 1.46 ± 0.22
- Lit. 3 MAE: 1.36 ± 0.17
- Train R^2: 0.94
- Test R^2: 0.93
- Lit. 1 R^2: 0.01
- Lit. 2 R^2: -0.02
- Lit. 3 R^2: 0.14

Hyperparameters: GaussianProcessRegressor(alpha=1, kernel=1**2 * Matern(length_scale=1, nu=1.5))

Features (74): Chem. Pot., EState (C1), EState (C2), EState (C3), EState (O4), EState (R1), EState (R2), EState (R3), EState (R4), Electrophilicity, Enthalpy, QH Entropy, HB Acceptors, HB Donors, HOMO, Hardness, LUMO, LogP (O4), LogP (R2), LogP (R3), LogP (R4), MR (C1), MR (C2), MR (R1), MR (R2), MR (R3), MR (R4), Mulliken (C1), Mulliken (C2), Mulliken (C3), Mulliken (O4), Mulliken (R2), Mulliken (R3), Mulliken (R4), Amide Bonds, PBV (C1), PBV (C2), PBV (C3), PBV (O4), PBV (R1), PBV (R2), PBV (R4), PEOE (C1), PEOE (C2), PEOE (C3), PEOE (O4), PEOE (R1), PEOE (R2), PEOE (R3), PEOE (R4), Pint (C1), Pint (C2), Pint (C3), Pint (O4), Pint (R2), Pint (R4), SASA (C2), SASA (C3), SASA (O4), SASA (R1), SASA (R2), SASA (R3), SASA (R4), Sterimol B1 (R1), Sterimol B5 (R1), Sterimol B1 (R2), Sterimol B5 (R2), Sterimol L (R2), Sterimol B1 (R3), Sterimol L (R3), Sterimol B1 (R4), Sterimol L (R4), TPSA, Vib. IR

GPR, AM1 PM6 All (importances-0.5*mean)

- Train MAE: 0.96
- Test MAE: 0.93 ± 0.06
- Lit. 1 MAE: 1.02 ± 0.18
- Lit. 2 MAE: 1.17 ± 0.2
- Lit. 3 MAE: 1.11 ± 0.18
- Train R^2: 0.94
- Test R^2: 0.94
- Lit. 1 R^2: -0.01
- Lit. 2 R^2: 0.26
- Lit. 3 R^2: 0.28

Hyperparameters: GaussianProcessRegressor(alpha=0.01, kernel=1**2 * Matern(length_scale=1, nu=1.5))

Features (192): AM1 Barrier, AM1 MA Chem. Pot., AM1 MA Electrophilicity, AM1 MA Energy, AM1 MA Mulliken (C1), AM1 MA Mulliken (C2), AM1 MA Mulliken (O4), AM1 MA Mulliken (R1), AM1 MA Mulliken (R2), AM1 MA Mulliken (R3), AM1 MA PBV (R2), AM1 MA PBV (R4), AM1 MA Pint (C1), AM1 MA Pint (C2), AM1 MA Pint (C3), AM1 MA Pint (R2), AM1 MA Pint (R4), AM1 MA SASA (C1), AM1 MA SASA (C3), AM1 MA SASA (O4), AM1 MA SASA (R4), AM1 MA Sterimol B5 (R1), AM1 MA Sterimol B1 (R2), AM1 MA Sterimol B1 (R3), AM1 MA Sterimol B1 (R4), AM1 MA Sterimol B5 (R4), AM1 MA Sterimol L (R4), AM1 MA Vib. Freq., AM1 MA Vib. IR, AM1 TS Chem. Pot., AM1 TS Distance, AM1 TS EState (C2), AM1 TS EState (C3), AM1 TS Electrophilicity, AM1 TS Energy (SPE), AM1 TS Enthalpy, AM1 TS HOMO, AM1 TS LUMO, AM1 TS LogP (C1), AM1 TS Mulliken (C1), AM1 TS Mulliken (C2), AM1 TS Mulliken (C3), AM1 TS Mulliken (C5), AM1 TS Mulliken (H6), AM1 TS Mulliken (H7), AM1 TS Mulliken (N8), AM1 TS Mulliken (O10), AM1 TS Mulliken (O4), AM1 TS Mulliken (O9), AM1 TS Mulliken (R3), AM1 TS Mulliken (R4), AM1 TS PBV (C1), AM1 TS PBV (C2), AM1 TS PBV (C3), AM1 TS PBV (C5), AM1 TS PBV (H6), AM1 TS PBV (H7), AM1 TS PBV (N8), AM1 TS PBV (O4), AM1 TS PBV (R3), AM1 TS PEOE (C1), AM1 TS PEOE (C2), AM1 TS PEOE (C3), AM1 TS PEOE (O4), AM1 TS PEOE (R2), AM1 TS PEOE (R3), AM1 TS PEOE (R4), AM1 TS Pint (C5), AM1 TS Pint (H6), AM1 TS Pint (N8), AM1 TS Pint (O10), AM1 TS Pint (O4), AM1 TS Pint (O9), AM1 TS SASA (C2), AM1 TS SASA (C5), AM1 TS SASA (H6), AM1 TS SASA (H7), AM1 TS SASA (N8), AM1 TS SASA (O4), AM1 TS SASA (O9), AM1 TS SASA (R1), AM1 TS Sterimol B1 (R1), AM1 TS Sterimol L (R1), AM1 TS Sterimol B5 (R2), AM1 TS Sterimol B1 (R3), AM1 TS Sterimol B5 (R3), AM1 TS Sterimol L (R3), AM1 TS Sterimol B1 (R4), AM1 TS Sterimol B5 (R4), AM1 TS Vib. Freq., PM6 Barrier, PM6 MA Chem. Pot., PM6 MA EState (C1), PM6 MA QH Entropy, PM6 MA LUMO, PM6 MA Mulliken (O4), PM6 MA Mulliken (R1), PM6 MA Mulliken (R2), PM6 MA Mulliken (R3), PM6 MA PBV (C1), PM6 MA PBV (O4), PM6 MA Pint (C1), PM6 MA Pint (C2), PM6 MA Pint (C3), PM6 MA Pint (R1), PM6 MA Pint (R2), PM6 MA Pint (R3), PM6 MA Pint (R4), PM6 MA SASA (C1), PM6 MA SASA (C3), PM6 MA SASA (O4), PM6 MA SASA (R1), PM6 MA SASA (R2), PM6 MA SASA (R4), PM6 MA Sterimol B1 (R2), PM6 MA Sterimol B1

(R3), PM6 MA Sterimol B5 (R3), PM6 MA Sterimol B1 (R4), PM6 MA Sterimol B5 (R4), PM6 MA Sterimol L (R4), PM6 MA TPSA, PM6 MA Vib. Freq., PM6 MA Vib. IR, PM6 TS Chem. Pot., PM6 TS Distance, PM6 TS EState (C2), PM6 TS EState (C3), PM6 TS EState (O4), PM6 TS EState (R1), PM6 TS EState (R2), PM6 TS EState (R3), PM6 TS EState (R4), PM6 TS Energy, PM6 TS Enthalpy, PM6 TS HB Acceptors, PM6 TS HB Donors, PM6 TS HOMO, PM6 TS LUMO, PM6 TS LogP (R1), PM6 TS LogP (R2), PM6 TS LogP (R3), PM6 TS LogP (R4), PM6 TS MR (C1), PM6 TS MR (R1), PM6 TS MR (R2), PM6 TS MR (R3), PM6 TS MR (R4), PM6 TS Mulliken (C1), PM6 TS Mulliken (C2), PM6 TS Mulliken (C5), PM6 TS Mulliken (H6), PM6 TS Mulliken (H7), PM6 TS Mulliken (N8), PM6 TS Mulliken (O4), PM6 TS Mulliken (O9), PM6 TS Mulliken (R4), PM6 TS Amide Bonds, PM6 TS PBV (C2), PM6 TS PBV (C5), PM6 TS PBV (H6), PM6 TS PBV (H7), PM6 TS PBV (N8), PM6 TS PBV (O10), PM6 TS PBV (O9), PM6 TS PBV (R1), PM6 TS PBV (R3), PM6 TS PEOE (C1), PM6 TS PEOE (C2), PM6 TS PEOE (R2), PM6 TS Pint (C5), PM6 TS Pint (H6), PM6 TS Pint (H7), PM6 TS Pint (N8), PM6 TS Pint (O10), PM6 TS Pint (O9), PM6 TS SASA (C2), PM6 TS SASA (C5), PM6 TS SASA (H6), PM6 TS SASA (H7), PM6 TS SASA (N8), PM6 TS SASA (O4), PM6 TS SASA (O9), PM6 TS SASA (R3), PM6 TS Softness, PM6 TS Sterimol B1 (R1), PM6 TS Sterimol B5 (R2), PM6 TS Sterimol B1 (R3), PM6 TS Sterimol L (R3), PM6 TS Sterimol B1 (R4), PM6 TS Sterimol B5 (R4), PM6 TS Vib. Freq., PM6 TS Vib. IR

6. Learning Curves

The learning curves for each SVR and KRR model with the AM1 All feature subset are provided in Fig. S8-11. The train and test scores for each model tend to be very close to one another, indicating that no significant overfitting takes place

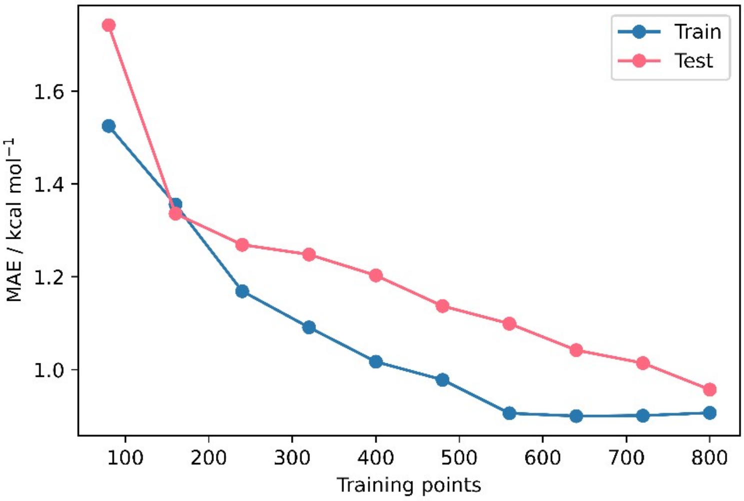

in the models at any point.

Fig. S8. MAE learning curves for SVR(RBF)(AM1 All).

Fig. S9. MAE learning curves for SVR(Poly)(AM1 All).

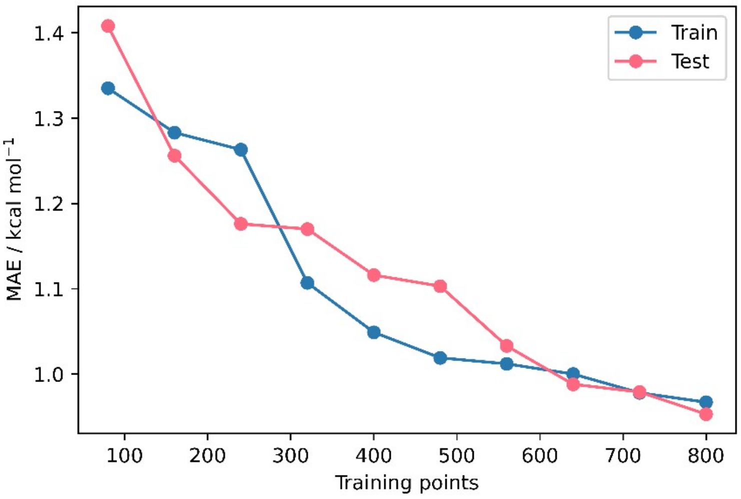

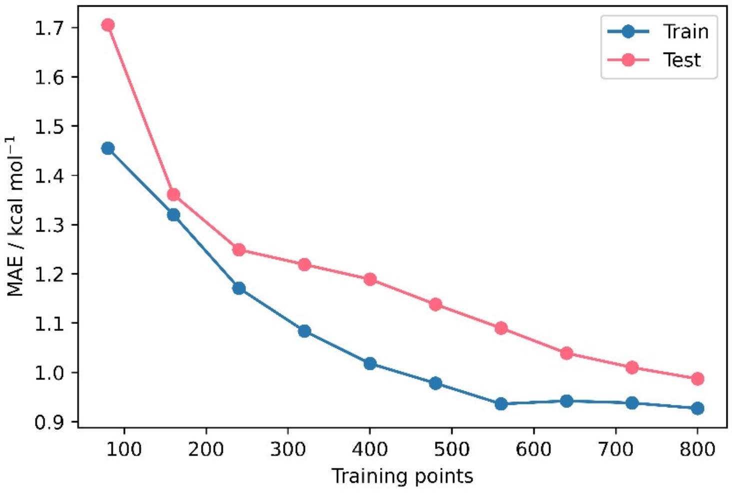

Fig. S10. MAE learning curves for KRR(RBF)(AM1 All).

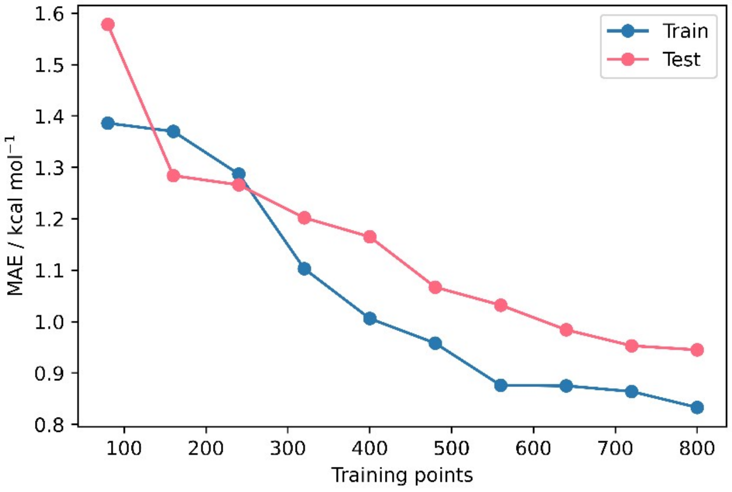

Fig. S11. MAE learning curves for KRR(Poly)(AM1 All).

7. Feature Importances

The top 15 train, test, and literature (set 1) feature importances for each GPR model with the PM6 All and AM1 + PM6 All feature subsets are provided in Fig. S12-13. The top 15 train, test, and literature (set 1) feature importances for each SVR and KRR model with the AM1 All feature subset are provided in Fig. S14-17. In each case, the SQM barrier and Mulliken charge (O4) tend to dominate the features importances, followed by several electrostatic (Mulliken charge, PEOE, P_{int}, EState) and steric (sterimol, PBV, SASA) properties pertaining largely to the R_1-R_4 substituents and core Michael acceptor atoms (C_1-O_4). However, for PM6-derived feature subsets, the SQM barrier is seen to be less impactful than the AM1 barrier. In general, there is consistency amongst the features that appear in each set of feature importances, indicating that the models are generalisable and not overfitted.

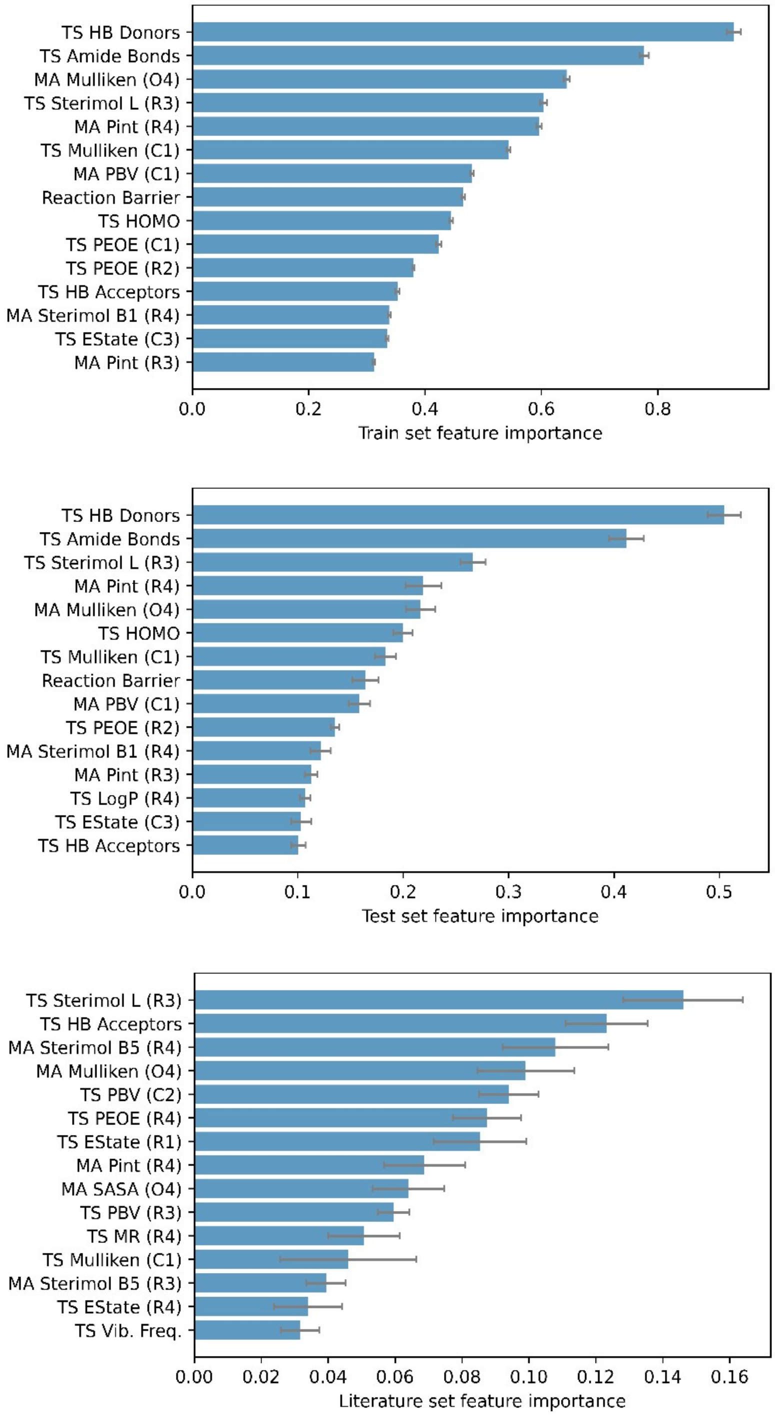

Fig. S12. Top 15 train, test, and literature permutation features importances for GPR(PM6 All).

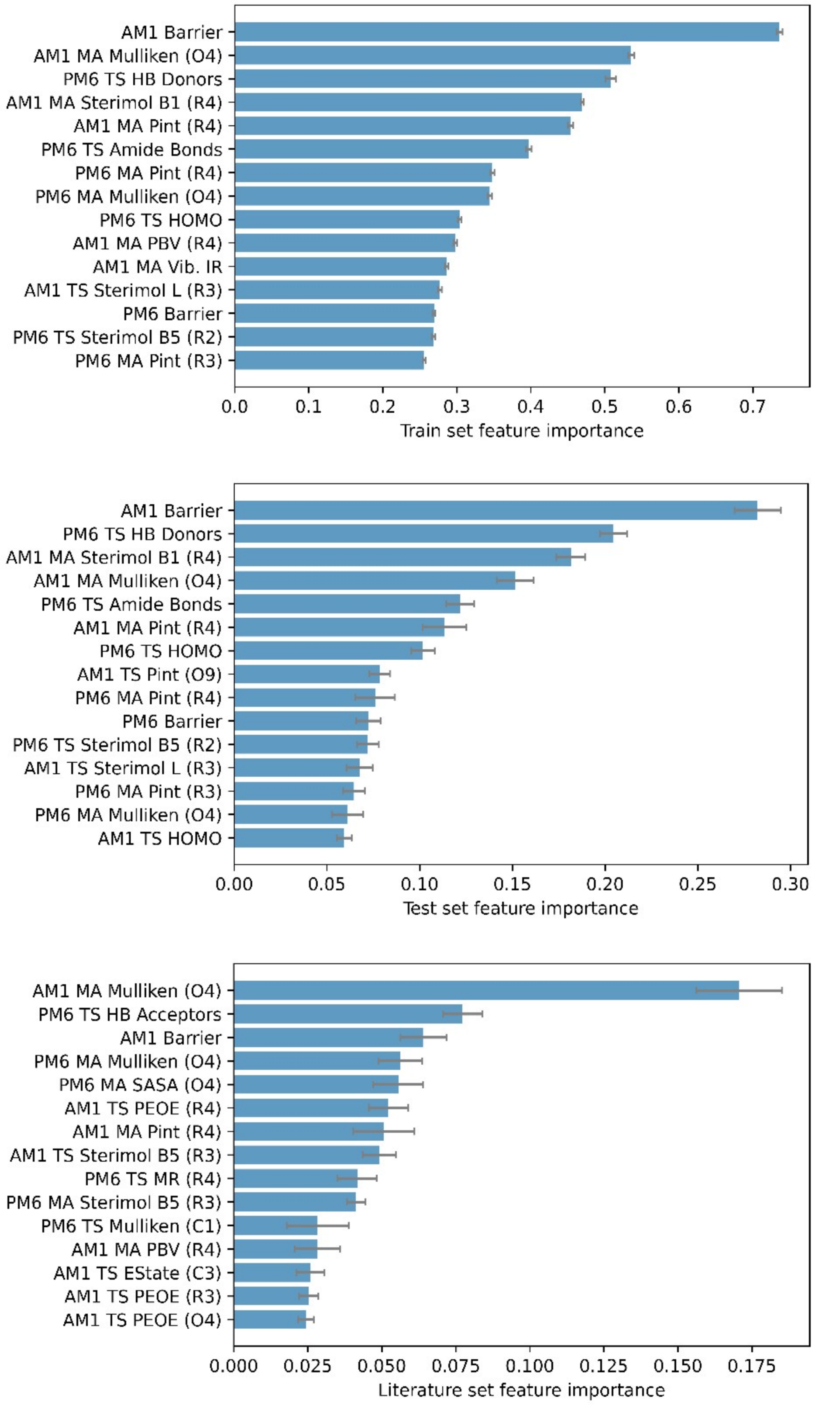

Fig. S13. Top 15 train, test, and literature permutation features importances for GPR(AM1 + PM6 All).

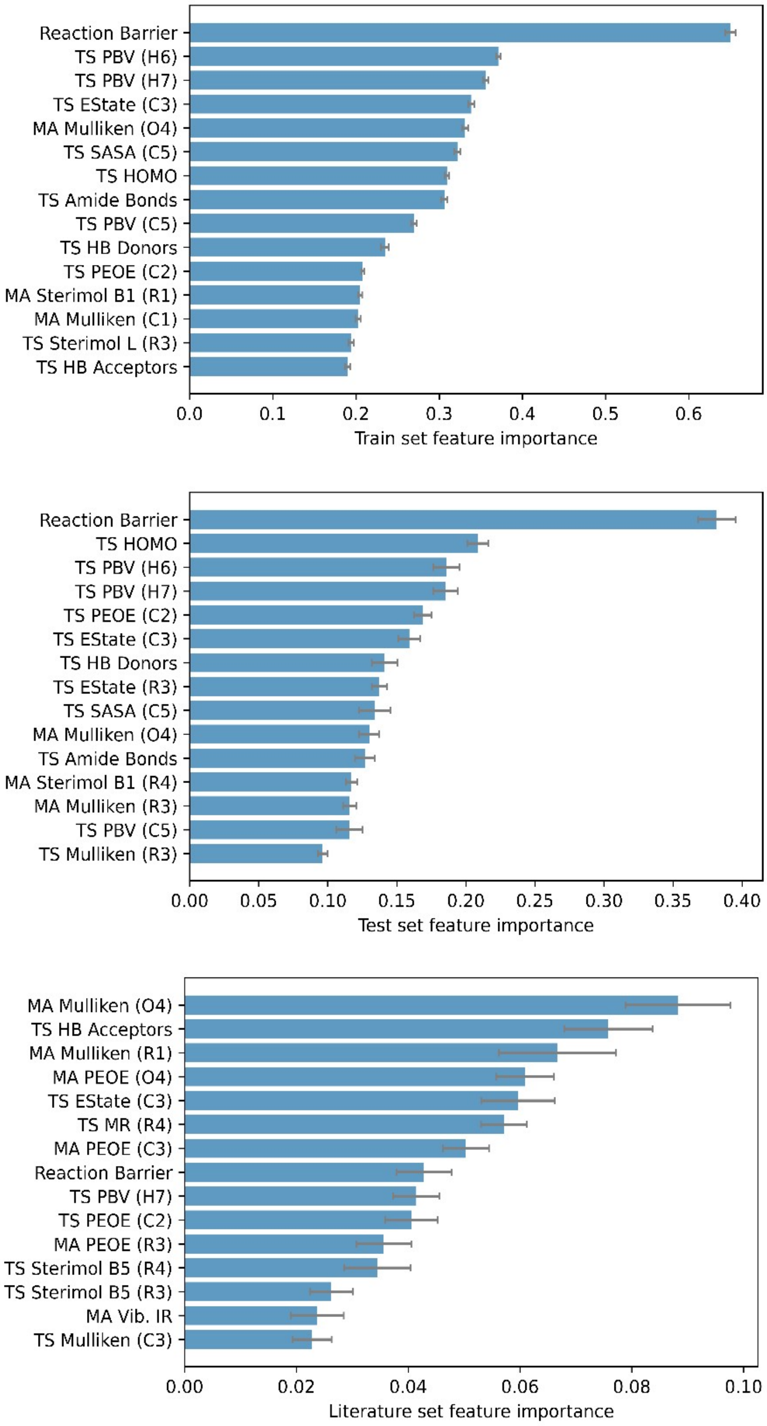

Fig. S14. Top 15 train, test, and literature permutation features importances for SVR(RBF)(AM1 All).

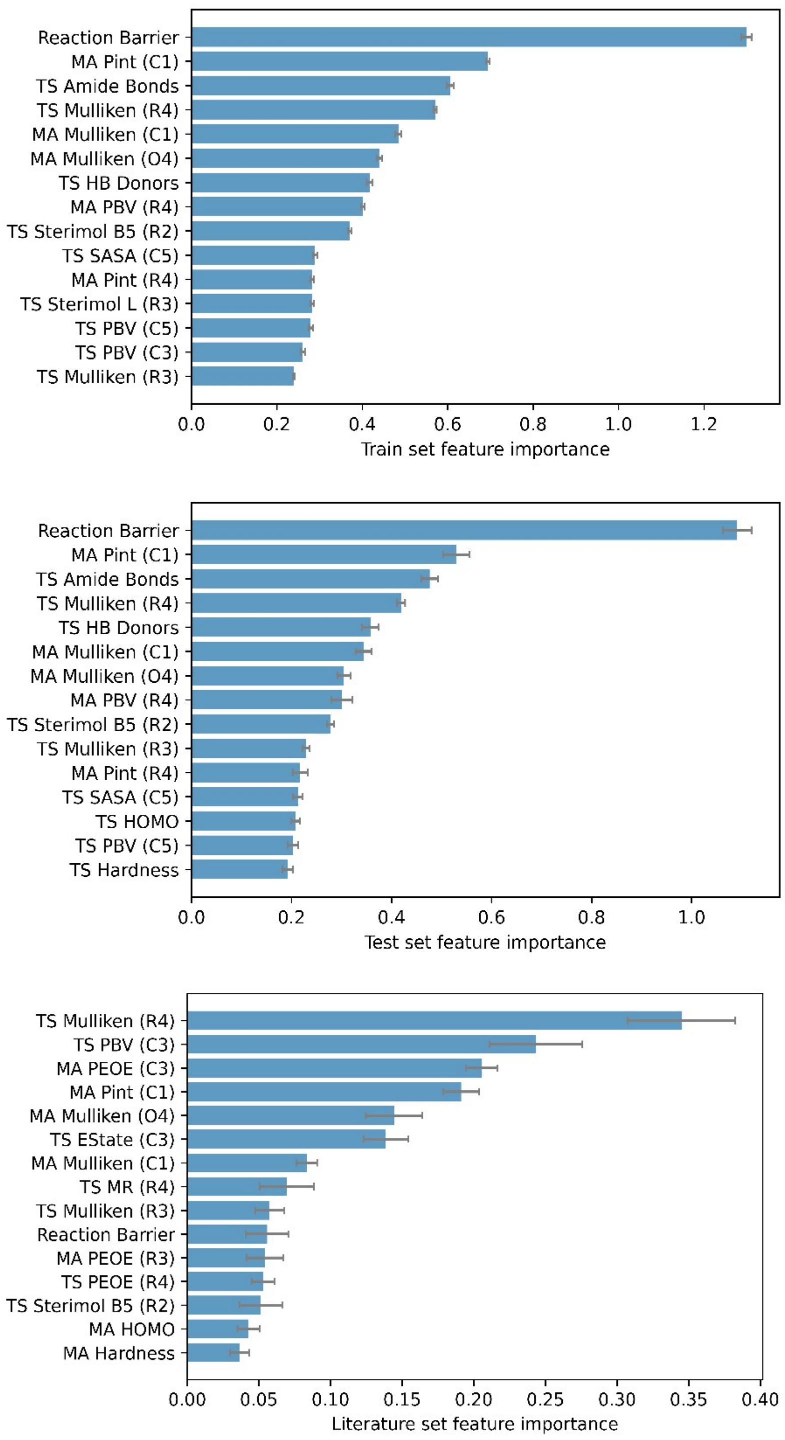

Fig. S15. Top 15 train, test, and literature permutation features importances for SVR(Poly)(AM1 All).

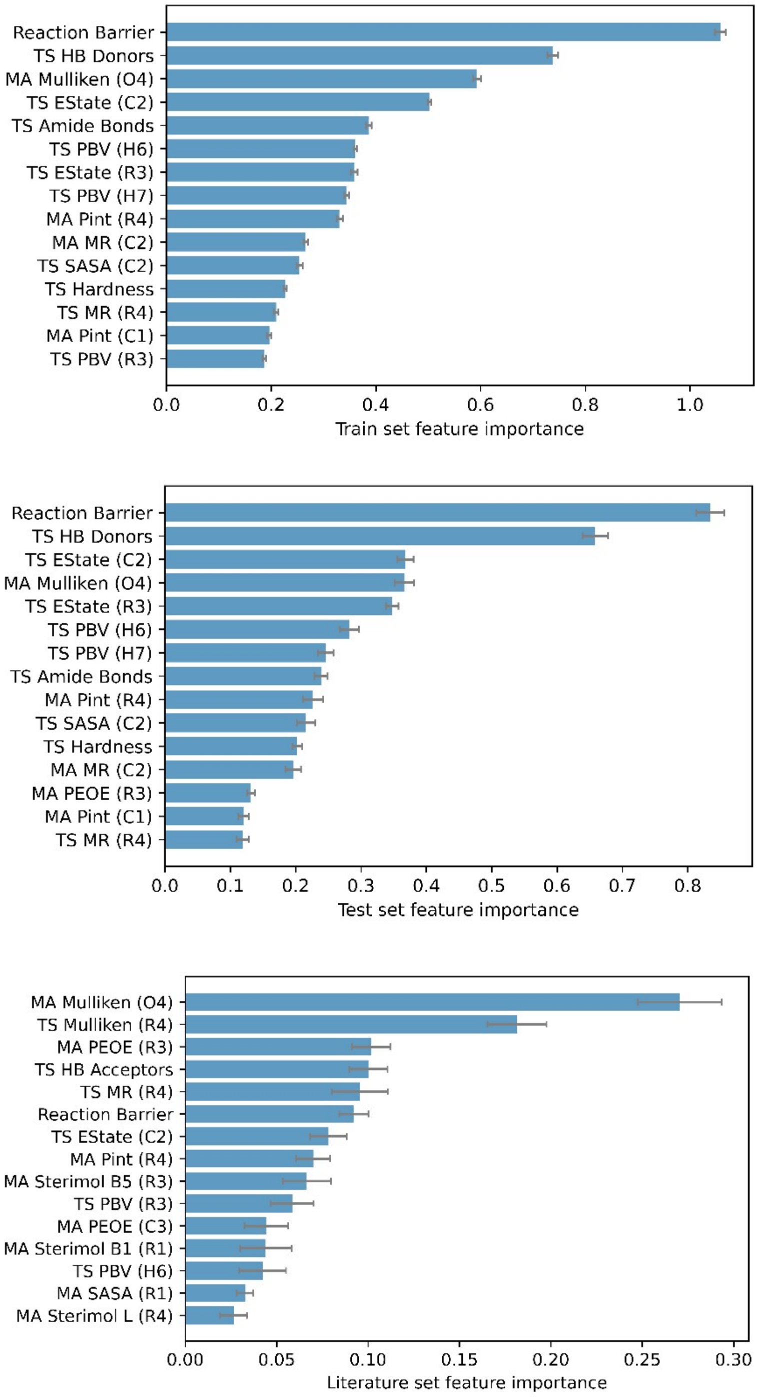

Fig. S16. Top 15 train, test, and literature permutation features importances for KRR(RBF)(AM1 All).

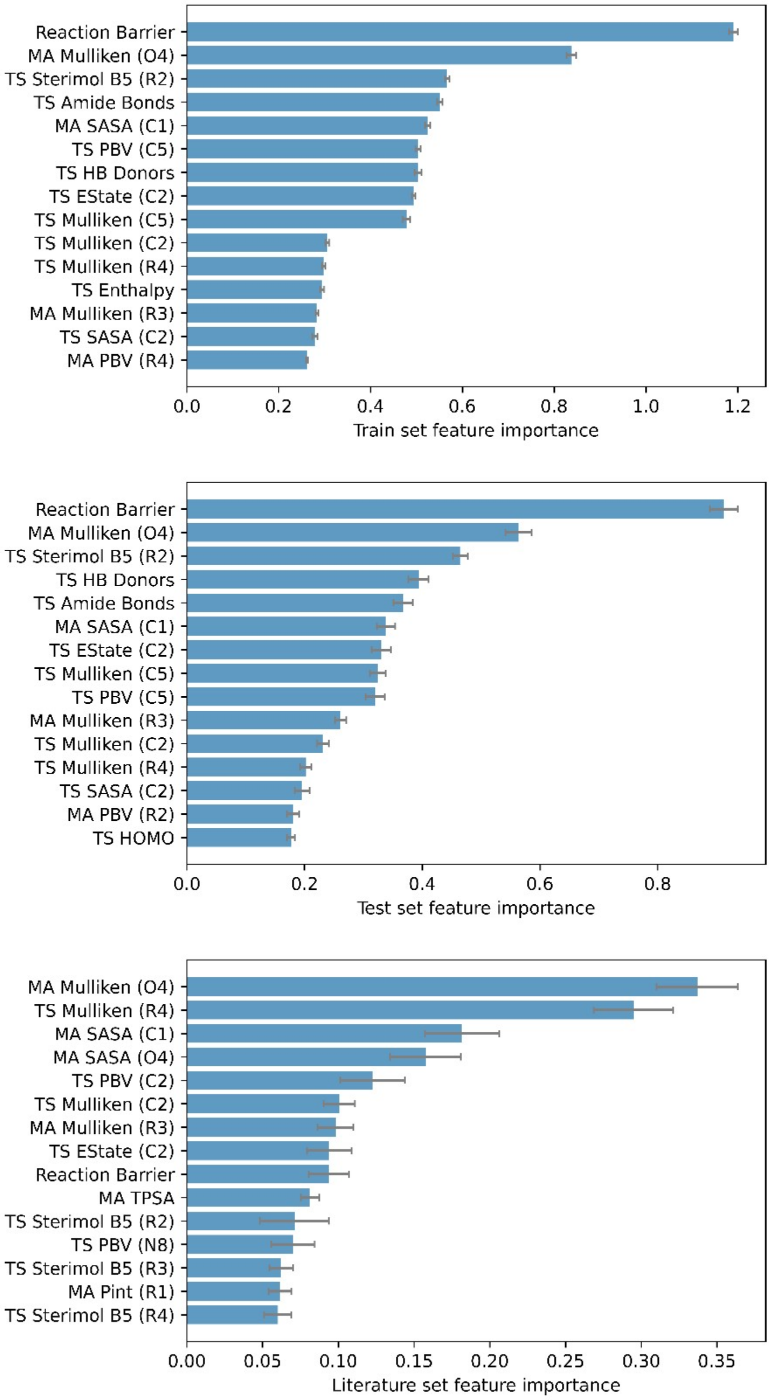

Fig. S17. Top 15 train, test, and literature permutation features importances for KRR(Poly)(AM1 All).

8. RMSD Analyses

Root-mean-squared-deviations of atomic positions (RMSDs) were calculated to assess the similarity between geometries of the MAs and TSs at each level of theory compared to the DFT TSs. The results of these analyses are summarised in Fig. S18-28. All RMSDs were calculated via a quaternion-based characteristic polynomial method[65] with the spyrmsd python package.[66] Structures being compared (all atoms, including hydrogens) were superimposed prior to calculation of each RMSD.

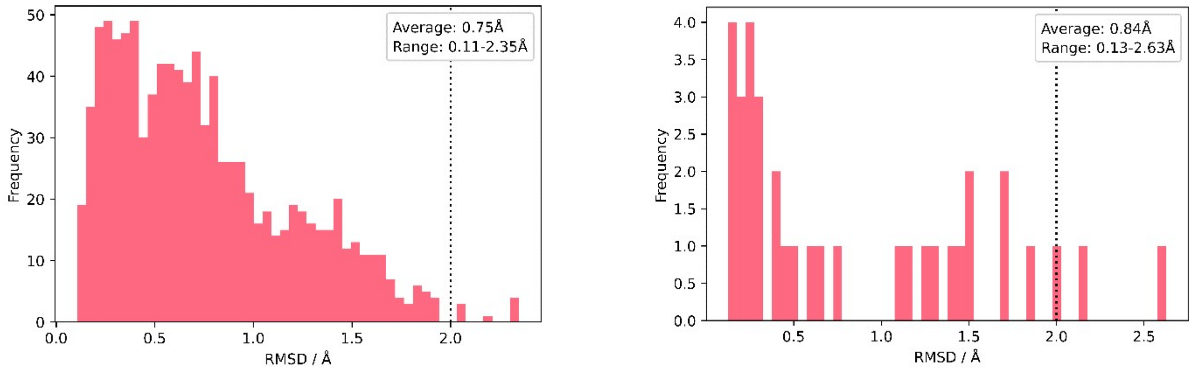

Fig. S18. Distribution of RMSDs between each TS at its AM1 and ωB97X-D/def2-TZVP geometries for the enumerated dataset (left) and literature set 1 (right).

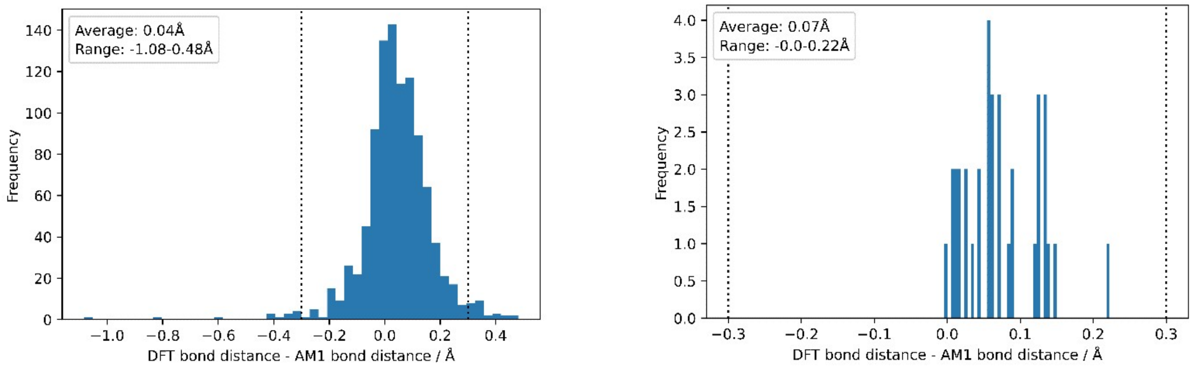

Fig. S19. Distribution of bond distance differences between each TS at its AM1 and ωB97X-D/def2-TZVP geometries for the enumerated dataset (left) and literature set 1 (right).

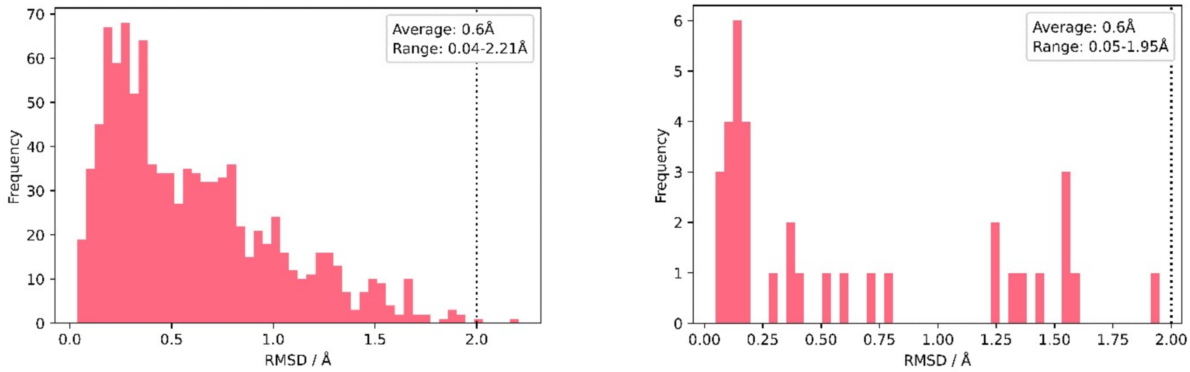

Fig. S20. Distribution of RMSDs between each TS (nucleophile removed) at its AM1 and ωB97X-D/def2-TZVP geometries for the enumerated dataset (left) and literature set 1 (right).

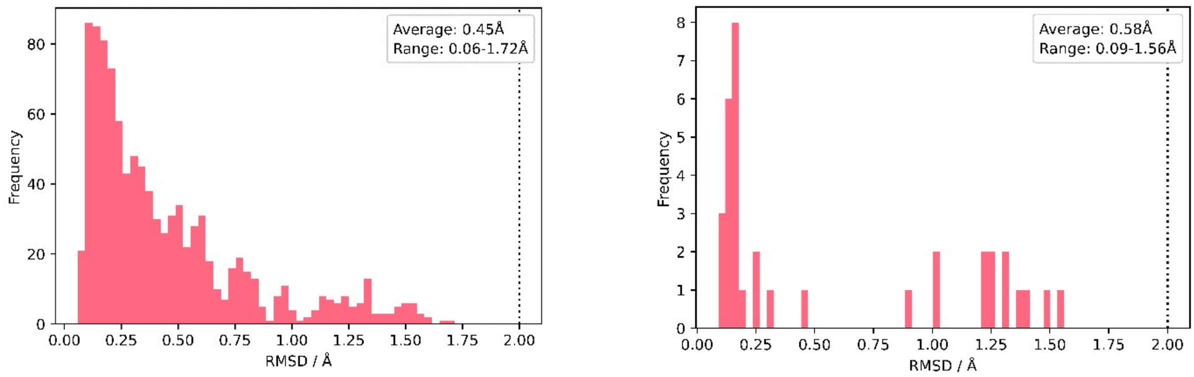

Fig. S21. Distribution of RMSDs between each TS (R-groups removed) at its AM1 and ωB97X-D/def2-TZVP

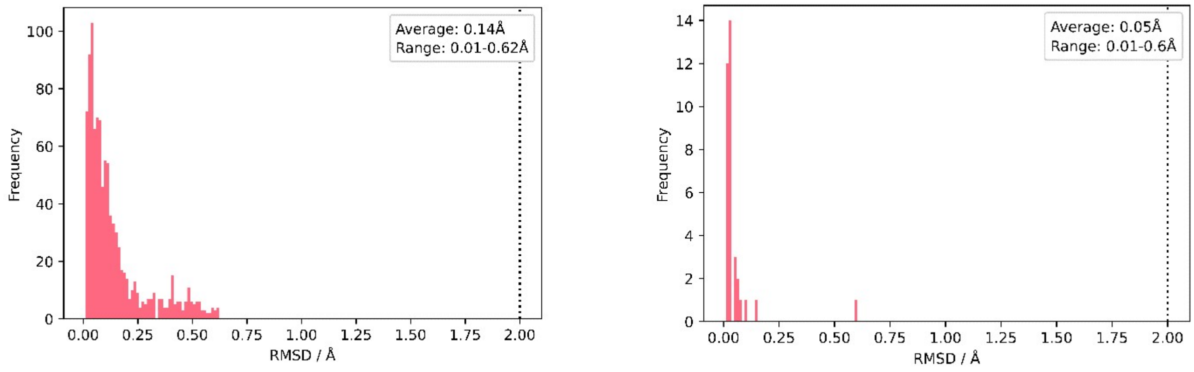

geometries for the enumerated dataset (left) and literature set 1 (right).

Fig. S22. Distribution of RMSDs between each TS (nucleophile and R-groups removed) at its AM1 and ωB97X-D/def2-TZVP geometries for the enumerated dataset (left) and literature set 1 (right).

Fig. S23. Distribution of RMSDs between each MA at its AM1 geometry and each TS (nucleophile removed) at its ωB97X-D/def2-TZVP geometry for the enumerated dataset (left) and literature set 1 (right).

Fig. S24. Distribution of RMSDs between each MA at its UFF geometry and each TS (nucleophile removed) at its ωB97X-D/def2-TZVP geometry for the enumerated dataset (left) and literature set 1 (right).

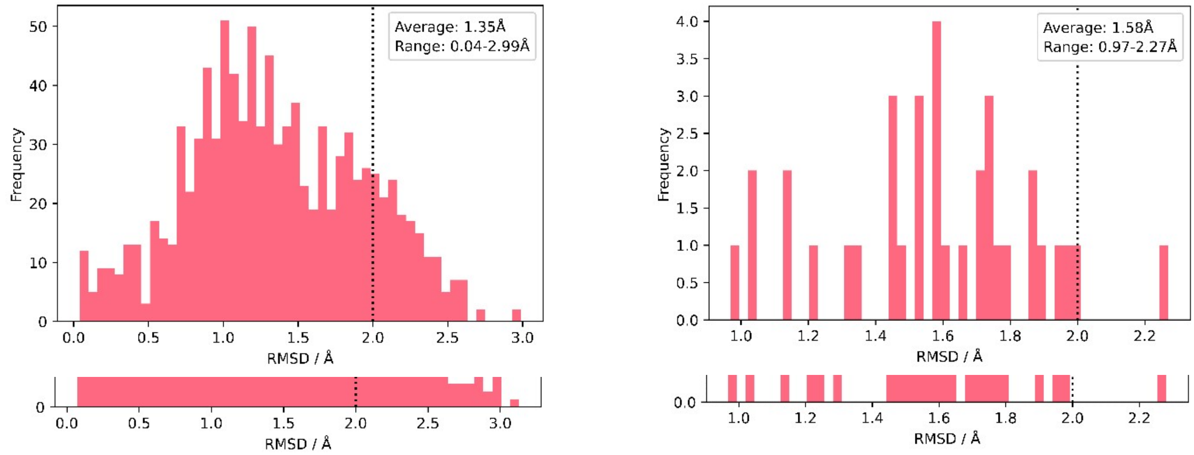

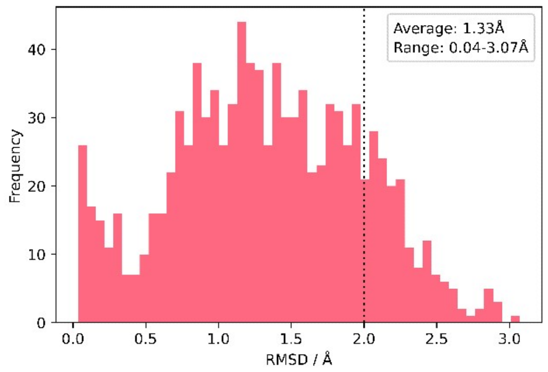

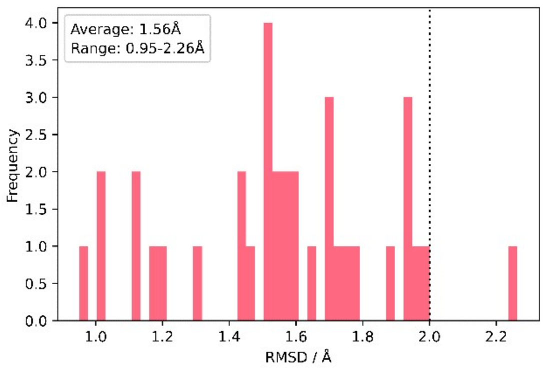

Fig. S25. Distribution of RMSDs between each MA at its ωB97X-D/def2-TZVP geometry and each TS (nucleophile

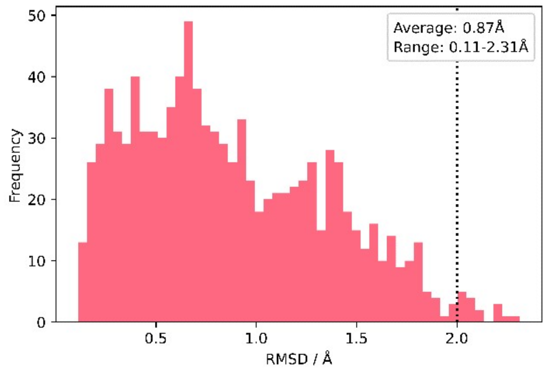

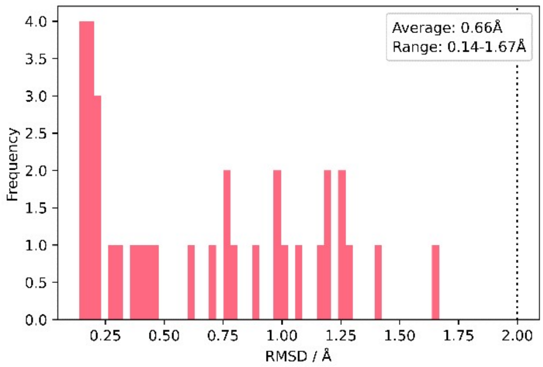

removed) at its ωB97X-D/def2-TZVP geometry for the enumerated dataset (left) and literature set 1 (right).

Fig. S26. Distribution of RMSDs between each TS at its PM6 and ωB97X-D/def2-TZVP geometries for the enumerated dataset (left) and literature set 1 (right).

Fig. S27. Distribution of RMSDs between each literature set 1 TS at its AM1 and AM1/IEFPCM(toluene) geometries (left) and PM6 and PM6/IEFPCM(toluene) geometries (right).

Fig. S28. Distribution of RMSDs between each literature set 1 TS at its ωB97X-D/def2-TZVP and ωB97X-D/def2-TZVP /IEFPCM(toluene) geometries.

9. References

1 Schrödinger Release 2020-1: Maestro, Schrödinger, LLC, New York, NY, 2020.

2 Q. Gu and S. L. You, *Chem. Sci.*, 2011, **2**, 1519–1522.

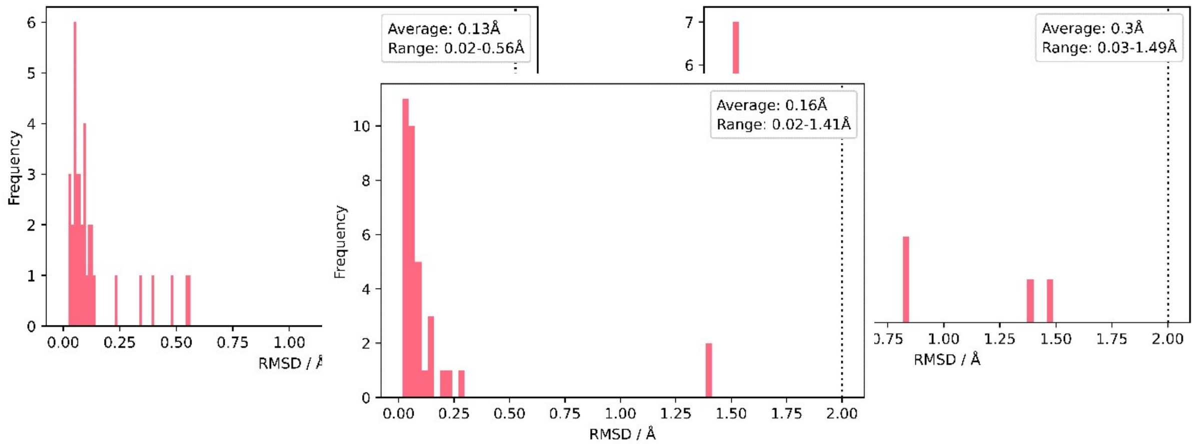

3 A. Böhme, A. Laqua and G. Schüürmann, *Chem. Res. Toxicol.*, 2016, **29**, 952–962.

4 P. A. Jackson, J. C. Widen, D. A. Harki and K. M. Brummond, *J. Med. Chem.*, 2017, **60**, 839–885.

5 J. A. H. Schwöbel, D. Wondrousch, Y. K. Koleva, J. C. Madden, M. T. D. Cronin and G. Schüürmann, *Chem. Res. Toxicol.*, 2010, **23**, 1576–1585.

6 S. Grimme, *Chem. - A Eur. J.*, 2012, **18**, 9955–9964.

7 G. Luchini, J. V. Alegre-Requena, I. Funes-Ardoiz and R. S. Paton, *F1000Research*, 2020, **9**, 291.

8 I. M. Alecu, J. Zheng, Y. Zhao and D. G. Truhlar, *J. Chem. Theory Comput.*, 2010, **6**, 2872–2887.

9 R. D'Agostino and E. S. Pearson, *Biometrika*, 1973, **60**, 613.

10 P. Virtanen, R. Gommers, T. E. Oliphant, M. Haberland, T. Reddy, D. Cournapeau, E. Burovski, P. Peterson, W. Weckesser, J. Bright, S. J. van der Walt, M. Brett, J. Wilson, K. J. Millman, N. Mayorov, A. R. J. Nelson, E. Jones, R. Kern, E. Larson, C. J. Carey, İ. Polat, Y. Feng, E. W. Moore, J. VanderPlas, D. Laxalde, J. Perktold, R. Cimrman, I. Henriksen, E. A. Quintero, C. R. Harris, A. M. Archibald, A. H. Ribeiro, F. Pedregosa, P. van Mulbregt, A. Vijaykumar, A. Pietro Bardelli, A. Rothberg, A. Hilboll, A. Kloeckner, A. Scopatz, A. Lee, A. Rokem, C. N. Woods, C. Fulton, C. Masson, C. Häggström, C. Fitzgerald, D. A. Nicholson, D. R. Hagen, D. V. Pasechnik, E. Olivetti, E. Martin, E. Wieser, F. Silva, F. Lenders, F. Wilhelm, G. Young, G. A. Price, G.-L. Ingold, G. E. Allen, G. R. Lee, H. Audren, I. Probst, J. P. Dietrich, J. Silterra, J. T. Webber, J. Slavič, J. Nothman, J. Buchner, J. Kulick, J. L. Schönberger, J. V. de Miranda Cardoso, J. Reimer, J. Harrington, J. L. C. Rodríguez, J. Nunez-Iglesias, J. Kuczynski, K. Tritz, M. Thoma, M. Newville, M. Kümmerer, M. Bolingbroke, M. Tartre, M. Pak, N. J. Smith, N. Nowaczyk, N. Shebanov, O. Pavlyk, P. A. Brodtkorb, P. Lee, R. T. McGibbon, R. Feldbauer, S. Lewis, S. Tygier, S. Sievert, S. Vigna, S. Peterson, S. More, T. Pudlik, T. Oshima, T. J. Pingel, T. P. Robitaille, T. Spura, T. R. Jones, T. Cera, T. Leslie, T. Zito, T. Krauss, U. Upadhyay, Y. O. Halchenko and Y. Vázquez-Baeza, *Nat. Methods*, 2020, **17**, 261–272.

11 F. Mohamadi, N. G. J. Richards, W. C. Guida, R. Liskamp, M. Lipton, C. Caufield, G. Chang, T. Hendrickson and W. C. Still, *J. Comput. Chem.*, 1990, **11**, 440–467.

12 Schrödinger Macromodel version 12.7, Schrödinger, LLC, New York, NY, 2020.

13 K. Roos, C. Wu, W. Damm, M. Reboul, J. M. Stevenson, C. Lu, M. K. Dahlgren, S. Mondal, W. Chen, L. Wang, R. Abel, R. A. Friesner and E. D. Harder, *J. Chem. Theory Comput.*, 2019, **15**, 1863–1874.

14 T. Lewis-Atwell, P. A. Townsend and M. N. Grayson, *J. Org. Chem.*, 2022, **87**, 5703–5712.

15 G. Chang, W. C. Guida and W. C. Still, *J. Am. Chem. Soc.*, 1989, **111**, 4379–4386.

16 I. Kolossváry and W. C. Guida, *J. Am. Chem. Soc.*, 1996, **118**, 5011–5019.

17 I. Kolossvary and W. C. Guida, *J. Comput. Chem.*, 1999, **20**, 1671–1684.

18 M. J. Frisch, G. W. Trucks, H. B. Schlegel, G. E. Scuseria, M. A. Robb, J. R. Cheeseman, G. Scalmani, V. Barone, B. Mennucci, G. A. Petersson, H. Nakatsuji, M. Caricato, X. Li, H. P. Hratchian, A. F. Izmaylov, J. Bloino, J. Zheng, J. L. Sonnenberg, M. Hada, M. Ehara, K. Toyota, R. Fukuda, J. Hasegawa, M. Ishida, T. Nakajima, Y. Honda, O. Kitao, H. Nakai, T. Vreven, J. A. Montgomery, J. E. Peralta, F. Ogliaro, M. Bearpark, J. J. Heyd, E. Brothers, K. N. Kudin, V. N. Staroverov, R. Kobayashi, J. Normand, K. Raghavachari, J. C. A. Rendell, S. Burant, S. Iyengar, J. Tomasi, M. Cossi, N. Rega, J. M. Millam, M. Klene, J. E. Knox, J. B. Cross, V. Bakken, C. Adamo, J. Jaramillo, R. Gomperts, R. E. Stratmann, O. Yazyev, A. J. Austin, R. Cammi, C. Pomelli, J. W. Ochterski, R. L. Martin, K. Morokuma, V. G. Zakrzewski, G. A. Voth, P. Salvador, J. J. Dannenberg, S. Dapprich, A. D. Daniels, O. Farkas, J. B. Foresman, J. V. Ortiz, J. Cioslowski and D. J. Fox, 2016.

19 A. K. Rappé, C. J. Casewit, K. S. Colwell, W. A. Goddard and W. M. Skiff, *J. Am. Chem. Soc.*, 1992, **114**, 10024–10035.

20 M. J. S. Dewar, E. G. Zoebisch, E. F. Healy and J. J. P. Stewart, *J. Am. Chem. Soc.*, 1985, **107**, 3902–3909.

21 J. J. P. Stewart, *J. Mol. Model.*, 2007, **13**, 1173–1213.

22 Y.-Y. Wu, F.-Q. Zhao and X.-H. Ju, *J. Mex. Chem. Soc.*, 2014, **58**, 223–229.

23 J. J. P. Stewart, *J. Mol. Model.*, 2013, **19**, 1–32.

24 J.-D. Chai and M. Head-Gordon, *Phys. Chem. Chem. Phys.*, 2008, **10**, 6615.

25 F. Weigend and R. Ahlrichs, *Phys. Chem. Chem. Phys.*, 2005, **7**, 3297.

26 N. Mardirossian and M. Head-Gordon, *Mol. Phys.*, 2017, **115**, 2315–2372.

27 L. Goerigk and S. Grimme, *Wiley Interdiscip. Rev. Comput. Mol. Sci.*, 2014, **4**, 576–600.

28 C. A. Grambow, L. Pattanaik and W. H. Green, *Sci. Data*, 2020, **7**, 137.

29 L. Simón and J. M. Goodman, *Org. Biomol. Chem.*, 2011, **9**, 689–700.

30 B. Mennucci, R. Cammi and J. Tomasi, *J. Chem. Phys.*, 1998, **109**, 2798–2807.

31 B. Vakulya, S. Varga, A. Csámpai and T. Soós, *Org. Lett.*, 2005, **7**, 1967–1969.

32 W. Yang and D. M. Du, *Org. Lett.*, 2010, **12**, 5450–5453.

33 CYLView1.0b, C. Y. Legault, 2009.

34 F. Pedregosa, G. Varoquaux, A. Gramfort, V. Michel, B. Thirion, O. Grisel, M. Blondel, P. Prettenhofer, R. Weiss, V. Dubourg, J. Vanderplas, A. Passos, D. Cournapeau, M. Brucher, M. Perrot and É. Duchesnay, *J. Mach. Learn. Res.*, 2011, **12**, 2825–2830.

35 K. N. Houk and F. Liu, *Acc. Chem. Res.*, 2017, **50**, 539–543.

36 K. A. Peterson, D. Feller and D. A. Dixon, *Theor. Chem. Acc.*, 2012, **131**, 1–20.

37 N. M. O'Boyle, C. Morley and G. R. Hutchison, *Chem. Cent. J.*, 2008, **2**, 5.

38 RDKit: Open-source cheminformatics, version 2020.03.2.0, 2020.

39 R. M. LoPachin, T. Gavin, A. DeCaprio and D. S. Barber, *Chem. Res. Toxicol.*, 2012, **25**, 239–251.

40 N. M. O'boyle, A. L. Tenderholt and K. M. Langner, *J. Comput. Chem.*, 2008, **29**, 839–845.

41 S. Mitternacht, *F1000Research*, 2016, **5**, 189.

42 P. Ertl, B. Rohde and P. Selzer, *J. Med. Chem.*, 2000, **43**, 3714–3717.

43 Morfeus: molecular features for machine learning, version 2021.0.6.0, 2021.

44 R. Pollice and P. Chen, *Angew. Chemie - Int. Ed.*, 2019, **58**, 9758–9769.

45 A. Poater, B. Cosenza, A. Correa, S. Giudice, F. Ragone, V. Scarano and L. Cavallo, *Eur. J. Inorg. Chem.*, 2009, **2009**, 1759–1766.

46 L. Falivene, R. Credendino, A. Poater, A. Petta, L. Serra, R. Oliva, V. Scarano and L. Cavallo, *Organometallics*, 2016, **35**, 2286–2293.

47 A. Verloop, *Drug Design, Vol III*, Academic Press, New York, 1976.

48 J. Gasteiger and M. Marsili, *Tetrahedron*, 1980, **36**, 3219–3228.

49 S. A. Wildman and G. M. Crippen, *J. Chem. Inf. Comput. Sci.*, 1999, **39**, 868–873.

50 L. H. Hall and L. B. Kier, *J. Chem. Inf. Comput. Sci.*, 1995, **35**, 1039–1045.

51 P. Labute, *J. Mol. Graph. Model.*, 2000, **18**, 464–477.

52 J. W. Osborne and E. Waters, *Pract. Assessment, Res. Eval.*, 2002, **8**.

53 K. Jorner, T. Brinck, P. O. Norrby and D. Buttar, *Chem. Sci.*, 2021, **12**, 1163–1175.

54 D. M. Hawkins, *J. Chem. Inf. Comput. Sci.*, 2004, **44**, 1–12.

55 S. Raschka, *J. Open Source Softw.*, 2018, **3**, 638.

56 A. E. Hoerl and R. W. Kennard, *Technometrics*, 1970, **12**, 55–67.

57 N. S. Altman, *Am. Stat.*, 1992, **46**, 175–185.

58 C. Cortes and V. Vapnik, *Mach. Learn.*, 1995, **20**, 273–297.

59 L. Breiman, *Mach. Learn.*, 2001, **45**, 5–32.

60 J. H. Friedman, *Ann. Stat.*, 2001, **29**, 1189–1232.

61 H. Drucker, C. J. C. Surges, L. Kaufman, A. Smola and V. Vapnik, *Adv. Neural Inf. Process. Syst.*, 1997, **1**, 155–161.

62 V. N. Vapnik, in *Empirical Inference*, Springer, 2013, pp. 105–116.

63 C. K. I. Williams and C. E. Rasmussen, *Gaussian Processes for Regression*, MIT Press, 2006.

64 D. G. Jenkins and P. F. Quintana-Ascencio, *PLoS One*, 2020, **15**, e0229345.

65 D. L. Theobald, *Acta Crystallogr. Sect. A Found. Crystallogr.*, 2005, **61**, 478–480.

66 R. Meli and P. C. Biggin, *J. Cheminform.*, 2020, **12**, 49.
